# Three-dimensional Epigenome Statistical Model: Genome-wide Chromatin Looping Prediction

**DOI:** 10.1038/s41598-018-23276-8

**Published:** 2018-03-26

**Authors:** Ziad Al Bkhetan, Dariusz Plewczynski

**Affiliations:** 1 Centre of New Technologies, University of Warsaw, Warsaw, Poland; 2 Faculty of Mathematics and Information Science, Warsaw University of Technology, Warsaw, Poland; 3 Biology Department, University of Warsaw, Warsaw, Poland

## Abstract

This study aims to understand through statistical learning the basic biophysical mechanisms behind three-dimensional folding of epigenomes. The 3DEpiLoop algorithm predicts three-dimensional chromatin looping interactions within topologically associating domains (TADs) from one-dimensional epigenomics and transcription factor profiles using the statistical learning. The predictions obtained by 3DEpiLoop are highly consistent with the reported experimental interactions. The complex signatures of epigenomic and transcription factors within the physically interacting chromatin regions (anchors) are similar across all genomic scales: genomic domains, chromosomal territories, cell types, and different individuals. We report the most important epigenetic and transcription factor features used for interaction identification either shared, or unique for each of sixteen (16) cell lines. The analysis shows that CTCF interaction anchors are enriched by transcription factors yet deficient in histone modifications, while the opposite is true in the case of RNAP II mediated interactions. The code is available at the repository https://bitbucket.org/4dnucleome/3depiloop.

## Introduction

Understanding the biological function of the genome requires interrogation of two distinct aspects of Human genome organization. The first aspect is the one-dimensional genomic structure, the position of genes, regulatory elements^1^, and epigenetic modifications such as chromatin remodelling through DNA methylation and post-translational histone modification^2,3^. The second aspect is the higher-order genome organization^4^, the 3D architecture of the nucleus in which two meters of DNA^5^ is fitted into a 6–10 μm diameter sphere^6^. This structure, linking distal regulatory motifs such as promoters and enhancers, functionally influences cellular processes including protein biosynthesis^7^. The 3D genomic organization could be captured by various methods based on chromosome confirmation capture (3C), however these experimental methods are expensive. They are specifically tailored to detect either local or global spatial interactions at unprecedented resolution, however, they are affected by noise introducing false positive interactions, or by unavoidable systemic biases. 3C classical methods are not genome-wide, instead they are limited from ten to several hundred kilobases. Chromosome conformation capture-on-chip “4C” methods are genome-wide, whereas chromosome conformation capture carbon copy “5C” can measure many anchored profiles in parallel, therefore, they analyse the chromatin interactions for large numbers of genomic loci efficiently^8^. The Hi-C method generates an all-to-all interaction map with a resolution depending on the sequencing depth. Some computational methods were proposed to improve the resolution of Hi-C heatmaps^9^. Chromatin conformation capture sequencing Hi-C considered the first unbiased genome-wide method, and it captures the interactions mediated by several proteins. Finally, chromatin interactions analysis by paired-end tag (ChIA-PET) method integrates the 3C method with chromatin immune-precipitation to detect interactions mediated by a specific protein. The association between one-dimensional and higher order structure has yet to be well established and requires further investigation and analysis. Identification of strategies for the prediction of 3D architecture may allow identification of long-range non-coding regulatory elements such as promoters and enhancers, located thousands or millions of base pairs away from their target gene^10^. Attempting to predict genome-wide interactions is a challenging task given the number of possible pairwise interactions as ${({genome\; length}/{resolution})}^{2}$, i.e. 10^8^ pairs of genomic segments for the human genome with 100 kb resolution. Less than 0.01% of these pairs are true physical interactions as confirmed by an experimental method such as *in situ* Hi-C^11,12^, or ChIA-PET^13–15^. The number of possible pairs may be reduced by forming pairs based on the distance between interacting genomic segments (anchors), or following interactions established Topologically Associating Domains (TADs). However, this is still insufficient to provide accurate statistical predictions due to the large number of possible formed pairs.

Another complicating factor is the diversity of biophysical characteristics of chromatin interactions. Previous studies have proposed interesting solutions to the problem of functional link between epigenomics and chromosomal organization. First, Di Pierro *et al*. performed *de novo* computational prediction of chromosomes structures and compartmentalization using epigenetic profiles as patterns that encode multiscale spatial architecture of Human genome at the resolution reaching 50 kb^16^. Other studies focused on 3D interactions between specific regulatory elements, such as enhancers and promoters^17–22^. Recent study of Nikumbh and Pfeifer^20^ extends this approach by analyzing structural interactions mediated by intervening chromatin that elucidates the role of short tandem repeats in sequence-based prediction of long-range chromatin interactions. Finally, some approaches find possible interactions within all combinations between the genomic segments that share the same epigenomic profiles^23^. Di Pierro *et al*.^24^ proposed transferable model of chromosome architecture that exploits novel idea of different classes of the physico-chemical characteristic of chromatin fiber. Each type of chromatin state (controlled epigenetically) is linked with the unique repertoire of its own biochemical interactions, and it is changed during cell differentiation.

In this contribution, we develop *3DEpiLoop*: a tool for the prediction of 3D genome-wide chromatin interactions using 1D genomic structure (mainly epigenomics and transcription factor assays). The resolution of the predictions obtained by 3DEpiLoop is very high (1 kb segments). The predictions are genome-wide and not restricted to the genomic segments containing regulatory elements (Enhancer, Promoters), or the motifs of the binding protein. In our approach, we build a universal statistical model which can be adapted to any type of interactions. Our supervised learning predictor targets each cell type separately to reduce the bias towards the most common interactions across different cell types. Moreover, it uses the peaks of the mediating protein to capture all possible interacting segments whether they contain regulatory elements or not. We aim to identify the important epigenomic modifications and transcription factors, which uniquely characterize the interacting genomic segments. Further, we perform complex analysis of the epigenomic and transcription profiles of the interacting anchors at different genomic scales: from genomic domains to whole genome. We separately optimise and validate this tool on a range of different interactions types such as CTCF ChIA-PET, RNAP II ChIA-PET, *in situ* Hi-C loops, and *in situ* Hi-C heatmaps, and identify common predictive features.

## Results

### 3DEpiLoop identifies efficiently the interacting genomic segments using the binding profiles of the mediating proteins

3DEpiLoop uses the binding profile of the mediating protein to determine the initial set of genomic segments, which contain the interacting anchors. The MACS peak calling method is applied to obtain the peaks from ChIP-seq data^25^. MACS identifies the peaks from ChIP-seq data with the high resolution by empirically modelling the shift size of ChIP-seq reads and using dynamic Poisson distribution to minimize the local biases in the genome for better prediction. We found that the identification of peaks dramatically reduces the number of segments being analysed while maintaining most of the interactions mediated by the corresponding protein. Furthermore, we determined the subset of epigenomic features enriched in the anchors of different loops types (CTCF ChIA-PET, RNAP II ChIA-PET, and Hi-C loops). These features were used to rank the genomic segments to eliminate likely physically non-interacting segments. In the case of Hi-C heatmaps, we do not apply any filtration because such frequency data (from next generation sequencing) represents spatial distance, therefore the majority of the segments within the same TAD are reported as proximal in addition to the diversity of the mediating proteins in these interactions. Using CTCF peaks and the above-mentioned filtration procedure we identify 32,378 segments at 1 kb resolution covering 29,159 of 29,295 (99.5%) of experimentally determined CTCF-mediated ChIA-PET interactions for the GM12878 cell line^13^. The same procedure captured 87% of K562 and 90% of HeLa CTCF ChIA-PET interactions^13^, and 90% of MCF7 CTCF-mediated interactions (GSM970215). In the case of RNAP II ChIA-PET interactions and corresponding RNAP II ChIP-seq peaks the filtered segments cover 93% of GM12878 interactions, 83% of K562 interactions and 92% of HeLa Interactions (Supplementary Methods sections 2.3.2, 4.2 and Supplementary Table 2).

**Figure 1:**
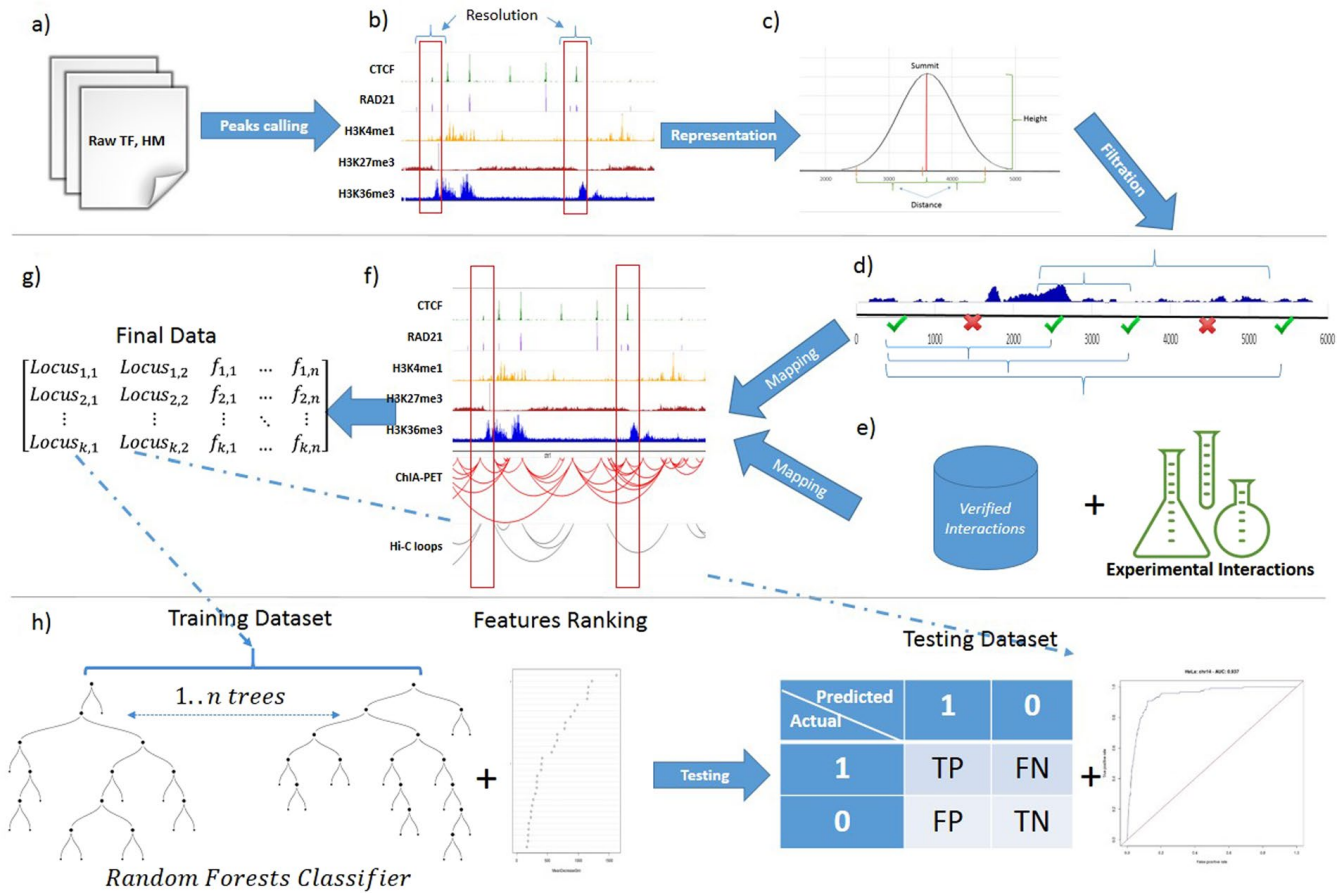
3DEpiLoop Supervised Learning Pipeline. (**a**) Transcription factors and histone modification experimental data is provided either as bam or bed input files. (**b**) MACS2 peaks calling method is applied to obtain the protein binding and histone modifications peaks. (**c**) Each locus is represented by the peak height and the distance between the peak summit and the centre of genomic segment. (**d**) A filtration process is applied to eliminate the non-interacting segments, then all possible pairs are formed from the remaining segments within the same genomic domain. (**e**) The experimental interactions, or any other set of verified or curated interactions, are mapped on the genome and considered as true interactions for training. (**f**) 80% of the pairs are randomly selected as the training dataset, while the rest (20%) as the testing dataset. (**g**) The training dataset is used to train a random forests classifier (or any other classifier) and to determine the ranking of important features for statistical prediction. (**h**) The testing dataset is used to evaluate the performance of the predictor by comparing results with the true interactions confirmed by experiments.

The second step in the interaction prediction procedure is to apply a supervised learning method to predict which pairs of the candidate genomic loci are truly interacting. 3DEpiLoop utilizes a Random Forests algorithm to predict the interactions within the same genomic domain (TAD), where the majority of the intra-chomosomal interactions are observed by experimental methods^11,13,23,26^. We also tried several classifiers such as AdaBoost classification trees, neural networks, Support Vector Machines SVM, and Stochastic Gradient Boosting in order to find the best classifier for such problem. Figure 1 illustrates the workflow implemented in our study, starting from data preparation and concluding with an *in silico* predictor performance evaluation procedure. See the Methods section for more details regarding the whole workflow and the classifiers used in this study.

### Validation with ChIA-PET Interactions

The 3DEpiLoop supervised learning pipeline was assessed in detection of the physical interactions acquired from Chromatin Interaction Analysis by Paired-End Tag Sequencing (ChIA-PET) experiments. Our focus in this study was on two types of ChIA-PET interactions: 1- CTCF-mediated looping, which is typically related to the long-range architectural interactions defining the three-dimensional higher-order genomic organization of the genome. 2- RNAP II mediated ones which are shorter, functionally linking the enhancers and promoters involved in the DNA transcription process^27^.

**Figure 2:**
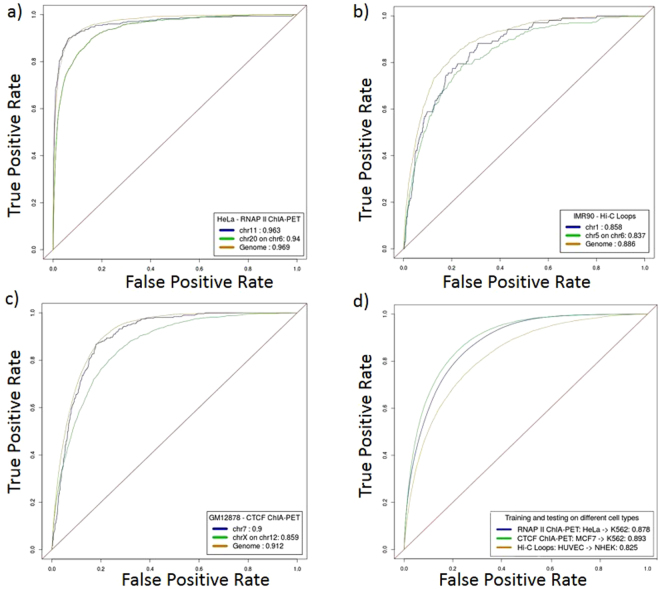
Performance Evaluation using ROCs Curve. (**a**) ROC Curves for RNAP II ChIA-PET interactions predictors obtained from HeLa cell line in genomics and chromosome scales. Blue when training and testing on chromosome 11. Green when training on chromosome 20 and testing on chromosome 6. Orange when training and testing on the whole genome. (**b**) ROC Curves for Hi-C Loops predictors obtained from IMR90 cell line in genomics and chromosome scales. Blue when training and testing on chromosome 1. Green when training on chromosome 5 and testing on chromosome 6. Orange when training and testing on the whole genome. (**c**) ROC Curves for CTCF ChIA-PET interactions predictors obtained from GM12878 cell line in genomics and chromosome scales. Blue when training and testing on chromosome 7. Green when training on chromosome X and testing on chromosome 12. Orange when training and testing on the whole genome. (**d**) ROC Curves for genome-wide predictors when training and testing on different cell types. Blue when training RNAP II ChIA-PET Interactions predictors on HeLa and testing on K562. Green when training CTCF ChIA-PET Interactions predictors on MCF7 and testing on K562. Orange when training Hi-C loops predictors on HUVEC and testing on NHEK.

CTCF ChIA-PET interactions identified by improved long reads ChIA-PET^11^ were used as true interactions for training in GM12878, K562, and HeLa cell lines, whereas MCF7 CTCF ChIA-PET interactions were extracted from GSM970215. RNAP II ChIA-PET interactions for GM12878, and HeLa were obtained from^13^, K562 from GSM970213, and mouse CH12.LX^28^. The average accuracy, sensitivity, specificity, and area under the ROC curve for all these tests are greater than 0.82, 0.78, 0.82, and 0.89 respectively. Figure 2 illustrates ROC Curves obtained from representative predictors. The results confirm the success of 3DEpiLoop pipeline in recovering physical interactions (Supplementary Methods sections 2, 4 and Supplementary Tables 3 and 5). The high quality of 3DEpiLoop, when training and testing across all possible combinatorial possibilities at several genomic scales (genomes, chromosomes, genomic domains, chromatin loops) confirms that the anchors (i.e. interacting segments) from ChIA-PET interactions have very similar epigenomic and transcription factor profiles across different cell types, chromosomes, and topologically associating domains. We noticed that transcription factors play a major role in CTCF ChIA-PET interaction determination; they appear at the top of the features of importance rankings according to the Random Forests machine learning classifier. We found that RNAP II interactions have different epigenetics preferences as compared with CTCF interactions. We identified the higher importance of histone modifications as compared with the importance of transcription factors, with a single exception for RNAP II protein binding profile, which is expected as it is the mediating protein. We believe that the better normalization could be applied to further improve the results, especially when testing predictors between differently prepared experimental datasets. See The Importance Rankings of Features used in interactions identification section for further details.

### Validation with Physical *in situ* Hi-C Loops

We compared the predictions of the 3DEpiLoop algorithm with physical interactions from *in situ* Hi-C that are mediated by any architectural protein. The chromatin loops were identified in GM12878, K562, HeLa, IMR90, HMEC, NHEK, HUVEC, and CH12.LX cell lines as physical interactions using the contact maps^11^. In brief, they searched for contacts in the Hi-C heatmaps that show closer proximity to each other, in comparison with their surrounding genomic segments. CTCF ChIA-PET predicted interactions obtained by 3DEpiLoop were highly concordant with *in situ* Hi-C loops for the cell lines GM12878, K562, and HeLa. Further, the 3DEpiLoop algorithm was successfully trained and tested on Hi-C identified loops. The average accuracy, sensitivity, specificity, and area under the ROC curve for all these tests are 0.79, 0.67, 0.79, and 0.81 respectively. Figure 2 illustrate ROC Curves obtained from representative supervised learning predictors (Supplementary Methods section 3 and Supplementary Table 4). The *in situ* Hi-C loops reported in the experimental study were less abundant than ChIA-PET reported interactions. Therefore, the efficiency of trained statistical models is affected by the number of true interactions in the training dataset. However, in general the predictor maintains its overall stability in the prediction even with a smaller number of training examples. Moreover, 3DEpiLoop uses CTCF ChIP-seq peaks to filter out genomic segments that are tested to form the pairs. This approach is not effective in the case of *in situ* Hi-C loops, because they can be mediated by the variety of different proteins, and not only CTCF, or RNAP II. Approximately 71% of the predictions were reported with the experimental CTCF ChIA-PET interactions. We also compared the predictions obtained by 3DEpiLoop when training on CTCF ChIA-PET physical interactions. We found that CTCF ChIA-PET trained predictor could predict ~75% of Hi-C loops for GM12878, K562, and HeLa cell lines. See Supplementary Methods section 3.4 and Supplementary Figure 17.

### Comparison with EpiTensor

The primary issue with existing computational methods for the prediction of interactions is the high false positive rate. This high false positive rate makes it difficult to interpret and apply predicted interactions. In contrast to our method, EpiTensor^23^ interactions are obtained by an unsupervised learning algorithm which uses tensor decomposition to find the genomic loci with similar epigenomic patterns across cell types, later assessing the strength of the interactions using ChIP-seq peak height at both analysed anchors. We compared EpiTensor interactions with CTCF/RNAP II ChIA-PET interactions obtained by the 3DEpiLoop predictor applied on the K562 cell line^13^, GSM970213, and with *in situ* Hi-C loops for the IMR90 cell line^11^. The results are reported in the Table 1. While both approaches obtained a lot of false positives, 3DEpiLoop captured far more experimental interactions, at least three times more than Epitensor. Additionally, fewer false positives were observed when dealing with CTCF ChIA-PET, and *in situ* Hi-C loops, though the false positive rate was higher with RNAP II ChIA-PET interactions. (Supplementary Methods sections 2.4, 3.3, and 4.5). Similar studies tried to predict functional interactions mediated by RNAP II and linking enhancers and promoters^21,22^. Both approaches are limited to the enhancers and promoters interactions, while 3DEpiLoop can predict larger set of interactions, genome-wide without any limitations. IM-PET^21^ targeted 2,219 interactions of 652,800 candidates. RIPPLE^22^ targeted less than 1000 interactions, then the authors expanded it to cover genome-wide enhancer-promoters interactions. The genome-wide predictions were compared with Hi-C contacts. We believe that Hi-C contacts and scores without loop calling post-processing do not provide a strong evidence for the physical interactions, and they are highly influenced by the genomic distance. 3DEpiLoop targets ~25000 physical interactions without any limitation, approximately 12, and 25 times what IM-PET and RIPPLE cover respectively.

**Table 1:** Comparison with EpiTensor: Experimental Results column represents the interactions reported by experimental methods, where the anchors in the analyzed TADs, and contain the mediating protein signal.

Cell line Interactions	Experimental Results	Epitensor predictions	Epitensor Verified	3DEpiLoop predictions	3DEpiLoop Verified
K562: CTCF ChIA-PET	30,978	209,086	1,331 (4%)	93,027	18,945 (61%)
K562: RNAP II ChIA-PET	26,371	209,086	5,006 (18%)	1,044,929	15,226 (57%)
IMR90: *In situ* Hi-C loops	4,343	105,799	746 (17%)	92,474	3,359 (77%)

(EpiTensor/3DEpiLoop) Predictions column represents the total count of all prediction for both computational methods. (EpiTensor/3DEpiLoop) Verified column represents the count and percentage of the prediction which are verified experimentally.

### Validation with *in situ* Hi-C Heatmaps

**Figure 3:**
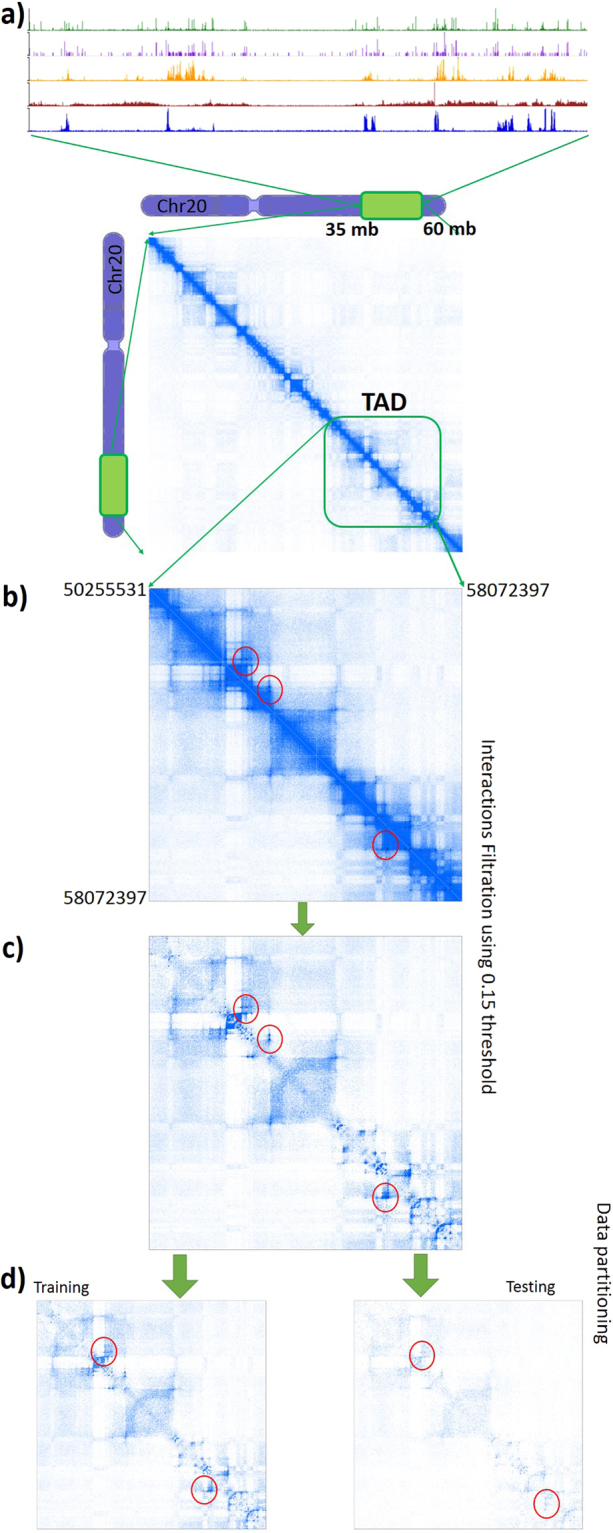
Heatmaps transformation for analysis. (**a**) *in situ* Hi-C heatmap representing the specific part of Chr20 in GM12878 cell line (35 mb − 60 mb) including the signals of CTCF, RAD21, H3K4me1, H3K27me3, and H3K36me3. (**b**) The heatmap of single TAD: Chr20:50255531–58072397. The red circles surround physical interactions. (**c**) Further, we illustrate the filtered heatmap using 0.15 as threshold, i.e. all interactions with genomic distance based score less than 0.15 are considered as true interactions. (**d**) The training heatmap (80% of the original) is on the left and it was used to train the predictor, while the testing heatmap (20%) is on the right. There is no common samples between training and testing datasets.

*In situ* Hi-C interactions heatmap reflects spatial proximity between genomic segments. It is difficult to identify physical interactions using Hi-C interaction strength. The score of a given contact is significantly influenced by the genomic distance between anchors^29^. As such, most loci, especially those located within the same TAD, interact to some degree. Our approach predicts physical interactions with the highest possible resolution (5 kb), whereas the previous study^16^ analysed the interactions within the genomics compartments in 50 kb resolution using neural network. To account for this we define the importance, or the interaction strength, as the score relative to the genomic distance between the anchors. In this way, we distinguish between the true interactions (more likely to interact because of a physical contact), and false interactions (no evidence for any contact, or very low score) using an optimal threshold. The threshold (0.15) was determined in a way that preserves high coverage of the physical interaction (i.e. ChIA-PET^13^ and *in situ* Hi-C loops^11^) within true interaction sets and takes the predictor performance into the consideration (Supplementary Methods section 5 and Supplementary Table 6). In the GM12878 cell line, the *in situ* Hi-C interactions stronger than the chosen threshold covered 14167/14187 (99.8%) of Hi-C loops, and 53519/55705 (96%) of CTCF ChIA-PET interactions experimentally reported^11,13^. Figure 3 illustrates the *in situ* Hi-C heatmap transformation procedure. Training and prediction were done separately on 2232 TADs^13^. We performed tests on GM12878, K562, IMR90, HUVEC, HMEC, and NHEK cell lines using *in situ* Hi-C heatmaps (5 kb resolution) from the study^11^ with the same set of epigenomic features. While the diversity of the proteins mediating Hi-C interactions, in addition to the noisy experimental data, affected predictive performance, 3DEpiLoop could recover most of the physical loops from both Hi-C and ChIA-PET. The average accuracy, sensitivity, specificity, and area under the ROC curve for all these tests are greater than 0.64, 0.51, 0.73, and 0.63 respectively. These measurements were calculated according to the *in situ* Hi-C proximate interactions after the filtration. We calculated the coverage of the physical interactions in the obtained predictions in order to assess the quality of the predicted interactions. We found that the obtained predictions covered 70% of *in situ* Hi-C loops and 80% of CTCF ChIA-PET interactions.

### Population Hi-C Heatmaps Analysis

**Figure 4:**
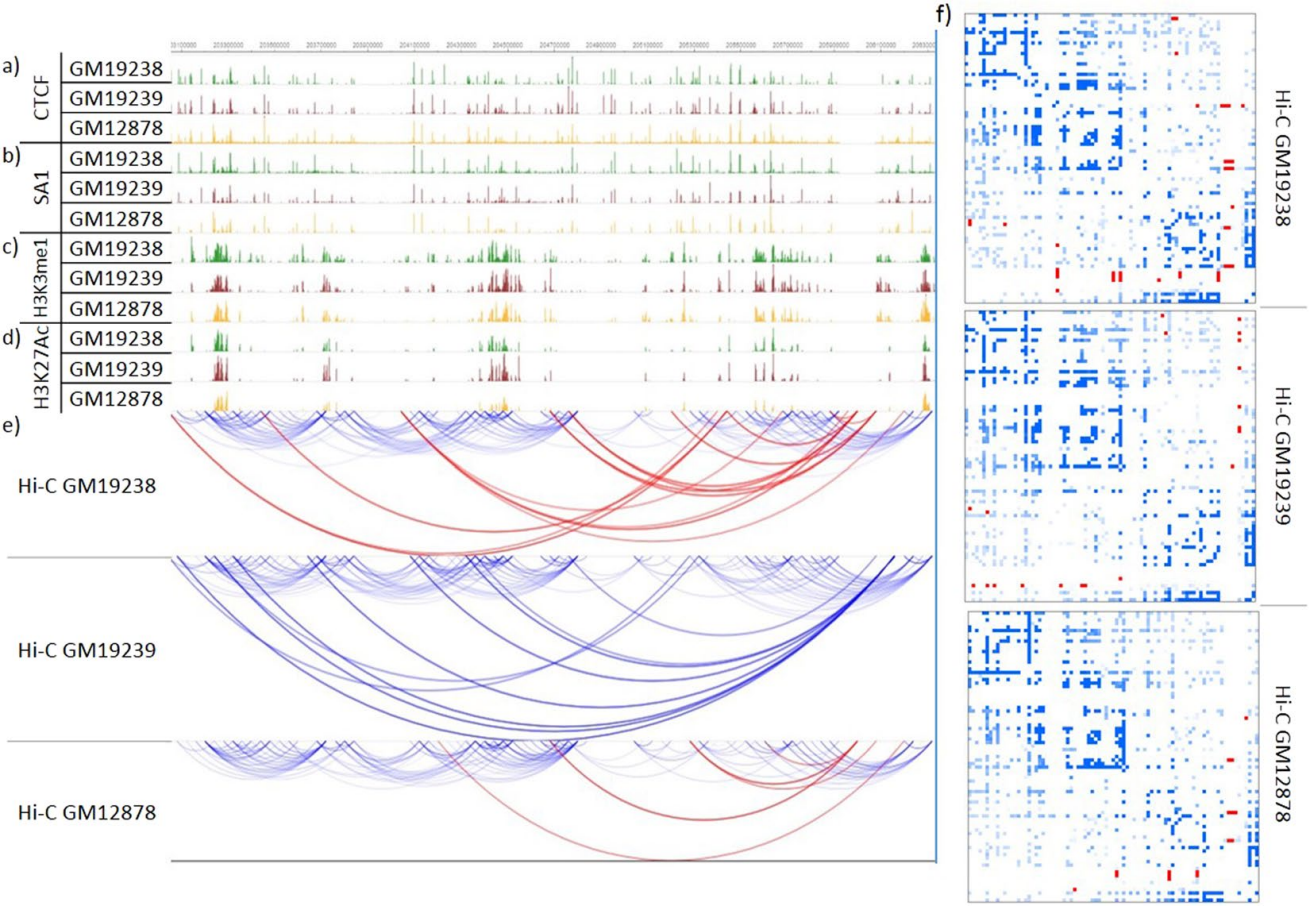
Epigenomics, Transcription factors, and Hi-C heatmaps (Chr1:203000000–206000000) in three individuals of GM12878 cell type. (**a**) Three tracks represent CTCF signals in three individuals. (**b**) Three tracks represent SA1 signals. (**c**) Three tracks represent the histone modification H3K4me1. (**d**) Three Tracks represent H3K27Ac Histone Modifications. (**e**) Hi-C approximate interactions predicted by 3DEpiLoop using 40 kb resolution. Tracks in green are for the cell type GM19238, red for GM19239, and yellow for GM12878. Arcs in blue are correct predictions, while incorrect ones are in red. (**f**) Hi-C heatmaps for the same region of the cell lines (40 kb resolution) after the filtration. Blue pixels are the correct predictions while the red ones are the wrong.

In the next steps of our analysis, we assessed 3DEpiLoop on data from lymphoblastoid cell lines of seven individuals (HG00731, HG00732, HG00513, HG00514, and the family trio GM19238, GM19239, and GM19240) from 1000 Genome Project. Heatmaps for these cell types were at 40 kb resolution (private communication from Bing Ren laboratory). 3DEpiLoop was trained on each individual and tested on all other individuals as well as GM12878. Using 3DEpiLoop interactions were recovered from the testing samples with an average accuracy of 0.87, sensitivity of 0.87, specificity of 0.86 and precision of 0.84. These tests confirm that the epigenomic and transcription factors profiles are conserved between individuals when comparing the same cell type. In general, individuals, share very similar epigenomics and transcription factors profiles and Hi-C heatmaps. Figure 4 illustrates the epigenomics and transcription factors, in addition to the predicted Hi-C heatmaps (strong interactions for visualization) of the cell lines GM19238, GM19239, and GM12878. We believe that lowering the resolution of the heatmaps makes it easier for the statistical predictor to distinguish between true interactions and non-interactions (Supplementary Methods section 5.4, and Supplementary Table 6).

### Transcription factors and Histone modification profiles are unique to the interaction type

**Figure 5:**
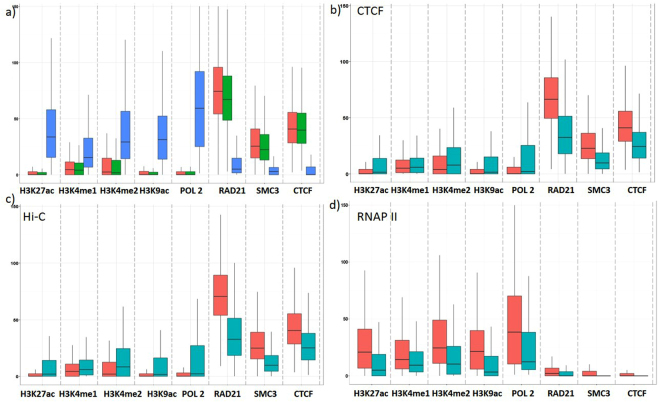
Features distribution in GM12878 cell type. The X-axis represents different histone modifications and transcription factor assays, while the Y-axis represents the sum of the assay peak’s height at both anchors. (**a**) the distribution of the important transcription factors and histone modifications among Hi-C loops (Red), CTCF (Green) and RNAP II ChIA-PET (Blue) interaction anchors; (**b**) the distribution of the features in the anchors of predicted CTCF ChIA-PET interactions and the non-interacting genomic segments. Red boxes represent the interactions, while green represent the non-interacting ones; (**c**) the distribution of the features in the anchors of predicted Hi-C loops and the non-interacting genomic segments. Red boxes represent the interactions, while green represent the non-interacting ones; (**d**) the distribution of the features in the anchors of predicted RNAP II ChIA-PET interactions and non-interacting genomic segments. Red boxes represent the interactions, while the green represent the non-interacting ones.

We analyzed CTCF ChIA-PET, RNAP II ChIA-PET and Hi-C interactions to assess whether anchors of each interaction type have preference for specific epigenetic features. We discovered that Hi-C loops and CTCF ChIA-PET interactions share similar patterns for epigenomic and transcription factors, with enrichment of transcription factor peaks at anchors, but depletion at the site of histone modification signals. In contrast, RNAP II ChIA-PET anchors were enriched with histone modifications and depleted in transcription factors signals (with the exception of RNAP II binding). In addition, we evaluated the predictions obtained by 3DEpiLoop to check whether the predictions share the same feature patterns. We compared the distribution of the features between the interacting genomic segments (signal) and the non-interacting ones (background). Our results confirm similar conclusions for Hi-C loops, CTCF and RNAP II ChIA-PET interactions. Figure 5 illustrates the distribution and the preferences according to each interaction type in GM12878 cell line (Supplementary Methods section 6).

### The Importance Rankings of Features used in interactions identification

The importance of different epigenomics and transcription factor preferences at the interaction anchors can be evaluated by the Random Forest features ranking^30^. For further verification, we applied chi-square test of independence in addition to Monte Carlo Feature selection Method^31^ to assess the importance of the features. The top significant features were highly consistent according to all feature selection methods. The statistical significance “p-value” calculated by chi-square test was less than 5.6 * 10–12, 1 * 10–118 and 1.9 * 10–2 for the top 35 significant features reported for CTCF ChIA-PET, RNAPII ChIA-PET, and Hi-C loops respectively. Histone modifications were ranked as more important than transcription factors in the case of RNAP II ChIA-PET interactions, whereas the opposite trend was observed for *in situ* Hi-C physical loops and CTCF ChIA-PET interactions. The genomic distance between the interacting segments together with the segment order in each TAD were ranked within the top features in most cases. The binding profile of the mediating protein such as 11-Zinc finger protein (CTCF) and RNA polymerase II (RNAP II) were among the highest ranked features, when predicting their corresponding interactions (CTCF ChIA-PET, RNAP II ChIA-PET). Double-strand-break repair protein (RAD21), and structural maintenance of chromosome 3 protein (SMC3) binding enrichment in addition to the histone H2A encoded by H2AFZ gene (H2AZ), monomethylation of histone H3 at lysine 4 (H3K4me1), dimethylation of histone H3 at lysine 4 (H3K4me2), acetylation of histone H3 at lysine 27 (H3K27ac), and finally the trimethylation of H3 lysine 36 (H3K36me3) signal depletion were important epigenomic/transcription factor signatures in the case of Hi-C physical loops prediction. CTCF ChIA-PET interactions were strongly linked with the transcription factors CTCF, RAD21, SMC3, and Zinc finger protein 143 (ZNF143). In contrast, the histone modification enrichment was the most important indicator of RNAP II ChIA-PET interactions, especially H3K27ac, H3K4me2, trimethylation of histone H3 at lysine 4 (H3K4me3), and acetylation of histone H3 at lysine 9 (H3K9ac). The same features were reported in previous study^22^. Those complex relations identified in human cells were also observed for the mouse cell line. Finally, the heatmaps prediction is characterized by a mixed set of preferences, where both transcription factors and histone modifications were important. CTCF, Cohesin subunit (SA1), H3k4me1, H3K27ac, H3K4me3, and H3K36me3 are the most important features according to the predictors for the lymphoblastoid cell lines (different individuals) when predicting Hi-C heatmaps. The similar histone modifications were reported also in this study^16^. H3K4me1, H2AZ, H3K4me2, CTCF, H3K27ac, H3K79me2, and H3K9AC were the most important features according to *in situ* Hi-C heatmaps predictors for the GM12878, K562, HUVEC, HMEC, and NHEK cell lines (Supplementary Methods sections 2, 3 and 4).

### Statistical Models Specificity

Some physical interactions especially the functional ones between enhancers and promoters are cell type specific^21^ while the others are conserved between different cell types^23,11^. This idea was employed by the EpiTensor algorithm^23^, where the data from five different cell lines were combined and shown to have conserved epigenomic patterns that are the hallmarks of physical interactions. Our study trains a supervised predictor on single cell line separately by using only its own data in order to detect all possible interactions, whether they are common and shared between cell types, or they are unique to this specific cell type. Such specificity of the input data used for training and constructing a single statistical model for each interaction type and cell line allowed us to build a very sensitive statistical model, reducing the bias toward the shared interactions between different cell types, or to the most prominent interaction types. A similar idea was employed to predict enhancers-promoters interactions^22^, while 3DEpiLoop targets genome-wide interactions which is more complicated problem. For example, 29,295 and 30,978 CTCF interactions for GM12878 and K562 cell types respectively were reported in the TADs used in this study. Only 12,033 (41%, 38%) pairs among these interactions are common and shared between both cell types. Our approach predicted 99,912, and 85,574 CTCF ChIA-PET pairs in GM12878 and K562. 24,676 GM12878 interactions and 18,731 from K562 were covered by our predictions, and only 9237 pairs were shared between them, while the rest were interactions unique to those cell types.

## Discussion

Our study confirms the feasibility of predicting the high resolution (1 kb) three-dimensional chromatin physical interactions using 1D epigenomic and transcription factor binding profiles. Up to the best of our knowledge, 3DEpiLoop is the second attempt (after EpiTensor) that predicts genome-wide 3D interactions without any limitation or restriction related to the existence of the regulatory elements or the mediating protein motifs in the interacting anchors. Moreover, it shows that complex epigenomic and transcription factor signatures are conserved across different genomic domains, chromosomes, cell types and within the population of individuals. However, these signatures vary according to the interaction types. We found that RNAP II ChiA-PET Interaction anchors are enriched by epigenomics signals, while CTCF ChIA-PET and Hi-C Loops are enriched by transcription factors. Using the mediating protein signal to prepare the initial genomic loci for the analysis reduces the size of the dataset dramatically whilst keeping the majority of the interacting loci. Scoring the segments using all important features could separate the interacting from the non-interacting segments efficiently. We believe the better filtration we do in the segments level before forming the pairs, the more accurate prediction we get. Some applied tests confirmed the possibility to predict RNAP II ChIA-PET interactions in mouse cell types using predictors trained on human cell line and vice versa. We believe it is also true for other interaction types, but we couldn’t apply the verification tests as the mouse interactions were not readily available. These Human-Mouse tests obtained the worst performance evaluation results which could be related to the difference in the quantity of the interactions between the human and mouse cell lines. Obtaining a lot of false positives is still an issue, but this is a usual problem related to the nature of the data (unbalanced data: very few interactions comparing with too many non-interactions pairs). Many of the false positives obtained by 3DEpiLoop were also reported in EpiTensor predictions, they might be verified by other experimental methods. We found that 22% (16367 of 74862) of the false positives predicted by 3DEpiLoop when trained on K562 cell line and applied on GM12878 cell line were reported experimentally in a different dataset. This dataset includes only the interactions between anchors that don’t have CTCF motifs. The success of our approach for detecting several physical interaction types as compared with previous approaches, in addition to its usability to predict other cell type interactions precisely without any prior knowledge about the cell type, make our approach a valuable tool to determine chromatin interactions. In addition to the interactions conserved among different cell types, our approach is able to predict the subset of unique interactions for each cell type. The high predictor specificity is gained by including the epigenomic and transcription factor data for this specific cell type. Hi-C interactions prediction is still a challenging problem due to the diversity of the mediating proteins in addition to the abundance of proximity-based interactions. When trying to separate the physical interactions from the spatial proximity illustrated by the heatmaps, the signal of the epigenomics and transcription factor patterns within the physical contact anchors is more likely to be strong and differentiable compared with proximity interactions in Hi-C experiments. Our approach to define the important Hi-C interactions relative to the genomic score helps to reduce the influence of the genomic distance in such supervised learning for those interactions. This study assessed the influence of the genomic distance by training two different models using the same features, the first model included the genomic distance while the second excluded it. The predictors trained without the distance could predict the interaction with slightly worse performance comparing with the ones including the genomic distance. Choosing the optimal threshold to separate the heatmap interactions in the training phase is the critical issue. Choosing threshold θ results three classes of interactions: important interactions when the *genomic distance based score* ≤ θ, not interactions when the *genomic distance based score* ≥ (1- θ), and *no-class* when θ < *genomic distance based score* < (1- θ). The simplest is to choose θ = 0.5 to cancel the *no-class* category. We found that using 0.15 and excluding the *no-class* interactions from the training dataset allow us to cover the majority of the physical interactions without sacrificing the performance of the predictor. In general, *in situ* Hi-C predictors obtained the worst results when predicting the high resolution heatmaps but they covered about 75% of the physical interactions.

We believe that such predictors or approaches should use both epigenomics and transcription factors in order to capture several interaction types. Using transcription factors was essential to recover CTCF ChIA-PET, and *in situ* Hi-C loops. This gave the advantage over EpiTesnor, when dealing with these interactions. There is still room for improvement, we believe that the more features uniquely representing the pairs the better performance one could get (such as CTCF motif orientation features, which improve CTCF interactions prediction). Performing better normalization of the input data and experimental interactions collected from different laboratories will improve the performance of the suggested approach especially when applying human cell lines trained predictor on mouse cell lines and vice versa. Our algorithm use training dataset with the variety of different features and it can be easily extended to include various types of sequence-based information (such as short tandem repeats^20^). We are now working on the sequence-based predictor, the initial results are promising. The 3DEpiLoop algorithm allows for adding novel physical interactions mediated by specific proteins into the statistical model, actually classifying possible chromatin binding motifs (both in terms of sequence specificity, physical, or chemical local properties) could improve the accuracy of the method^24^.

## Methods

### Data Preparation

The experimental datasets used in this study were obtained from multiple online resources. The epigenomics and transcription factors peaks were called using the MACS2 package^25^ utilizing previously reported parameters^32^. The human genome was divided into segments with predefined resolution according to the validation data (1 kb for physical interactions prediction, 5 kb and 40 kb for Hi-C heatmaps). The called peaks from all assays were assigned to the genome, and each locus was represented by the height of the peak, together with the distance between the centre of the locus and the summit of the peak (two features for each assay). The conflict between the assay peaks in the same genomic segment was resolved using either the maximum height when predicting high resolution physical interactions <5 kb, or the sum of the peaks’ heights when predicting low resolution heatmaps >40 kb. We analyzed eight human cell lines (GM12878, K562, HMEC, HUVEC, IMR90, HeLa, NHEK, and MCF7), seven lymphoblastoid cell lines (HG00731, HG00732, HG00513, HG00514, GM19238, GM19239, and GM19240) from the 1000 Genomes Project^33^. And the CH12.LX mouse cell line. We used epigenomic assays characterizing genomic segments by CTCF, RNAP II, RAD12, ZNF143, SMC and SA1 transcription factor binding profiles, and a variety of histone modifications: H2AFZ, H3K27ac, H3K27me3, H3K36me3, H3K4me1, H3K4me2, H3K4me3, H3K79me2, H3K9ac, H3K9me1, H3K9me3, and H4K20me1 (Supplementary Table 1).

### Segments Filtration

To reduce combinatorial complexity, we first applied a filtration pipeline to eliminate likely non-interacting loci before predicting physical interactions. We identified the subset of the features, which are enriched in the interacting segments as compared to other loci within the available distribution of epigenomic and transcription factor binding assays. The segments enriched by CTCF, SA1, RAD21, SMC3, and ZNF143 transcription factor signal are more likely to interact in the case of Hi-C loops, and CTCF ChIA-PET interactions. RNAP II physical interaction anchors are enriched by H3K4me1, H3K4me3, and H3K27Ac histone modifications. In this study, we define the key-features set to refer to these most important assays. This pipeline consisted of the following steps: First, loci with peaks for the mediating protein (CTCF for CTCF ChIA-PET and Hi-C Loops. RNAP II for RNAP II ChIA-PET interactions) were retained. Secondly, each segment was scored according the key features, that is the subset of the features from epigenomic and TF binding assays which were enriched in the interacting segments. The segments were filtered using an optimal threshold defined according to the availability of key-features and analysis of the distribution of interacting and non-interacting segments.

### Candidate Pairs

The final candidate pairs are selected from the filtered segments by considering all possible combinations within each genomic domains, as the majority of chromatin interactions occur within the same TAD^34,35^. The interacting pairs (few pairs from all possible combination within TADs that match the experimental interactions) together with the non-interacting pairs (a lot of pairs don’t match any experimental interactions) formed the final data for training and testing. Each pair is presented by the maximum and minimum values of the right and left anchor features. The percentage of the interacting pairs to the non-interacting ones ranges from 0.01 to 0.1 according to the interaction types and the availability of the experimental interactions. See the supplementary Table 1 for detailed information. We used chromatin contact domains (CCDs) genomic coordinates from the GM12878 cell line^13^ to maintain the position of topologically associating domains across cell lines. The localization of three-dimensional domains is typically conserved among different cell types and even mammals^36^.

### Training and Testing Datasets

Training and testing datasets were prepared at multiple levels: genomic domains, chromosome, and whole genome. The statistical models were constructed and evaluated separately at all levels for all possible combinations. In all applied tests the classifier has no prior information about the testing dataset. Both training and testing sets are constructed by random selection of the samples from the same cell line, or by training on a cell line and testing on another one. 3DEpiLoop uses the Random Forest classifier, a machine learning approach that uses multiple decision trees and voting for the final predictions^37,38^. We also applied different classifiers such as AdaBoost classification trees, neural networks, Support Vector Machines SVM, and Stochastic Gradient Boosting. All of these classifiers obtained very similar results with an exception for neural network (lower accuracy). The performance of the predictors was described by the accuracy, sensitivity (quality of the predictor when detecting the real interactions), specificity (quality of the predictor to detect the non-interacting pairs) and the area under the ROC curve (refers to the probability that the predictor will rank randomly chosen interacting pairs higher than randomly chosen non-interacting pairs)^39^. These measurements are calculated according to the confusion matrix elements (contingency table)^40^: We considered a prediction as True Positives when the left anchors of both pairs (prediction and validation) were overlapping, and the same for the right anchors. The prediction probability obtained by the Random Forest classifier was used to calculate the area under the ROC curve.

## Electronic supplementary material

Supplementary Materials

Supplementary Table 1

Supplementary Table 2

Supplementary Table 3

Supplementary Table 4

Supplementary Table 5

Supplementary Table 6
