# Supplementary material for: Three-dimensional Epigenome Statistical Model: Genome-wide Chromatin Looping Prediction
Source: Sci Rep. 2018 Mar 26;8:5217. doi: 10.1038/s41598-018-23276-8 (PMC5979957; doi:10.1038/s41598-018-23276-8)
Supplement: Supplementary file 1 — Supplementary Materials [file 41598_2018_23276_MOESM1_ESM.docx]

Three-dimensional Epigenome Statistical Model: Genome-wide Chromatin Looping Prediction

Ziad Al Bkhetan^1,3^ and Dariusz Plewczynski^1,2,*^

^1^ Centre of New Technologies, University of Warsaw, Warsaw, Poland

^2^ Faculty of Mathematics and Information Science, Warsaw University of Technology, Warsaw, Poland

^3^ Biology Department, University of Warsaw, Warsaw, Poland.

^*^Corresponding author
{z.albkhetan, d.plewczynski}@cent.uw.edu.pl

# General Methodology

The analysed genomic segments were represented using all available epigenomic and transcription factors assays conducted for the tested cell types See Supplementary Table 1.xlsx for full details of all features. MACS2 peaks calling method is invoked to find the assay peaks, we collect both the height of the peak (p-value) and the distance between the peak summit and center of the segment. Two features for each assay were calculated and assigned to each genomic segments. When more than one peak exist in the same genomic distance (which happens because of the variable peak length which is in general shorter than the genomic segment.), we either keep the maximum height and minimum distance when the resolution is high < 5 kb, or the sum of the features when the resolution is low 40 kb. The pairs of segments are formed combinatorically from all possible segments within the same topologically associating domain (TAD). We used the anchors features to represent the pair by using the minimum and maximum for each feature in both right and left anchors. Using the minimum and maximum for pair representation helps to have the same coding regardless the direction of the interaction (from the left anchor to the right or from the right to the left). We also added some additional features such as the genomic distance between the anchors, the distance to the TAD border. Genomic domains borders were defined according to CTCF-mediated ChIA-PET interactions from GM12878 cell line as Chromatin Contact Domains (CCDs). We used the same CCDs coordinates for other cell types because the TADs are typically conserved across the cell types and species (mammals). The confirmed interacting pairs were selected according to the analysis scheme, Hi-C, *in situ* Hi-C heatmaps, physical *in situ* Hi-C loops, and finally physical ChIA-PET interactions (CTCF/RNAP II). The available pairs are divided into training (80%) and testing (20%) datasets with some additional pre-processing applied before running machine learning algorithms (see below). The genomic segment size (i.e. the prediction resolution) may vary between the tests according to the availability, resolution and quality of the experimental data used for verification. Long-read ChIA-PET , and *in situ* Hi-C loops predictions are done using 1kb segment size, *in situ* Hi-C predictions for GM12878, K562, HUVEC, IMR90, NHEK and HMEC cell lines are performed using 5 kb bin sizes. In the case of the population data (Hi-C experiments of GM12878 and 7 individuals) the analysis was done using 40 kb genomic segment sizes. We use Random Forest as statistical learning algorithm, because of its efficiency and the easy interpretability of results. It uses many decision trees and the voting for the final predictions. Each individual tree is trained on a subset of the data, at each node the decision is made based on a subset of the features. Random Forest ranks the descriptive features according to their importance in data partitioning process into the target classes (in our case interacting and non-interacting pair). Such features ranking represents the complex and typically highly non-linear associations between one dimensional epigenomic data and three-dimensional spatial interactions. This ranking allows for biological inference of the most important epigenomic features that are relevant for three dimensional epigenome structure. To evaluate the predictor, we calculated the confusion matrix (the error matrix), which is one of the most important statistical metrics for evaluation of classifier performance. In the case of our binary classification problem, each sample belongs to one of the two possible categories being either an interaction or not interaction, and our aim is to predict its category. The performance of such predictor on the testing dataset can be summarized in the confusion matrix. The supplementary tables 3, 4, 5 and 6 include all detailed information about the confusion matrix in all genomic scales for all applied tests where the interactions are TP + FN, while the non-interactions are TN + FP.

We can measure our classifier performance using standard quality estimators such as accuracy, precision, sensitivity, specificity and false positive rate. The accuracy refers to the classifier’s capability to predict correctly, either as positive or negative:

$$accuracy= \frac{TP+TN}{TP+FP+TN+FN}$$

The Precision or Positive Predictive Value (PPV) refers to the correct prediction of positive values among all predictions as positive:

$$Precision= \frac{TP}{TP+FP}$$

The Sensitivity or True Positive Rate (TPR) represents the proportion of positives which are predicted as positive:

$$Sensitivity=TPR:= \frac{TP}{TP+FN}$$

Whereas the Specificity or True Negative Rate (TNR) refers to the proportion of negatives which are predicted as negative:

$$Specificity=TNR := \frac{TN}{TN+FP}$$

Finally, the False Positive Rate (FPR) is calculated as the proportion of negatives which are predicted as positives:

$$FPR= 1- Specificity= \frac{FP}{TN+FP}$$

We also computed **Receiver Operating Characteristic (ROC)** Curve which illustrates the performance of the binary classifier, whereas the area under the curve (AUC) represents the probability of a classifier to rank arbitrary chosen positive sample higher than arbitrary chosen negative sample (with the assumption that the positive samples have higher rank comparing with negative samples), i.e. the probability of a classifier to predict arbitrary chosen positive and negative pairs correctly.

# CTCF ChIA-PET Interactions Prediction

First, we trained our machine learning algorithm combining experimental interaction data for genomic domains (CCDs) from chromosomes and finally at the whole genome level. At the chromosome level, we analyze interacting segments from each chromosome separately to train specific predictor for each chromosome. The experimental data is than aggregated again to build the whole genome level predictor. The computational pipeline consists of two phases: first the segments filtration to avoid combinatorial explosion of pairs, and later the interactions training and prediction using supervised learning method.

## Segments Filtration

The aim of filtration procedure is to get the optimal subset of segments, which contains the majority of the interacting segments and as few as possible non-interacting ones.

The segments were processed by several filters sequentially:

1. First, we removed the segments which don’t have CTCF peaks, then we kept these ones that contain the summits of the CTCF peaks.
2. Secondly, we noticed by analyzing the distribution of the interacting segments according to their features, that the segments enriched by CTCF, SA1, RAD21, SMC3, and ZNF143 are more likely to interact, therefore we scored the segments according to each feature by calculating their p-values as the rank of the feature peak height divided by the segments count.
3. We calculated the final score as the multiplication of all important features scores then ranked and divided on the segments count.
4. The pairs of segments with p-value greater than 0.5 were removed (if all important features are available). Otherwise we filter using 0.8 for the threshold. The Figure 1 illustrates the distribution of the interacting and non-interacting segments according to their final score for GM12878 cell line, when all important features are available.
5. The last filtration is done to keep single candidate segment within each neighbouring segments (only when the distance between the neighbours is less than 3kb).

See the Supplementary File Supplementary Table 2.xlsx for the detailed statistics of the filtration pipeline.


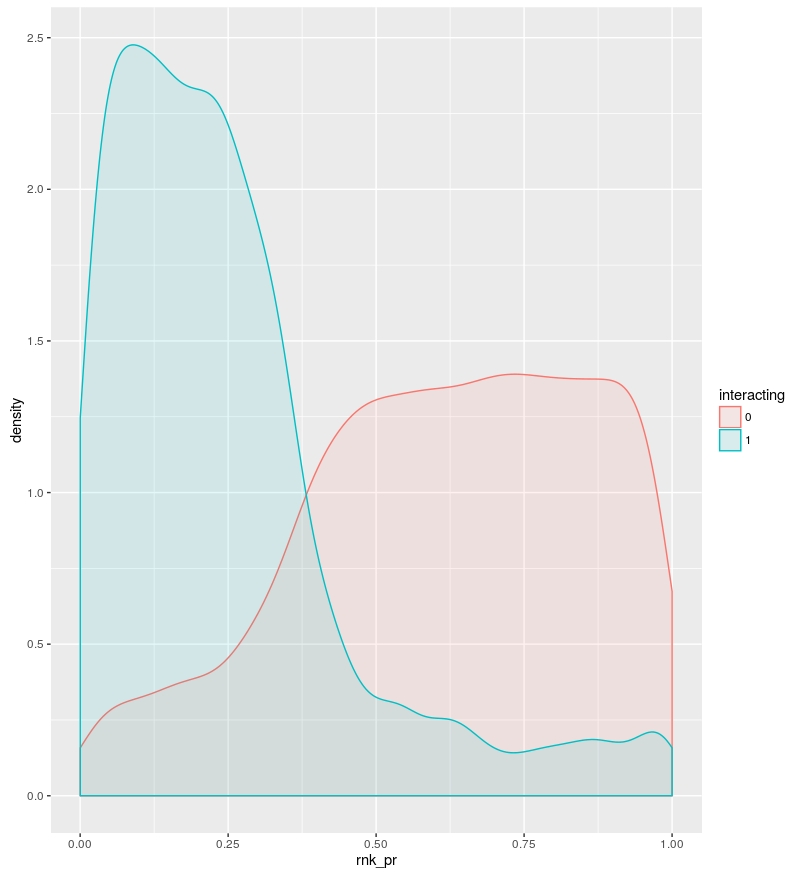


Figure 1 Interacting segments distribution according to the combined final score of the important features (CTCF, SA1, RAD21, SMC3, and ZNF143) in the X-axis. Y- Axis represents the density of the interacting (in green) and non-interacting (in red) segments

## Human lymphoblastoid GM12878 cell line analysis

### Input Features

**Transcription factors**: ATF2, ATF3, BATF, BCL11, BCL3, BCLAF, BHLHE40, BRCA1, CDP, CEBPB, CHD1, CHD2, cMYC, COREST, CREB1, CTCF, E2F4, EBF1, EBF, EGR1, ELF1, ELK1, ERRA, ETS1, EZH2, FOXM1, GABP, GCN5, IRF3, IRF4, JUND, MEF2A, MEF2C, MTA3, NFATC1, NFE2, NFIC, NRF1, NRSF, P300, PAX5,PBX3, PML, POL2-4H8, POL2-HAIB, POL2, POL2-S2P, POU2F2, PU.1, RAD21, RAD21-SNYDER, RFX5, RUNX3, RXRA, SA1, SIX5, SMC3, SP1, SPT20, SRF, STAT1, STAT3, STAT5, TAF1, TBP, TCF12, TCF3, USF1, USF2, WHIP, YY1, ZBTB33, ZEB1, ZNF143.

**Histone Modifications**: H2AFZ, H3K27ac, H3K27me3, H3K36me3, H3K4me1, H3K4me2, H3K4me3, H3K79me2, H3K9ac, H3K9me1, H3K9me3, and H4K20me1.

See (Supplementary Table 1.xlsx) for full details of all features and resources.

### Chromosomes Level Tests

The first test was done by splitting the set of pairs located within the same chromosome into the training (20% cases) and testing (80% pairs) datasets. For each chromosome we trained separate predictor and evaluated it within the same chromosome using testing dataset. We identify true positives, when the pair is predicted by *in silico* method and verified using long-read ChIAP-PET experiment, true negatives are the pairs that are not predicted by our method, yet which are reported by the experimental results. False positives are pairs that are predicted in our test but not observed in the experimental data, such pairs could be in principle observed when other experimental method is used. See the supplementary Excel file (Supplementary Table 3.xlsx) for detailed results.

In general, we found that the transcription factors are more important than epigenomic profiles for physical interactions identification. The top 25 important features of the predictors according to Random Forest classifier are: the distance between the anchors, the p-value of the combined score calculated based on the important features, SMC3, RAD21, CTCF, SA1, ZNF143 peaks heights in both anchors, the sum of important features heights, the p-values of RAD21, SA1, SMC3, CTCF, ZNF143 which are calculated in the domain level, and finally the order of the anchors in the TAD.

To see if the epigenomics and transcription factors pattern change among the chromosomes, we applied the predictors trained on one chromosome to the rest of the chromosomes of the same cell type. The evaluation of the performance shows that the prediction efficiency was affected slightly comparing with the prediction in the same chromosome. In general, our *in silico* RF predictors performed well, and such quality is retained for all chromosomes. The further details are provided in the supplementary file (Supplementary Table 3.xlsx).

### Genome Level Test

The final test was done at the whole genome level by aggregating interacting pairs for all chromosomes, splitting them into training (80%) and testing (20%) datasets. *In silico* predictor identifies 18826 interactions, 5251 of them were reported in the experimental data, 795 interactions reported in the same study were missed from our predictions. Genome level predictor achieved 0.814, 0.8, 0.86, and 0.908 for Accuracy, Specificity, Sensitivity and the area under the ROC Curve, when comparing with the experimental interactions. The Figure 2 present the ROC Curves for some predictors in different tests and levels. The importance rankings for features of genome-wide predictor are similar to chromosomes predictors. The importance plot is illustrated in the Figure 3.

| 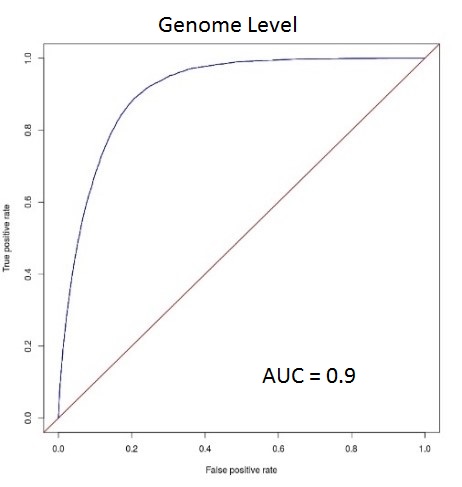 | 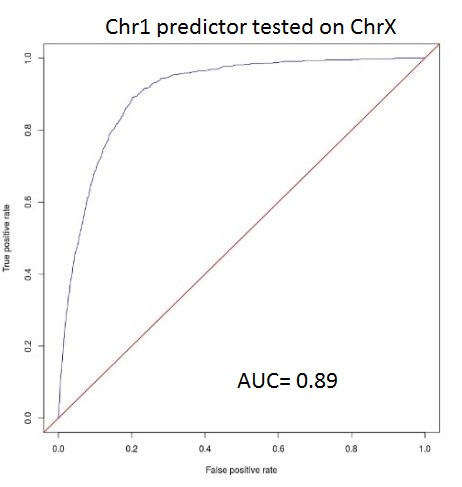 | 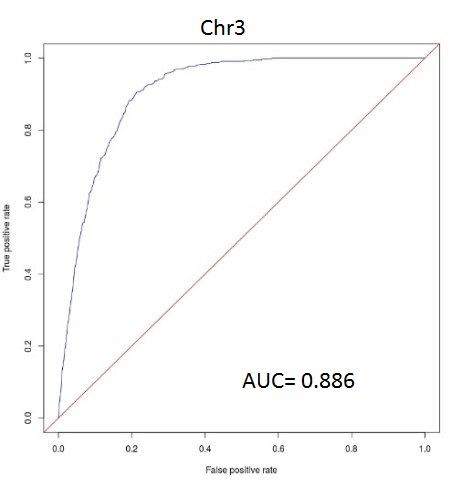 |
| --- | --- | --- |

*Figure 2. ROC curves for GM12878 cell line RF predictors at different levels, genome level, trained on chromosome chr1 and tested on chrX, and trained and tested on the same chromosome.*


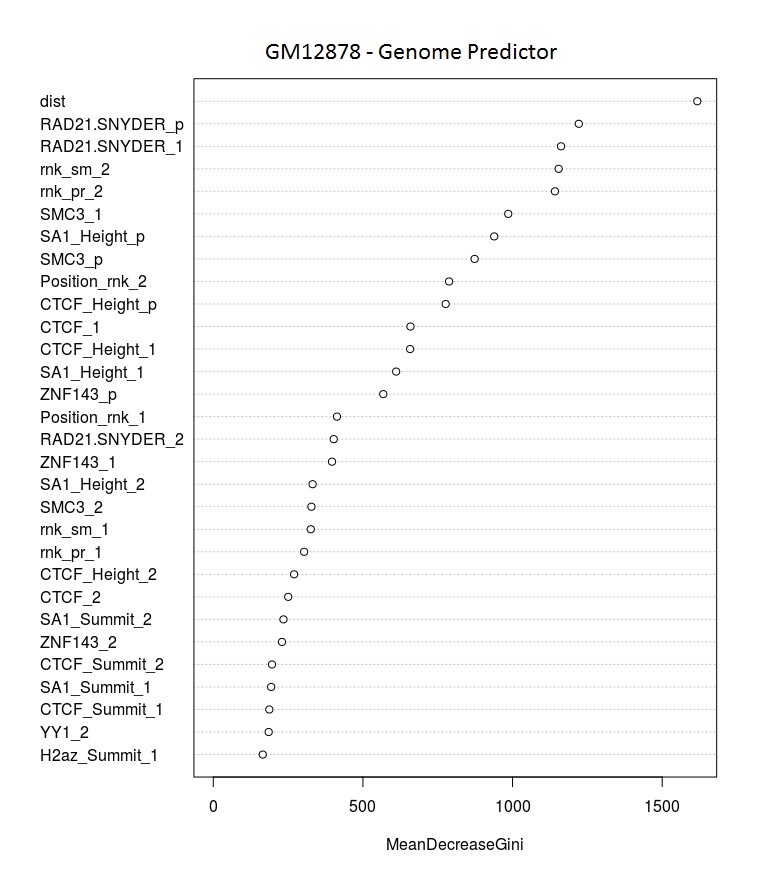


*Figure 3. The importance ranking of learning features for GM12878 the whole genome predictor. Y-axis represent the features, and X-Axis represents the mean decrease Gini which refers to the importance of this feature in data partitioning into interaction or non-interaction. The first part of the feature name refers to the feature name, the suffix, rnk_sum, rnk_pr: the sum and the multiplication of the important features scores. The Suffixes: 1 refers to the minimum 2: refers to the maximum. P: p-value calculated in the TAD level.*

## GM12878, K562, HeLa and MCF7 Cell Types Analysis

In the second phase of learning experiment, we constructed chromosome and genome levels predictors for all cell types using all available experimental data for them, then we used the largest subset of the epigenomic data that is shared between cell lines, in order to see the performance of our predictor when trained and tested on different cell types.

### Input Features in this test

**Transcription Factors:** CTCF, RAD21, YY1, SMC3, ZNF143, and NRS Transcription factors were used for GM12878, K562, and HeLa (Except YY1 for HeLa). CTCF, RAD21, and NRS for MCF7.

**Histone Modification:** H3K27ac, H3K27me3, H3K36me3, H3K4me1, H3K4me2, H3K4me3, H3K9ac, H3K9me3, H4K20me1, and H2AZ for GM12878, K562, and HeLa. While we used H3K27ac, H3K27me3, H3K36me3, H3K4me3, and H3K9me3 for MCF7 cell type.

See (Supplementary Table 1.xlsx) for full details of all features and their resources.

### Segments Filtration

The same filtration procedure as above was used in this test. Table 1 describes the results of the filtration pipeline for all cell types included in the test.

| Cell Line | All Segments | Interacting Segments | non-interacting Segments | All ChIA-PET Interactions^1^ | ChIA-PET in the studied domains^2^ | Covered ChIA-PET by filtered Segments^3^ |
| --- | --- | --- | --- | --- | --- | --- |
| K562 | 39835 | 22173 | 17662 | 42421 | 30978 | 26865 (87%) |
| HeLa | 42193 | 18668 | 23525 | 24080 | 19776 | 17695 (89%) |
| MCF7 | 42100 | 17138 | 24962 | 14978 | 13587 | 12326 (90%) |

Table 1 Filtration Results for K562, HeLA, and MCF7 cell types, 1) represent all experimental interactions reported for the specific cell line. 2) Represent the interactions that have both anchors related to the analysed TADs. 3) The interactions covered by the filtered segments.

See the supplementary file “Supplementary Table 2.xlsx” for detailed results.

### Chromosomes Level Test

The chromosome level predictors were evaluated on the same chromosome for the same cell type. The Table 2 present the average performance for all cell lines predictors.

| Cell line | Accuracy | Sensitivity | Specificity | AUC | All reported Experimentally | Predicted | Verified |
| --- | --- | --- | --- | --- | --- | --- | --- |
| GM12878 | 0.8217 | 0.8198 | 0.8419 | 0.906 | 6036 | 18040 | 5096 (84%) |
| K562 | 0.8346 | 0.8337 | 0.8584 | 0.9167 | 5410 | 26579 | 4663 (86%) |
| HeLa | 0.8581 | 0.8575 | 0.8807 | 0.938 | 3542 | 22219 | 3138 (88%) |
| MCF7 | 0.84 | 0.84 | 0.85 | 0.91 | 2459 | 24500 | 2130 (86%) |

*Table 2. Chromosomes predictors’ average performance obtained when testing chromosomes predictors.*

The detailed result for each cell type are provided in the supplementary file (Supplementary Table 3.xlsx).

**Importance ranking of features**

The cell types’ predictors shared similar important features mainly: the genomic distance between the anchors, RAD21, and CTCF peaks height in both anchors, the sum of important features heights, and the p-values of RAD21, CTCF which are calculated in the domain level. These predictors have different preferences for the histone modifications in their important features. H2AZ, H3K27ac, and H3K4me1 appear in the GM12878. H3K27ac, and H3K4me1 in K562. H2AZ, H3K4me1, H3K4me1, and H3K79me2 in HeLa. H3K27ac, H3K27me3, H3K36me3, H3K4me3, and H3K9me3 in MCF7 cell type.

*Table 3* present the average results for the chromosome predictors in the cell types (averaged over all 23 chromosomes) when testing each chromosome predictor on other chromosomes within the same cell type.

| Cell line | **Accuracy** | **Sensitivity** | **Specificity** | **AUC** |
| --- | --- | --- | --- | --- |
| GM12878 | 0.82 | 0.77 | 0.822 | 0.881 |
| K562 | 0.829 | 0.789 | 0.83 | 0.89 |
| HeLa | 0.857 | 0.84 | 0.857 | 0.92 |
| MCF7 | 0.8249 | 0.8246 | 0.8348 | 0.9077 |

*Table 3. Chromosomes predictors’ results tested on other chromosomes within the same cell line.*

Our test confirms that the epigenomic and transcription factors binding patterns in the interacting anchors are similar among different chromosomes within the same cell type. More details about chromosome-level results could be found in the supplementary file (Supplementary Table 3.xlsx).

### Genome Level Test

Genome level predictor was tested for the same cell types, and the performance evaluation is provided in the Table 4.

| Cell line | Accuracy | Sensitivity | Specificity | AUC | All reported Experimentally | Predicted | Verified |
| --- | --- | --- | --- | --- | --- | --- | --- |
| MCF7 | 0.884 | 0.884 | 0.893 | 0.95 | 2459 | 23038 | 2196 (89%) |
| HeLa | 0.866 | 0.864 | 0.911 | 0.948 | 3542 | 21404 | 3227 (91%) |
| K562 | 0.840 | 0.839 | 0.878 | 0.928 | 5410 | 26640 | 4751 (87%) |
| GM12878 | 0.82 | 0.816 | 0.865 | 0.911 | 6036 | 18409 | 5225 (86%) |

*Table 4. The whole genome level predictors evaluated for GM12878, HeLa, K562, and MCF7 cell types.*

Figure 4 illustrates ROC curves for these cell types’ predictors.

| 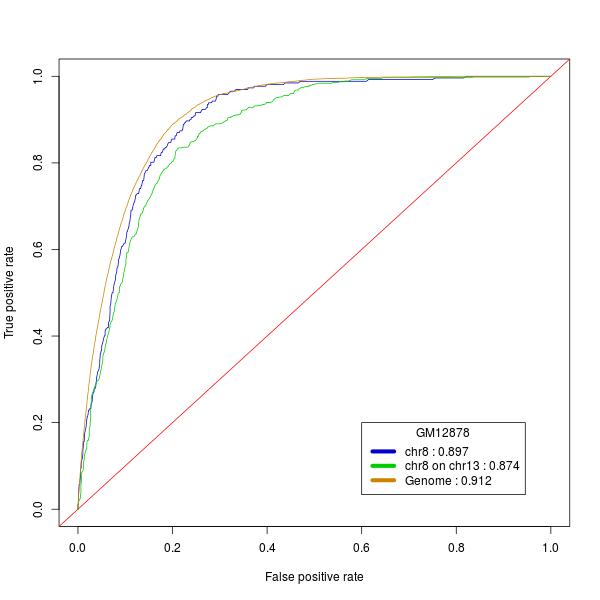 | 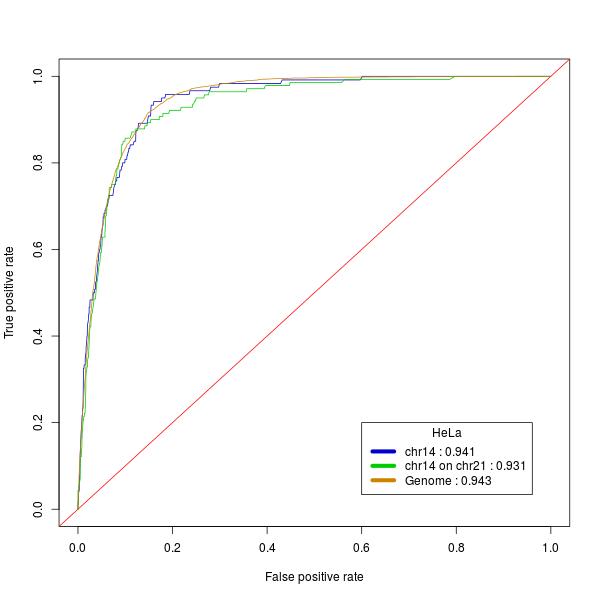 |
| --- | --- |
| 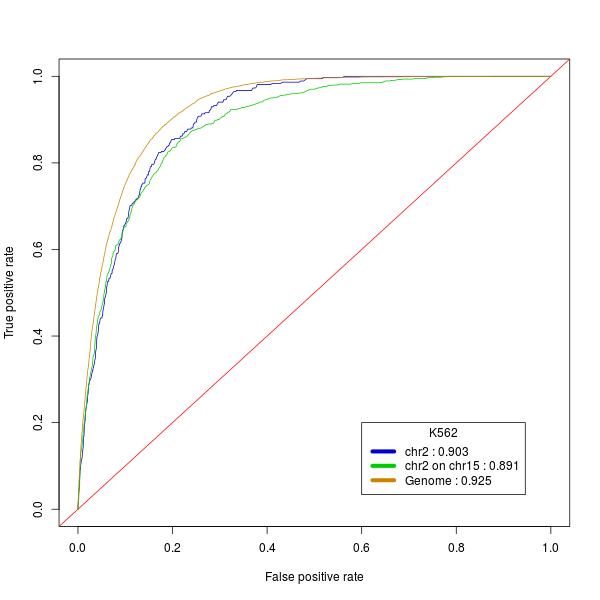 | 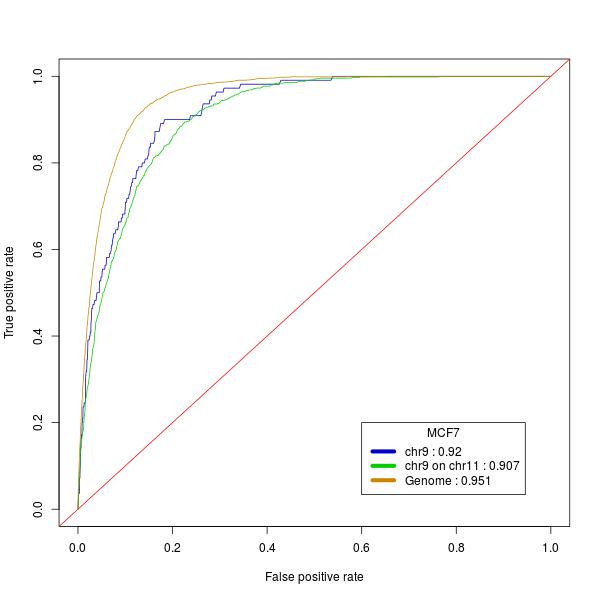 |

*Figure 4. ROC curves for GM12878, K562, HeLa, and MCF7 predictors at different scales: the whole genome in orange, trained on chromosome and tested on another chromosome in green, and trained and tested on the same chromosome in blue.*

**Important Features**

The important features for each genome predictor are illustrated on the Figure 5.


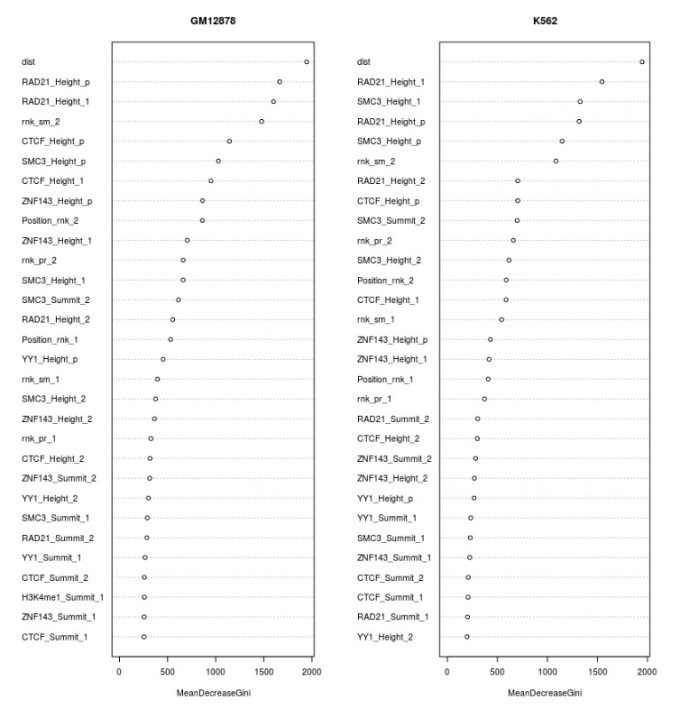


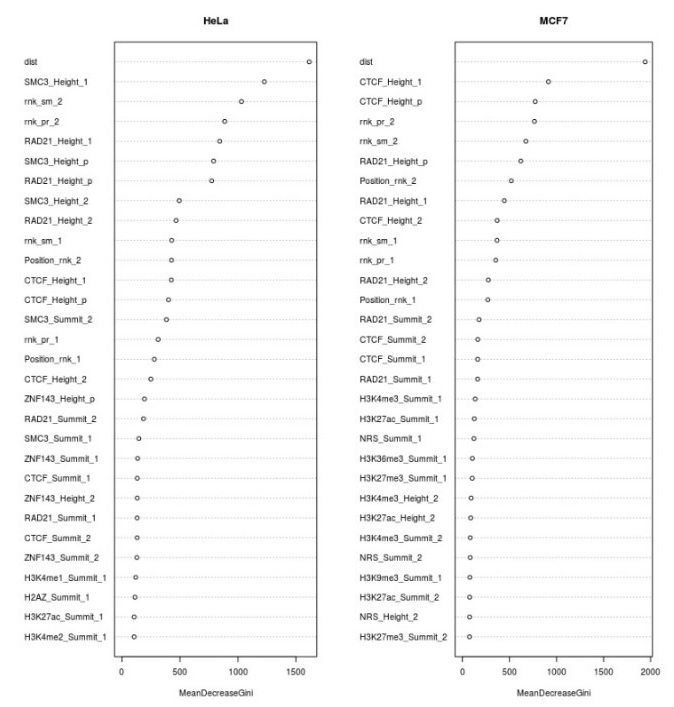


*Figure 5. The important features of the whole genome level predictors for GM12878, K562, HeLa, and MCF7 cell types.*

In all cell lines, the transcription factors were ranked higher than the histone modifications, and the most important features are similar and shared between the cell lines.

### Different Cell Lines

Further, we tested the machine learning predictors on new data from different cell types to check whether the epigenomic and transcription factors pattern differ among cell types. We trained the predictor using the shared subset of epigenomic profiles and transcription factors binding profiles, which affect the performance slightly. We tried all possible combinations of chromosome-chromosome, and genome-genome tests. The results (see Supplementary Table 3.xlsx) confirm the ability to predict ChIA-PET interactions using predictor trained on another cell type.

In the first case, we applied the predictor trained on single chromosome to the same chromosome, but from different cell type. These predictors achieved on average 0.8251, 0.8266, 0.7894, and 0.8912 for Accuracy, Sensitivity, Specificity, and the area under the ROC curve (AUC).

See Supplementary Table 3.xlsx for details.

**Importance Ranking of Features**

The predictors have 25 common features in their top importance ranking: the genomic distance between the anchors, NRS, RAD21, and CTCF peaks height in both anchors, the sum and multiplication of important features heights, the p-values of RAD21, CTCF which are calculated in the domain level, H3K27ac peaks height and finally the order of the segments in the CCD.

Further, we trained predictor for each cell type using interacting pairs from all chromosomes in genome-genome prediction test. Each cell type predictor was tested on the other cell types. ROC curves for these predictors are illustrated in the Figure 6. The important features for these predictors are illustrated in the Figure 7, these important features are similar to the reported ones in genome tests section, where the transcription factors are the most important features.

| 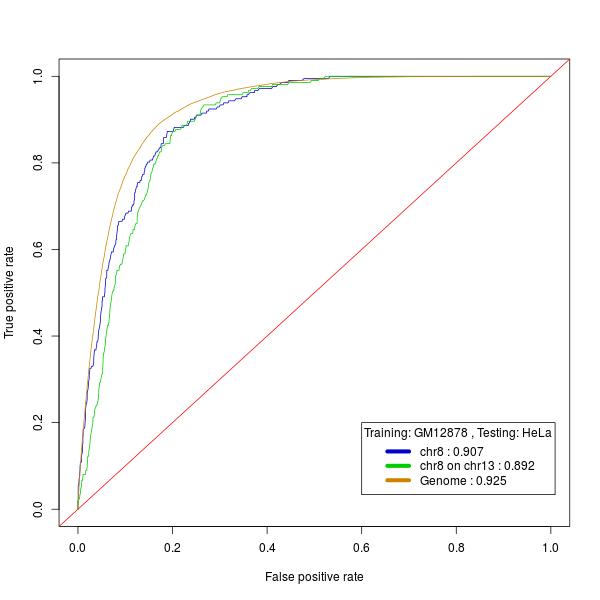 | 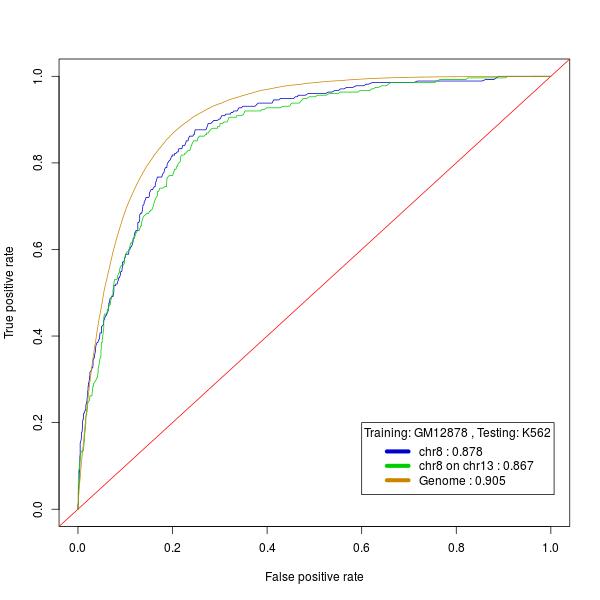 |
| --- | --- |
| 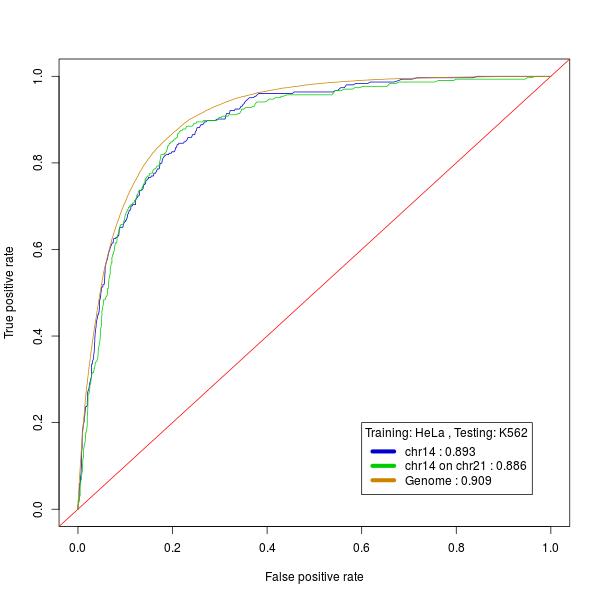 | 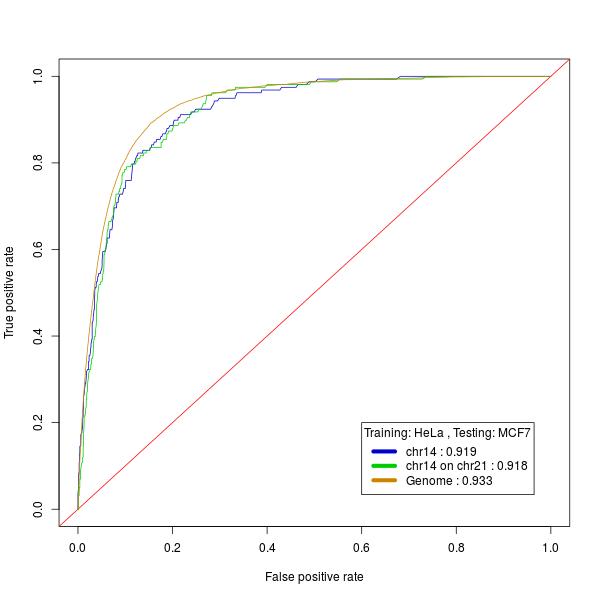 |
| 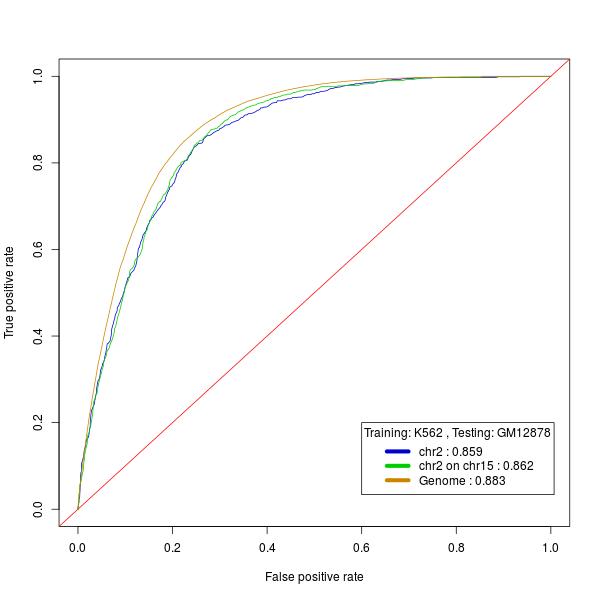 | 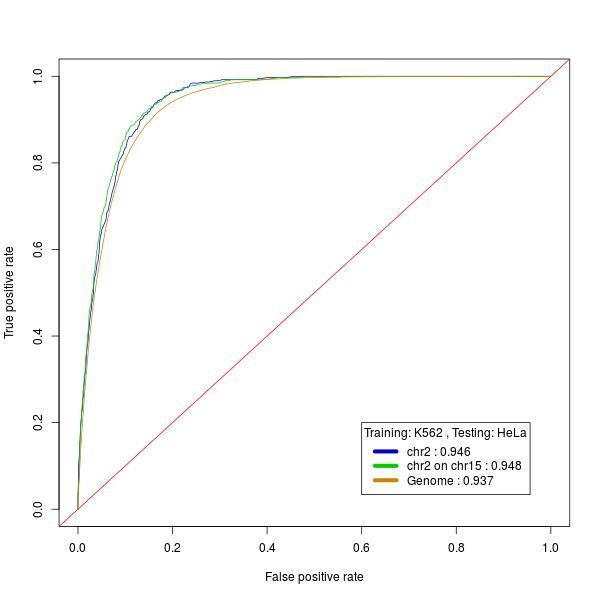 |
| 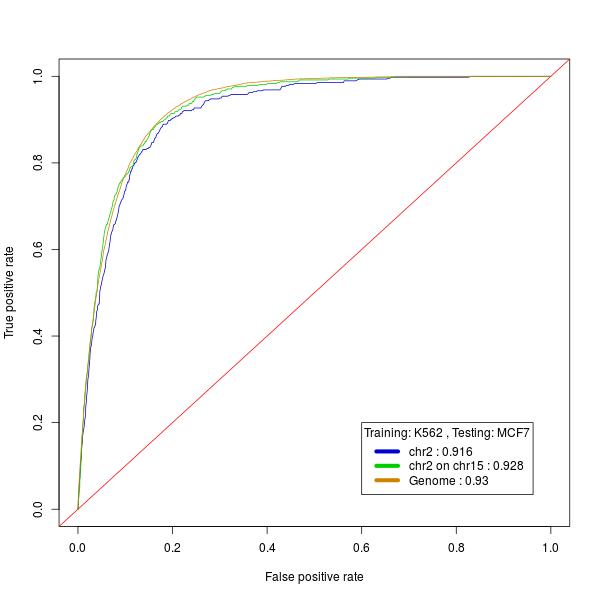 | 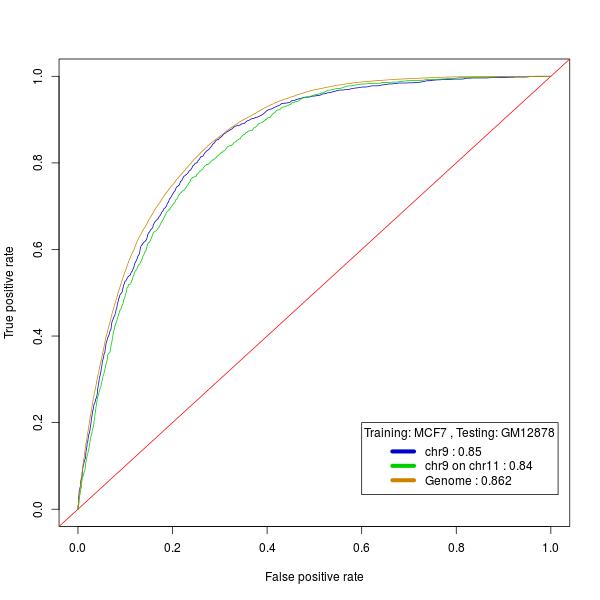 |
| 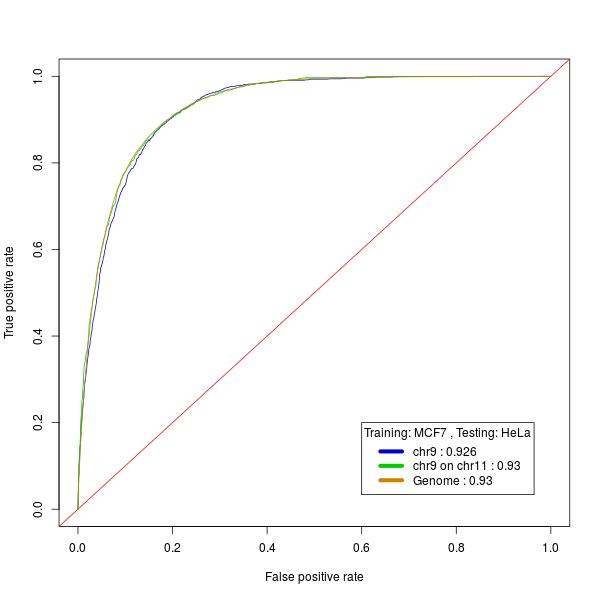 | 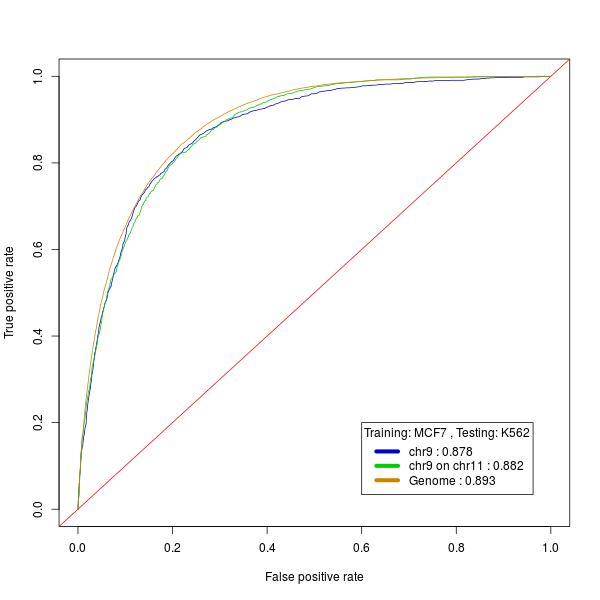 |
| 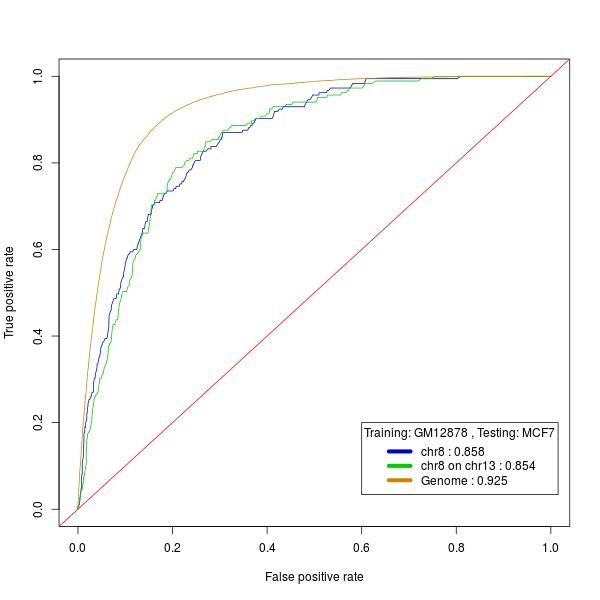 | 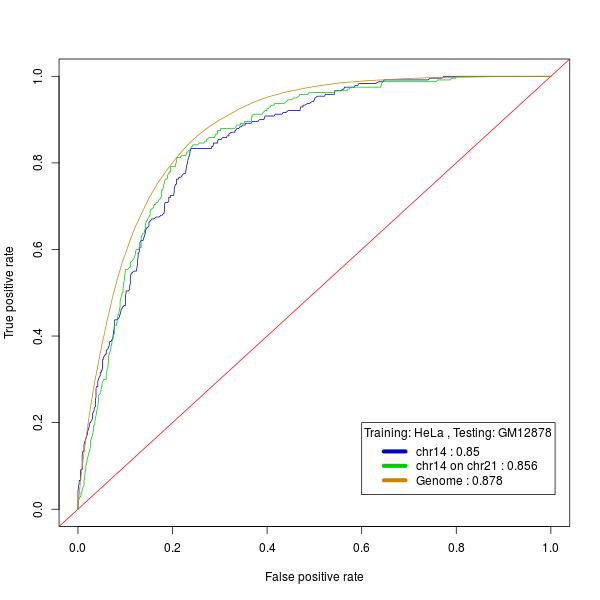 |

*Figure 6. ROC curves for various cell lines predictors trained on cell line, and tested on another cell line at different scales: the whole genome in orange, trained on chromosome and tested on another chromosome in green, and trained and tested on the same chromosome in blue.*


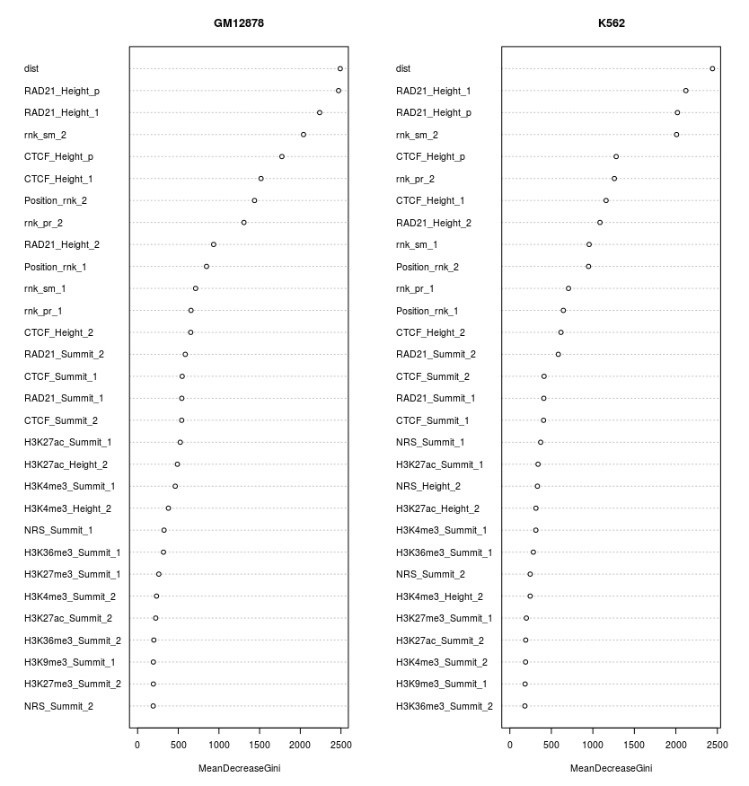


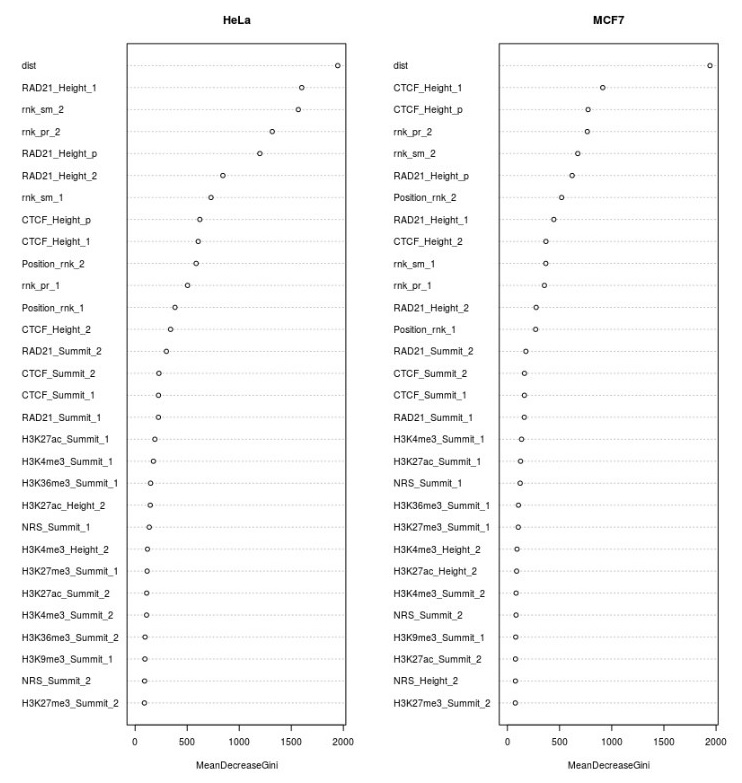


Figure 7 The important features of the whole-genome predictors for GM12878, K562, HeLa, and MCF7 cell lines using the same input data.

## Comparison with EpiTensor Unsupervised Method

We compared our supervised methodology with unsupervised approach of Epitensor to understand the similarities and differences between cell types and *in silico* algorithms for interactions identification. The cell types used in EpiTensor study don’t have ChIA-PET experimental results, so they reported the comparison of *in silico* predicted interactions with the ChIA-PET interactions from K562 cell type. In order to evaluate our supervised predictor performance with EpiTensor we downloaded all EpiTensor interaction for human embryonic stem cells (hESCs), TBL cells, MSCs, NPCs and human lung fibroblasts (IMR90) cells. In the case of our predictions, we used the genome level predictor trained on GM12878 to predict the interactions of K562. We considered the correct match between the interactions if both anchors of predicted pairs are overlapping with the anchors reported in the experimental ChIA-PET interactions. The experimental data reported 42421 interactions for the whole genome, 30978 of them have anchors that belong to our TADs, and have CTCF signal within. We aim to predict these 30978 interactions treating them as true positives. Our RF method predicted 93027 pairs in total, while EpiTensor predicted 420314 interactions for 5 different cell types, 210420 pairs of them are unique. When comparing with the experimental ChIA-PET interactions we found: 18945 of ChIA-PET interactions were predicted by our predictor, while 72186 pairs were not reported. 1331 of ChIA-PET interactions were predicted by EpiTensor while 209086 pairs were not reported. 922 pairs were predicted by our predictor and EpiTensor but they are not reported in the experimental interactions. Figure 8 illustrates the Venn diagram presenting the intersections between our prediction methodology, EpiTensor predictions, and the experimental ChIA-PET interactions for K562 cell line.


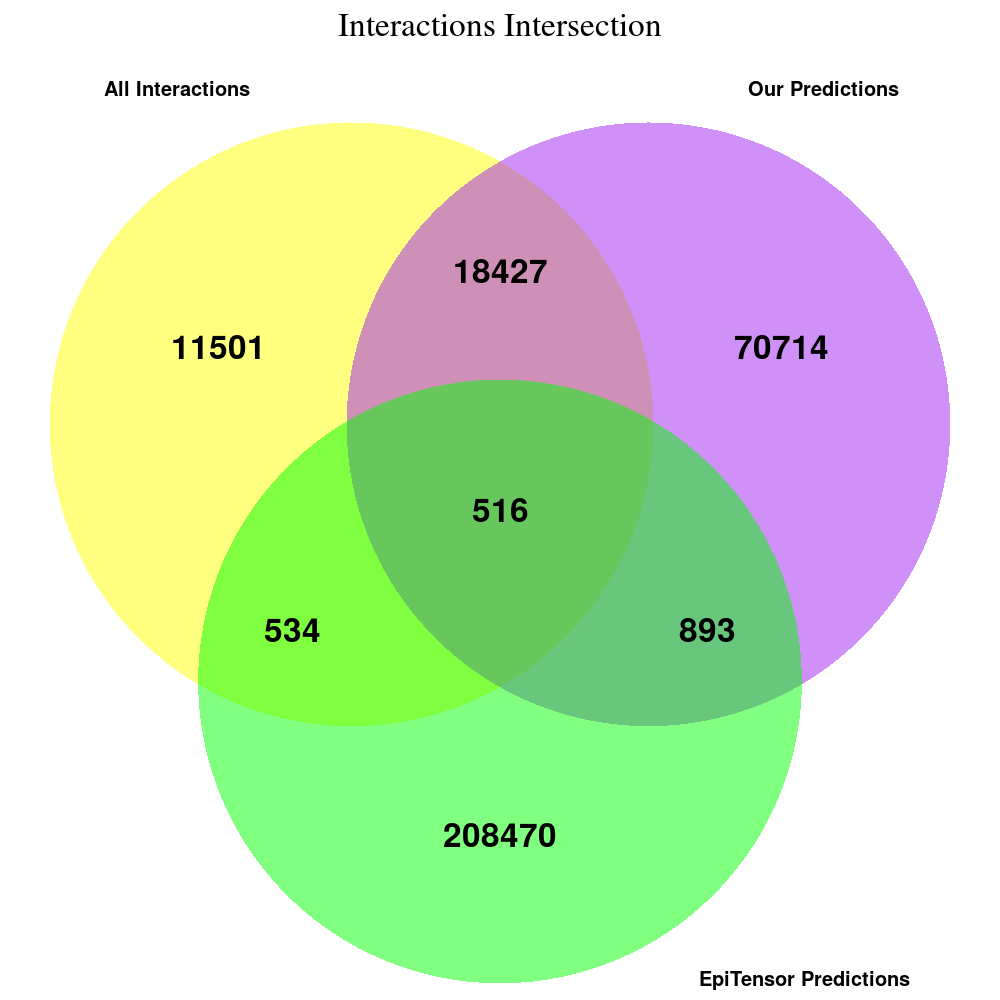


*Figure 8. K562 interactions comparison between our predictor (trained on GM12878 cell line), EpiTensor (constructed using human embryonic stem cells (hESCs), TBL cells, MSCs, NPCs and human lung fibroblasts cell lines) and experimental ChIA-PET interactions for K562 cell line.*

# Physical *in situ* Hi-C Chromatin Loops prediction

## GM12878 Analysis (All Features included)

*In situ* Hi-C chromatin loops are subset of the *in situ* Hi-C heatmaps, which are more believed to be physical interactions. These loops represent the contacts in the heatmaps which have high score comparing with the interactions between the surrounding loci.

To see if the predictor is able to recover *in situ* Hi-C chromatin loops, we used similar approach as before with the same epigenomics and transcription factors as in CTCF ChIA-PET interactions prediction phase. In this experiment, we used the Hi-C loops, which contain CTCF signal in their anchors, as positives in our training phase.

### Input Features

**Transcription factors**: ATF2, ATF3, BATF, BCL11, BCL3, BCLAF, BHLHE40, BRCA1, CDP, CEBPB, CHD1, CHD2, cMYC, COREST, CREB1, CTCF, E2F4, EBF1, EBF, EGR1, ELF1, ELK1, ERRA, ETS1, EZH2, FOXM1, GABP, GCN5, IRF3, IRF4, JUND, MEF2A, MEF2C, MTA3, NFATC1, NFE2, NFIC, NRF1, NRSF, P300, PAX5,PBX3, PML, POL2-4H8, POL2-HAIB, POL2, POL2-S2P, POU2F2, PU.1, RAD21, RAD21-SNYDER, RFX5, RUNX3, RXRA, SA1, SIX5, SMC3, SP1, SPT20, SRF, STAT1, STAT3, STAT5, TAF1, TBP, TCF12, TCF3, USF1, USF2, WHIP, YY1, ZBTB33, ZEB1, ZNF143.

**Histone Modifications**: H2AFZ, H3K27ac, H3K27me3, H3K36me3, H3K4me1, H3K4me2, H3K4me3, H3K79me2, H3K9ac, H3K9me1, H3K9me3, and H4K20me1.

See (Supplementary Table 1.xlsx) for full details of all features.

### Chromosome Level Tests

We started the analysis using GM12878 cell type with all available epigenomics and transcription factor binding profiles. The filtration pipeline provides 32378 genomic segments pairs, which cover 4357 of 5077 (~ 86 %) *in situ* Hi-C loops which belong to the studied CCDs and contain CTCF peaks. The chromosomes predictors achieved on average: 0.7773, 0.7778, 0.7467, and 0.84 for Accuracy, Specificity, Sensitivity and the area under ROC curve (AUC). See the supplementary file (Supplementary Table 4.xlsx) for more details.

Additionally, we tested each chromosome-specific predictor on the other chromosomes. Such predictors achieved on average 0.7727, 0.7734, 0.7164, and 0.8187 for Accuracy, Specificity, Sensitivity and the area under ROC curve. The results for each predictor were described in the supplementary file (Supplementary Table 4.xlsx.xlsx).

The top 25 important features for chromosome predictors are: the genomic distance between the anchors, RAD21, SA1, SMC3, ZNF143, YY1, ZEB1, ELF1, EGR1, CTCF, and H3K79me2 peaks height in both anchors, the sum and the multiplication of the important features heights scores, and the p-values of RAD21, CTCF, ZNF143, and SMC3 which are calculated in the domain level, finally the order of the segments within a genomic domain.

### Genome Level Tests

The whole genome predictor which trained on the interacting pairs collected from all chromosomes achieved 0.798, 0.798, 0.768, and 0.864 for Accuracy, Specificity, Sensitivity and the area under the ROC curve. The important features of genome predictor are presented on the Figure 9. The ROC curves for these predictors at different levels are illustrated in the Figure 10.


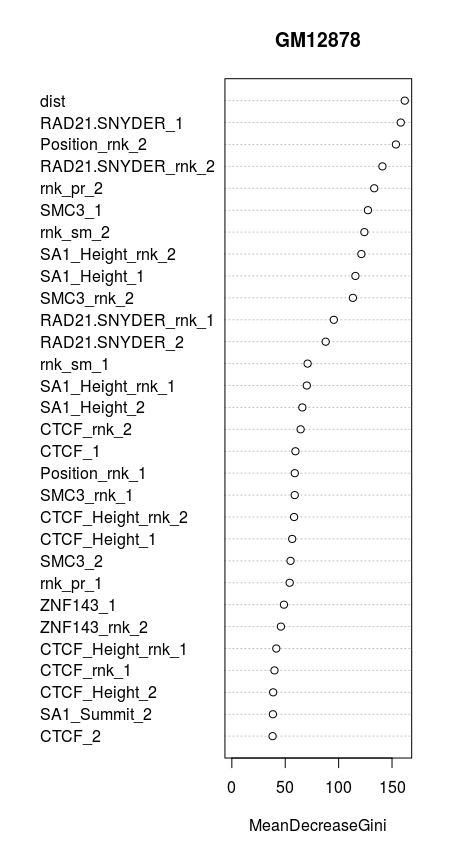


Figure 9. The important features, reported for GM12878 cell type predictor trained and tested on the whole genome.

| 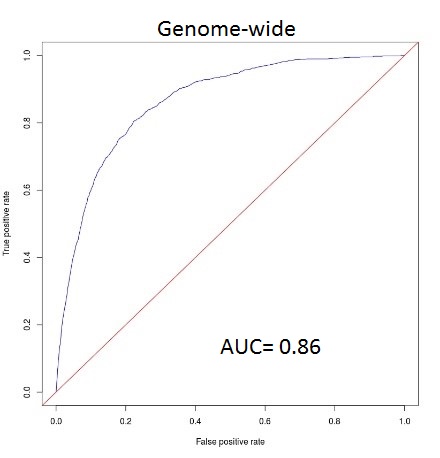 | 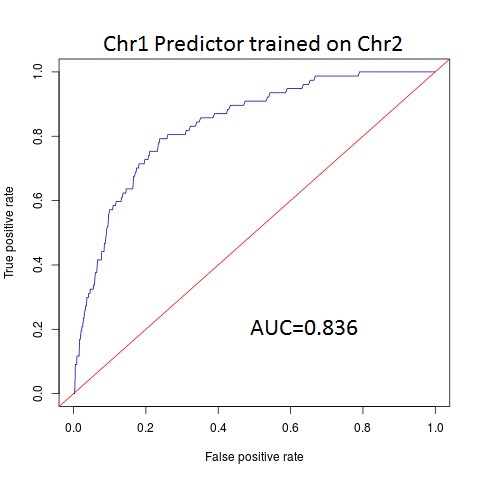 | 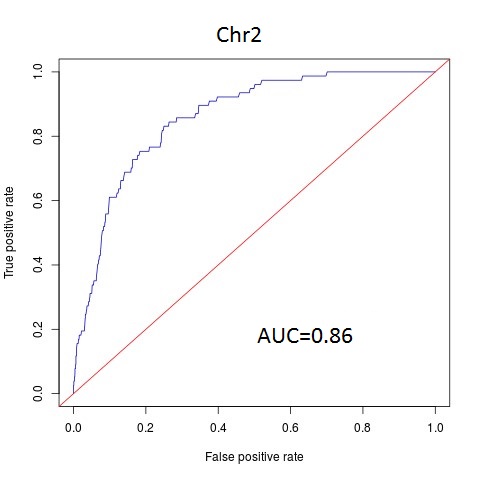 |
| --- | --- | --- |

Figure 10. ROC Curves obtained for GM12878 cell types, in different genomic scales, Trained and tested on the same chromosome, trained on chromosome and tested on another chromosome, trained and tested on the whole genome.

## GM12878, K562, HeLa, IMR90, HMEC, NHEK and HUVEC *in situ* Hi-C chromatin loops prediction

We tested our supervised predictors on different cell lines in order to evaluate their performance across cell types. According to the availability of the epigenomics and transcription factors data we trained GM12878, K562 and HeLa predictors on the common and shared subset of features. The same procedure was performed also for the cell types HUVEC, MHEK, and HMEC.

### Input Features in this test

**Transcription factors**: CTCF, RAD21, YY1, SMC3, ZNF143, and NRS.
**Histone Modifications**: EZH2. H3K27ac, H3K27me3, H3K36me3, H3K4me1, H3K4me2, H3K4me3, H3K9ac, H3K9me3, H4K20me1, H2AZ, H3k79me2.

See (Supplementary Table 1.xlsx) for full details of all features.

### Chromosome Level Tests

Chromosome-level predictors tested on the same chromosomes achieved in average: 0.781, 0.782, 0.77, and 0.852 for Accuracy, Specificity, Sensitivity, and the area under ROC Curve. Table 5 describes the results for each cell line. *In situ* Hi-C loops are smaller in number comparing with ChIA-PET interactions for the same cell type. This observation affects the prediction performance slightly. Detailed results are provided in the supplementary (Supplementary Table 4.xlsx).

| Cell Type | **Accuracy** | **Specificity** | **Sensitivity** | **AUC** |
| --- | --- | --- | --- | --- |
| GM12878 | 0.848 | 0.850 | 0.643 | 0.815 |
| K562 | 0.831 | 0.833 | 0.636 | 0.852 |
| HeLa | 0.747 | 0.746 | 0.879 | 0.901 |
| IMR90 | 0.779 | 0.779 | 0.808 | 0.840 |
| HUVEC | 0.751 | 0.751 | 0.806 | 0.829 |
| HMEC | 0.776 | 0.775 | 0.933 | 0.913 |
| NHEK | 0.765 | 0.766 | 0.74 | 0.831 |

Table 5. Chromosomes predictors’ performance evaluation for GM12878, K562, HeLa, IMR90, HUVEC, HMEC, NHEK cell lines.

The top 25 important features of the first cell types group (GM12878, K562, HeLa) were covered by: the distances between the anchors, RAD21, SMC3, ZNF143, NRS, CTCF, H3K4me1, H3K4me2, H3K27ac, H4K20me1, H3K9ac, H3K27me3, H3K4me3, and H3K79me2 peaks height in both anchors, the sum and the multiplication of the important features heights scores, and the p-values of RAD21, ZNF143, and SMC3 which are calculated in the domain level, finally the order of the segments within the TAD.

The top 25 important features of the second group (IMR90, HUVEC, HMEC, and HNEK, K562, HeLa) predictors are: the distance between the anchors, CTCF, H3K4me1, H3K4me2, H3K27ac, H4K20me1, H3K9ac, H3K27me3, H3K4me3, EZH2, H3K9me3, H2AZ, and H3K79me2 peaks height in both anchors, the sum and the multiplication of the important features heights scores, and the p-values of CTCF which are calculated in the domain level, finally the order of the segments within the CCD.

The second group of cell lines does not have many Transcription factors - only CTCF binding profile is included, yet we are still able to predict physical interactions with good performance. We used chromosomes predictors to identify the interactions within the rest of chromosomes. The results are presented in the Table 6. Detailed results are in the supplementary file (Supplementary Table 4.xlsx).

| Cell Type | **Accuracy** | **Specificity** | **Sensitivity** | **AUC** |
| --- | --- | --- | --- | --- |
| K562 | 0.886 | 0.888 | 0.509 | 0.781 |
| HeLa | 0.725 | 0.725 | 0.657 | 0.752 |
| IMR90 | 0.921 | 0.924 | 0.412 | 0.799 |
| HUVEC | 0.563 | 0.562 | 0.862 | 0.802 |
| HMEC | 0.635 | 0.634 | 0.713 | 0.76 |
| NHEK | 0.776 | 0.777 | 0.411 | 0.695 |
| K562 | 0.886 | 0.888 | 0.509 | 0.781 |

Table 6. Chromosome-level predictor results, when tested on another chromosome for the same cell line.

### Genome Level Tests

Genome level predictors are built and tested on the same cell types. Table 7 provides the evaluation of these predictors that show the success of the predictor in interactions predictions, and the percentage of the covered experimental interactions.

| Cell Type | Predicted | Verified | All Reported | Accuracy | Specificity | Sensitivity | AUC |
| --- | --- | --- | --- | --- | --- | --- | --- |
| GM12878 | 14693 | 771 (77%) | 1000 | 0.819 | 0.819 | 0.771 | 0.874 |
| K562 | 25877 | 971 (77%) | 1257 | 0.822 | 0.822 | 0.772 | 0.876 |
| HeLa | 27844 | 757 (80%) | 946 | 0.803 | 0.803 | 0.800 | 0.881 |
| IMR90 | 13091 | 717 (79%) | 899 | 0.816 | 0.816 | 0.798 | 0.886 |
| HUVEC | 20431 | 731 (73%) | 988 | 0.791 | 0.792 | 0.740 | 0.848 |
| HMEC | 12275 | 428 (0.76) | 552 | 0.744 | 0.743 | 0.775 | 0.832 |
| NHEK | 29484 | 1077 (79%) | 1363 | 0.801 | 0.801 | 0.790 | 0.875 |

Table 7. Genome Level evaluation results.

The important features of genome levels predictors are illustrated in Figure 11, Figure 12, and Figure 13.

Figure 14 illustrates the ROC curves for these predictors at different levels.


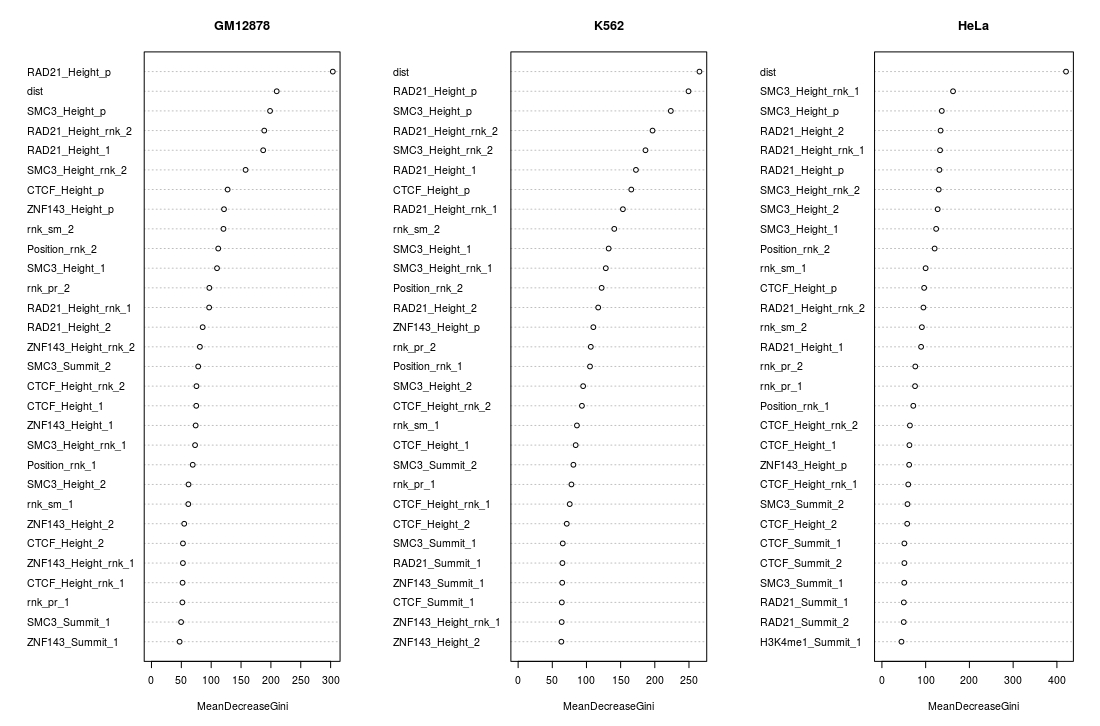


Figure 11 important features according to the predictors trained on the cell lines: GM12878, K562, and HeLa


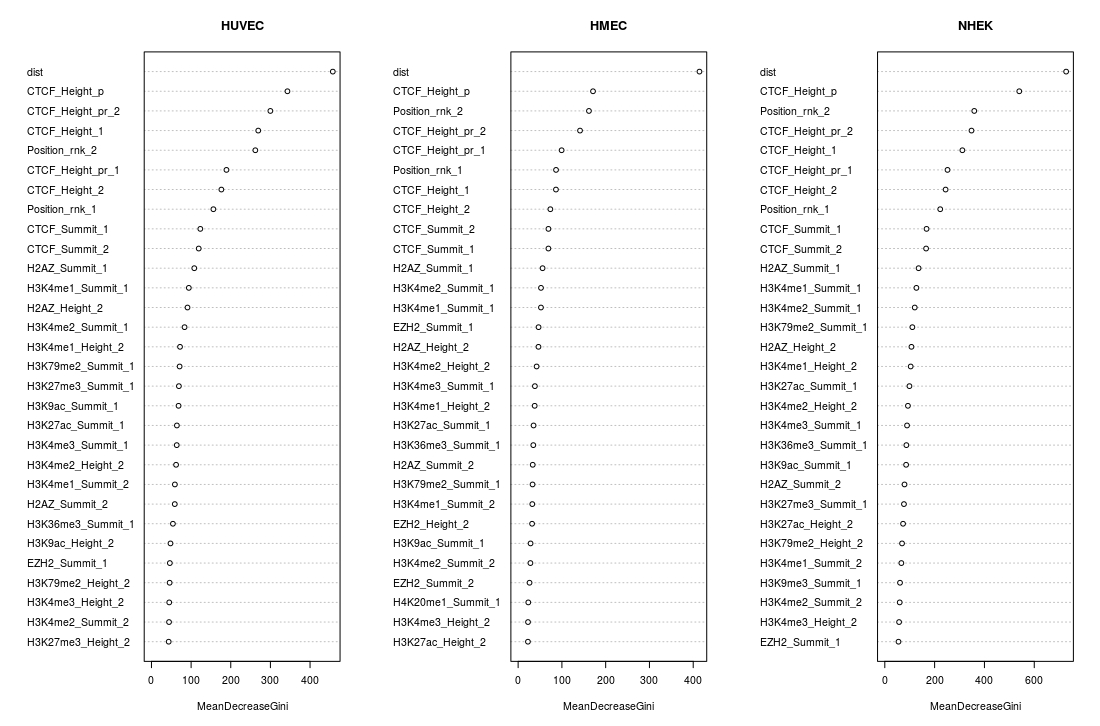


Figure 12. The important features according to the predictors trained on the cell lines: HUVEC, HMEC, and NHEK.


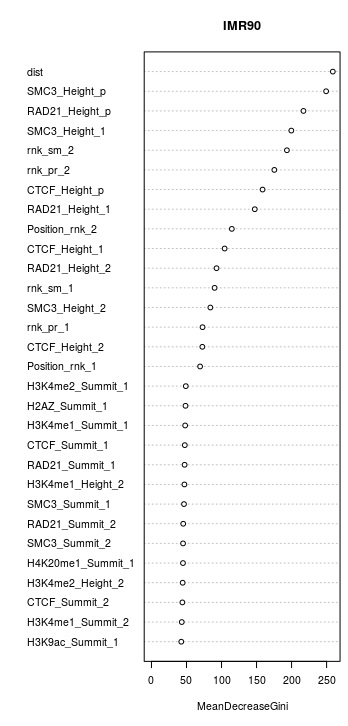


Figure 13. The important features according to the predictors trained on the cell lines: IMR90

| 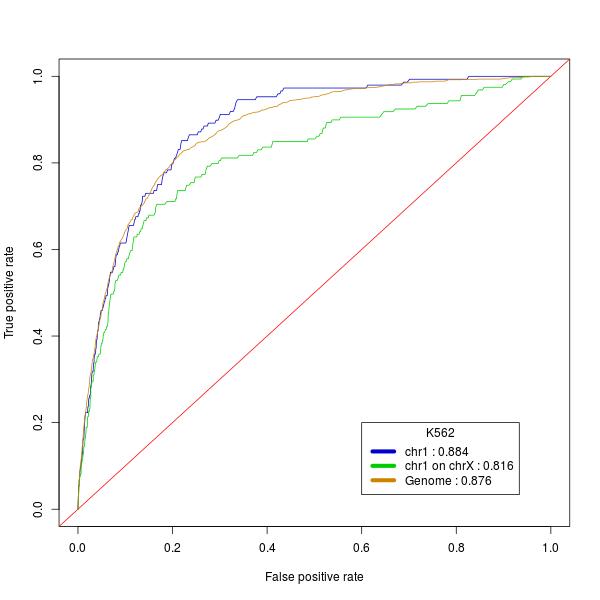 | 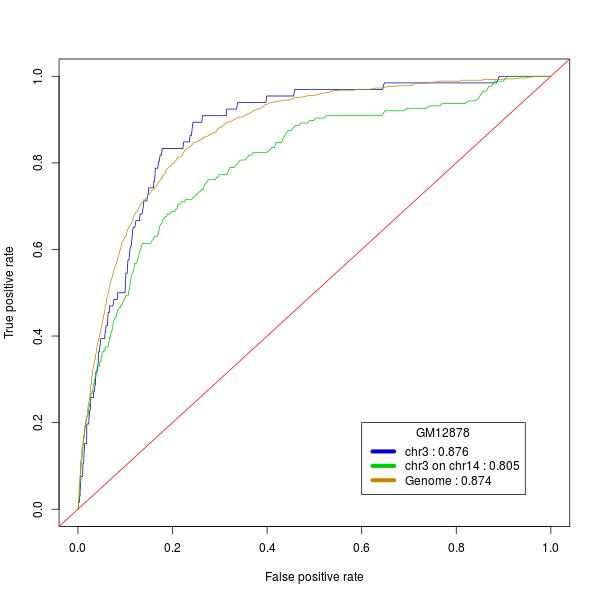 |
| --- | --- |
| 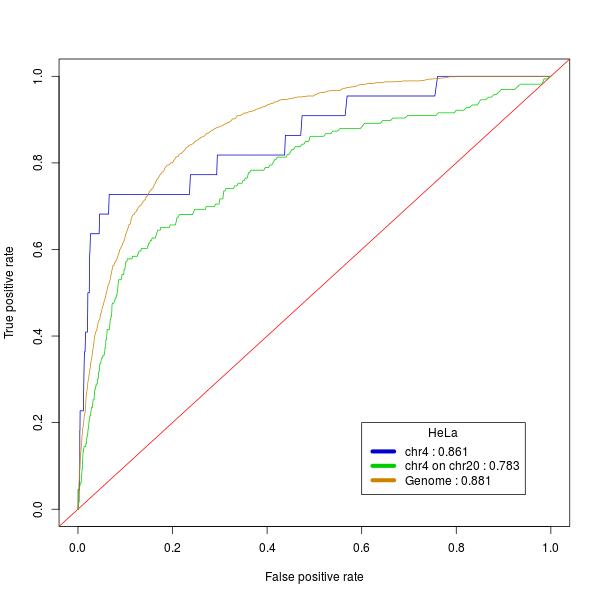 | 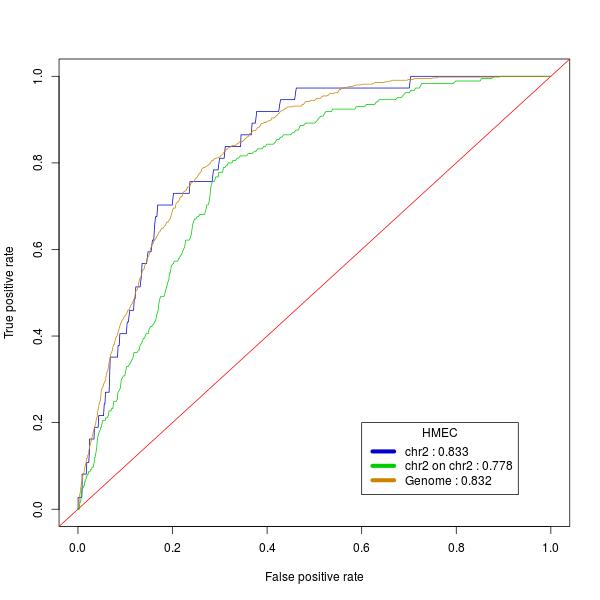 |
| 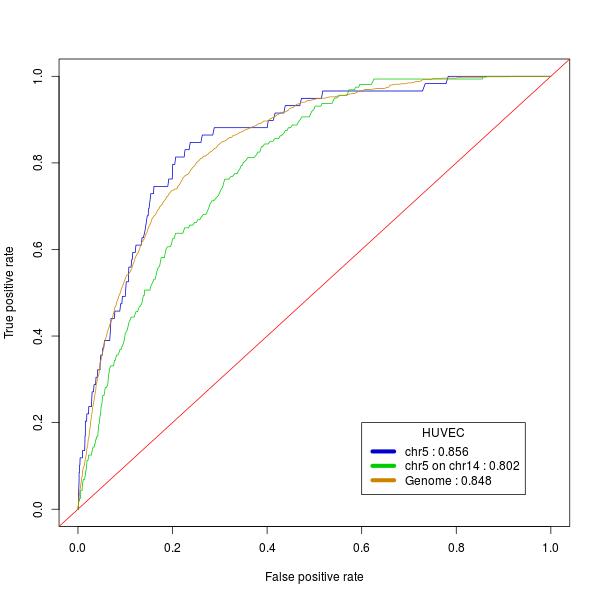 | 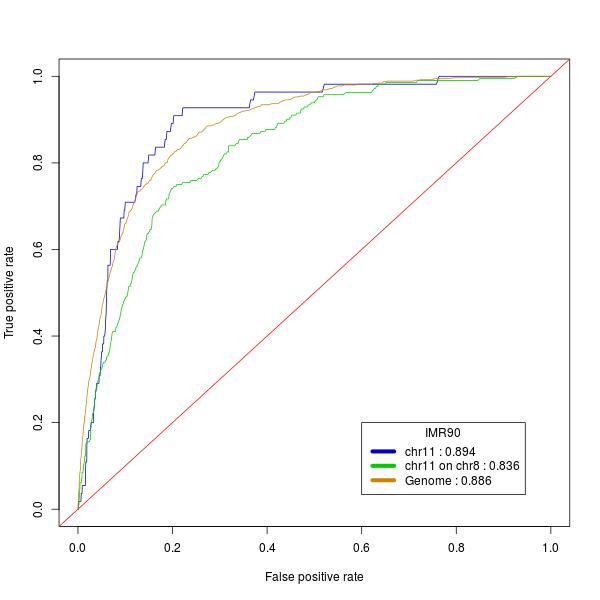 |
| 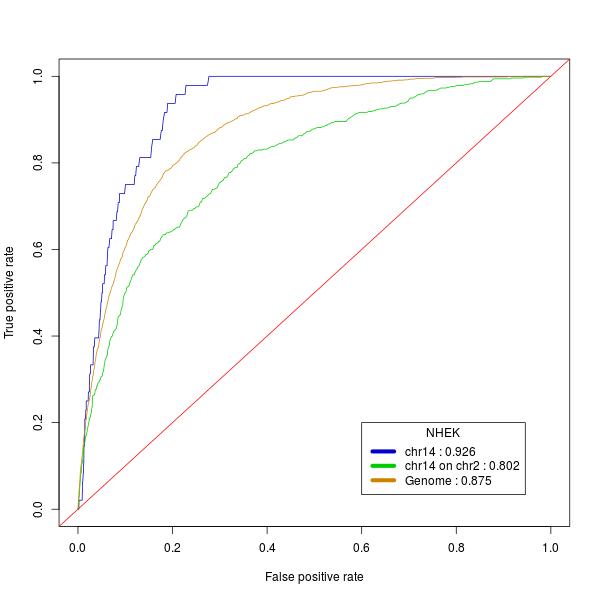 | |

*Figure 14. ROC Curves, obtained by the predictors trained on the cell lines: GM12878, K562, HeLa, HUVEC, HMEC, NHEK, and IMR90. Three curves for each cell line representing the predictors in different genome scales the whole genome in orange, trained on chromosome and tested on another chromosome in green, and trained and tested on the same chromosome in blue.*

We applied chromosome-chromosome and genome-genome tests to evaluate the performance of each predictor when testing on different cell type, without any prior knowledge about the tested cell type specific interactions. All combinations of the cell types belong to the same subgroup were tested. Group 1: GM12878, K562, and HeLa. Group 2: HUVEC, NHEK, and HMEC. This division is related to the availability of the epigenomics profiles and transcription factors. Each group has the same set of the features. In chromosome-chromosome tests we evaluated each chromosome predictor on the same chromosome but different cell type. Table 8 illustrate the tests results. Detailed results are available in the supplementary (Supplementary Table 4.xlsx).

| Group | Training Cell Type | Testing Cell Type | Accuracy | Specificity | Sensitivity | AUC |
| --- | --- | --- | --- | --- | --- | --- |
| 1 | GM12878 | K562 | 0.875 | 0.878 | 0.552 | 0.804 |
| 1 | GM12878 | HeLa | 0.866 | 0.868 | 0.562 | 0.802 |
| 1 | K562 | GM12878 | 0.712 | 0.711 | 0.805 | 0.835 |
| 1 | K562 | HeLa | 0.757 | 0.758 | 0.710 | 0.807 |
| 1 | HeLa | GM12878 | 0.769 | 0.769 | 0.714 | 0.825 |
| 1 | HeLa | K562 | 0.829 | 0.831 | 0.599 | 0.800 |
| 2 | HUVEC | HMEC | 0.739 | 0.741 | 0.601 | 0.761 |
| 2 | HUVEC | NHEK | 0.822 | 0.824 | 0.588 | 0.793 |
| 2 | HMEC | HUVEC | 0.789 | 0.791 | 0.577 | 0.766 |
| 2 | HMEC | NHEK | 0.819 | 0.822 | 0.515 | 0.761 |
| 2 | NHEK | HUVEC | 0.744 | 0.744 | 0.714 | 0.804 |
| 2 | NHEK | HMEC | 0.653 | 0.653 | 0.702 | 0.748 |

Table 8. Chromosome-level predictor evaluation when testing on other cell types.

Genome-genome predictors test is also performed for the each group cell types and described in the Table 9.

| Training Cell Type | Testing Cell Type | predicted | Verified | All Reported | Accuracy | Specificity | Sensitivity | AUC |
| --- | --- | --- | --- | --- | --- | --- | --- | --- |
| HeLa | K562 | 112026 | 4073 | 6324 | 0.844 | 0.846 | 0.644 | 0.829 |
| HeLa | GM12878 | 83883 | 3814 | 5043 | 0.792 | 0.792 | 0.756 | 0.852 |
| GM12878 | K562 | 76694 | 3593 | 6324 | 0.893 | 0.896 | 0.568 | 0.820 |
| GM12878 | HeLa | 77324 | 2656 | 4779 | 0.889 | 0.891 | 0.556 | 0.821 |
| K562 | HeLa | 153302 | 3406 | 4779 | 0.781 | 0.782 | 0.713 | 0.824 |
| K562 | GM12878 | 101127 | 4152 | 5043 | 0.749 | 0.748 | 0.823 | 0.860 |
| NHEK | HMEC | 80133 | 2016 | 2802 | 0.662 | 0.662 | 0.719 | 0.753 |
| NHEK | HUVEC | 122458 | 3749 | 4981 | 0.749 | 0.749 | 0.753 | 0.829 |
| HUVEC | HMEC | 61677 | 1805 | 2802 | 0.740 | 0.741 | 0.644 | 0.774 |
| HUVEC | NHEK | 121369 | 4326 | 6862 | 0.834 | 0.836 | 0.630 | 0.825 |
| HMEC | NHEK | 137172 | 4073 | 6862 | 0.812 | 0.814 | 0.594 | 0.786 |
| HMEC | HUVEC | 108269 | 3218 | 4981 | 0.777 | 0.778 | 0.646 | 0.793 |

Table 9. Genome level predictors evaluation when testing on other cell types.

Figure 15 illustrate the ROC curves for chromosome-chromosome, and genome-genome tests.

| 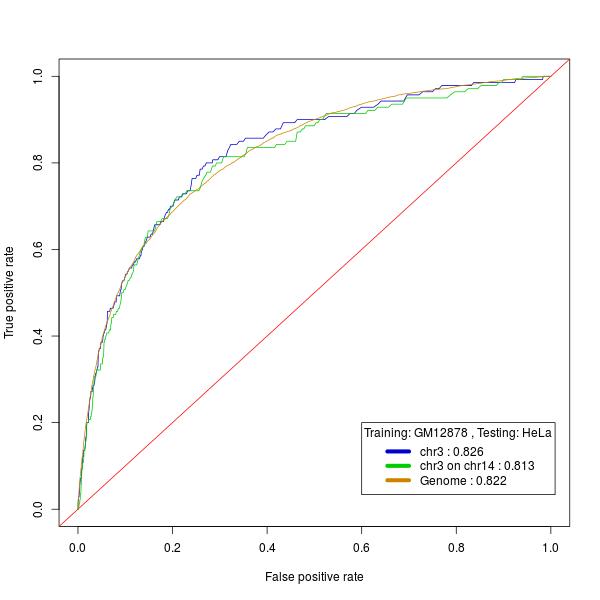 | 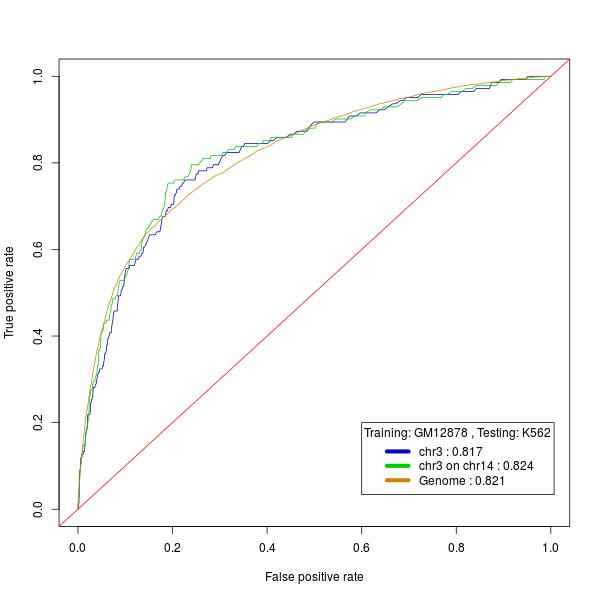 |
| --- | --- |
| 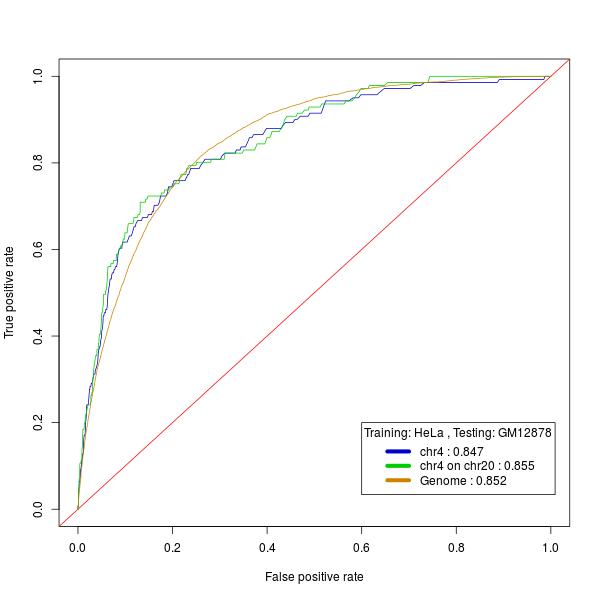 | 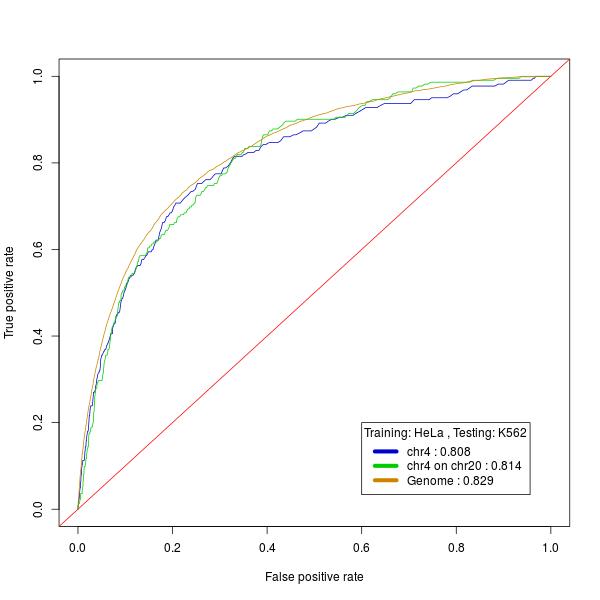 |
| 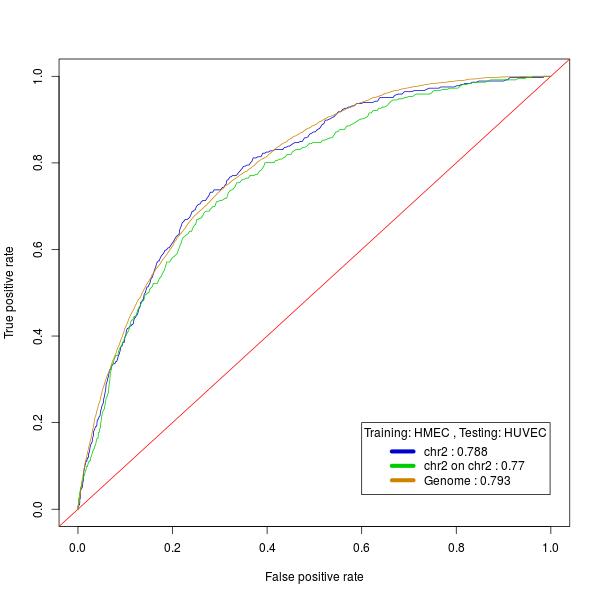 | 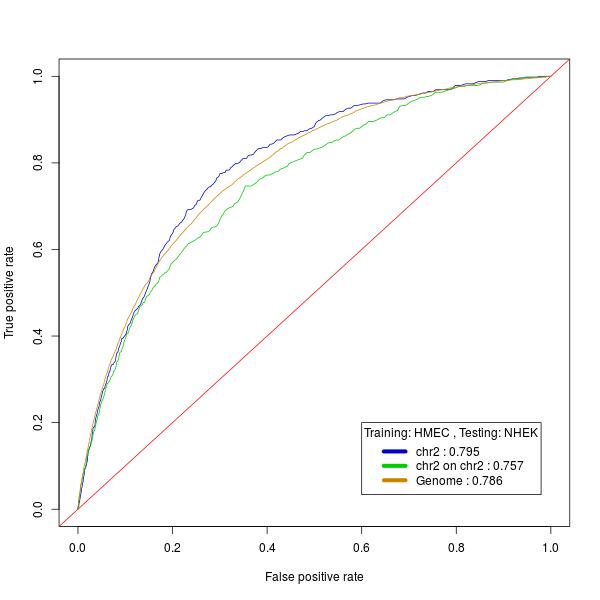 |
| 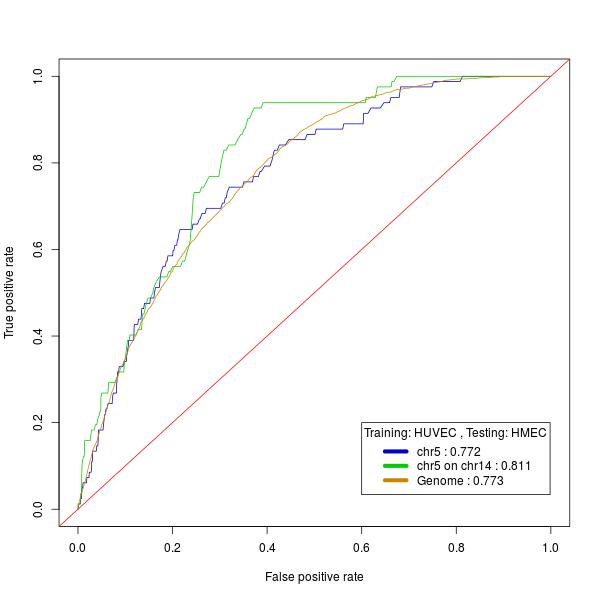 | 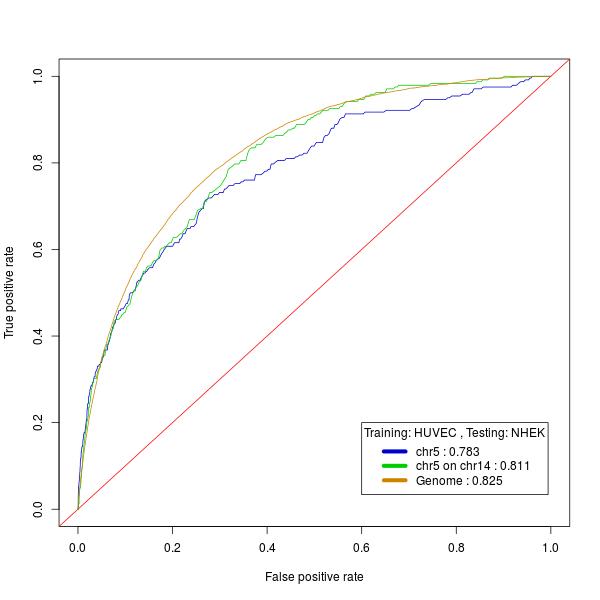 |
| 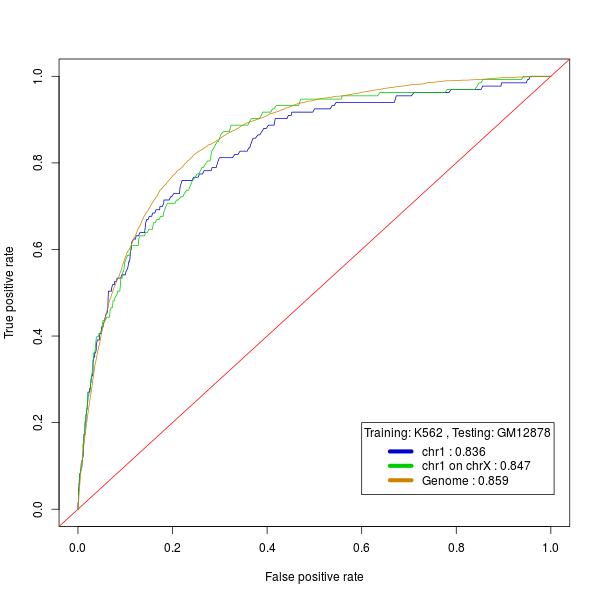 | 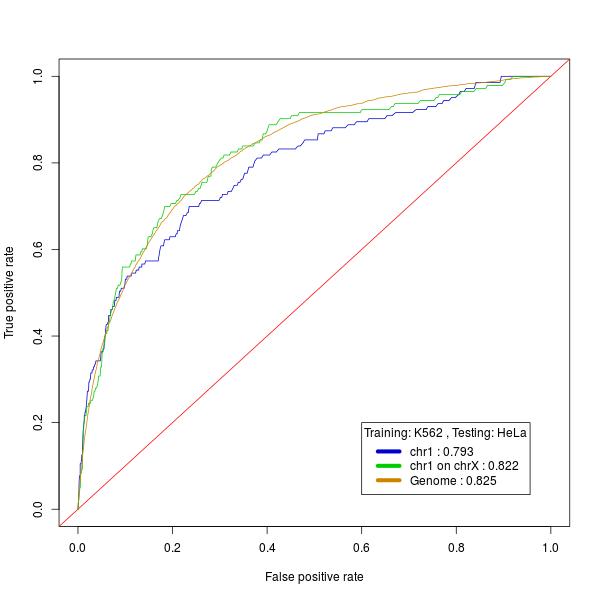 |
| 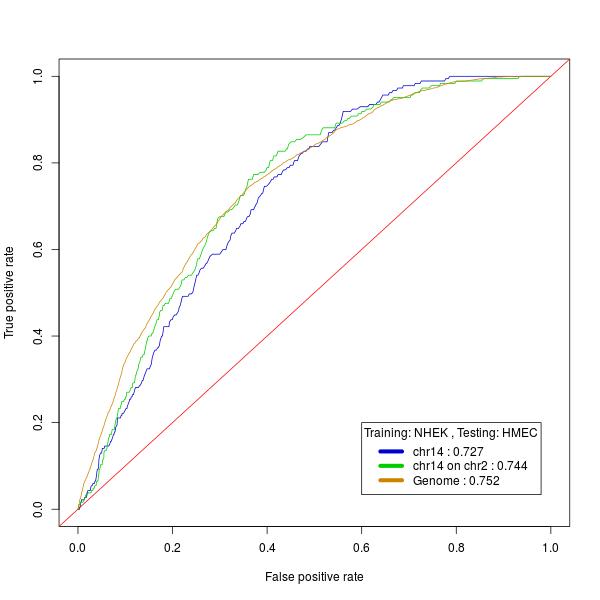 | 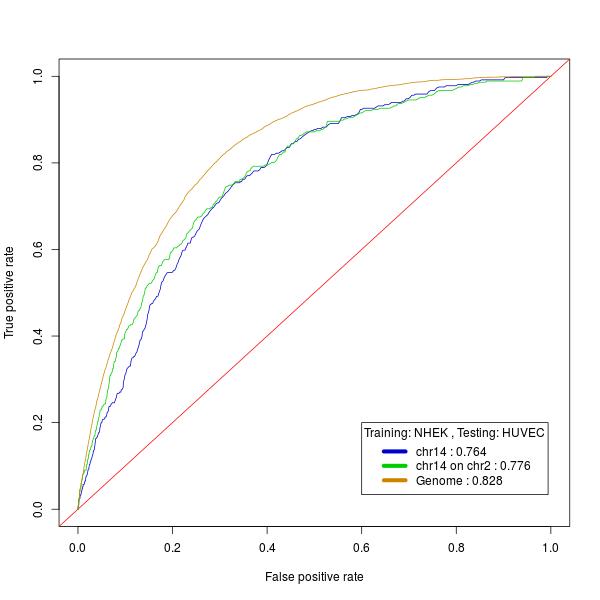 |

Figure 15. Genome levels predictors ROC Curves. Each Curve represent the performance predictor trained on cell type, and tested on another cell type at different genomics scales: the whole genome in orange, trained on chromosome and tested on another chromosome in green, and trained and tested on the same chromosome in blue.

## Comparison with EpiTensor

We compared our *in situ* Hi-C loops prediction with EpiTensor results. We collected IMR90 interactions predicted by EpiTensor, and compared them with IMR90 interactions identified by our genome level predictor trained on HeLa cell type. The reported Hi-C loops contain 8040 pairs, 4343 of them are experimental target set, i.e. they have anchors belong to the studied TADs and contain CTCF peaks. EpiTensor predict 105799 interactions, only 746 of them were confirmed by target set. Our HeLa-trained predictor identify 92474 pairs in total, 3359 pairs of them were confirmed by the target interactions set. Venn diagram Figure 16 illustrates the intersection between the predicted interactions and the target interactions.


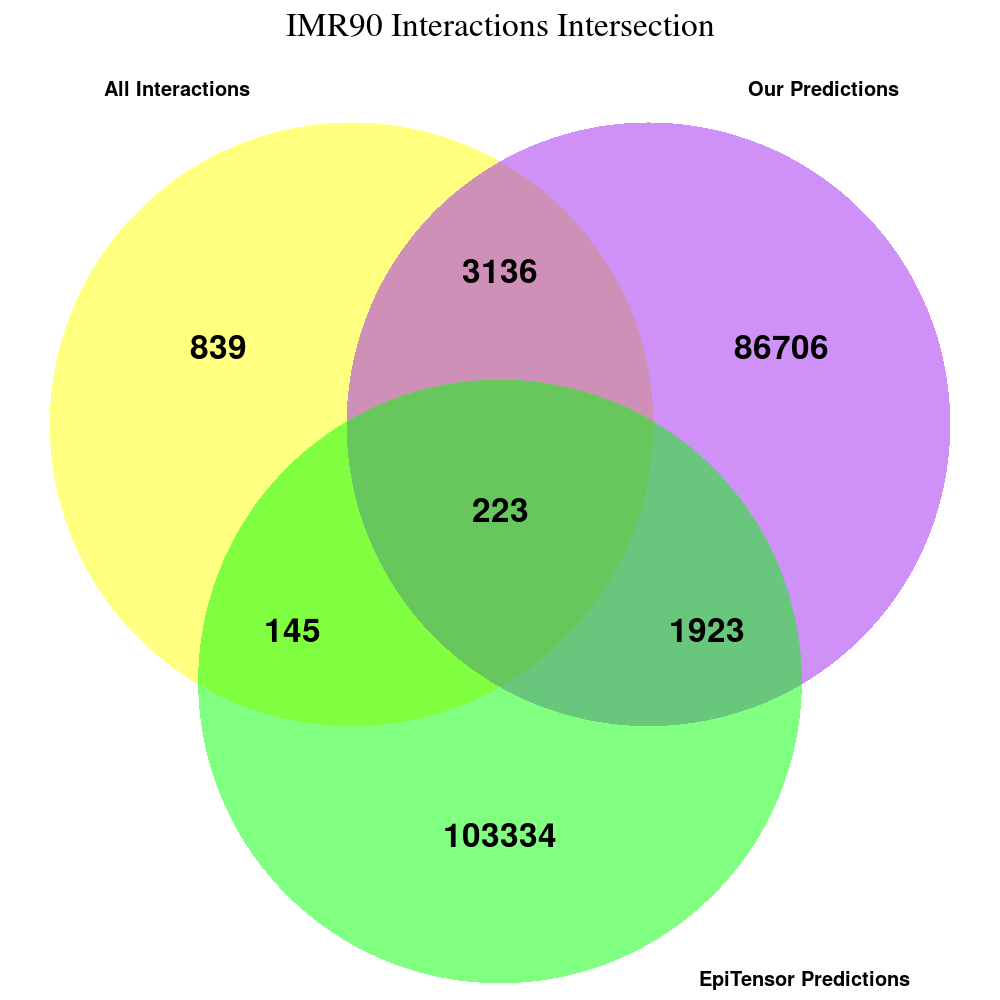


Figure 16. Hi-C loop comparison with EpiTensor. Our prediction in purple, the experimental interactions are in yellow, and EPiTensor interactions are in green.

## Comparison of predicted chromatin loops with ChIA-PET and in situ Hi-C experiments

For further verification, we predicted the physical interactions for different cell types, using genome level predictor trained on ChIA-PET physical CTCF-mediated interactions of other cell types. The results where verified with *long-range* ChIA-PET interactions and *in situ* Hi-C chromatin loops. GM12878 interactions were predicted using K562 genome predictor, and vice versa: K562 interactions were predicted by GM12878 genome level predictor. The HeLa interactions were identified using a predictor trained on MCF7 cell types. For further analysis we trained the genome level predictor using Hi-C loops then compared the predictions when applying on GM12878, K562, and HeLa cell lines on their CTCF ChIA-PET experimental interactions. We found that ~ 71 % of CTCF ChIA-PET interactions were also reported in Hi-C loops trained predictor. For GM12878 cell line: the predictor trained on K562 Hi-C loops and returned 101139 interaction. 21935 of them were reported experimentally in a data contains 30223 interactions. For K562 cell line: the predictor trained on HeLa Hi-C loops and returned 111998 interaction. 19130 of them were reported experimentally in a data contains 27093 interactions. For HeLa cell line: the predictor trained on K562 Hi-C loops and returned 153309 interaction. 12656 of them were reported experimentally in a data contains K562 on HeLa: 17744 interactions.

On Figure 17 Venn diagrams illustrates the intersections of the verified predictions for those cell lines.

| 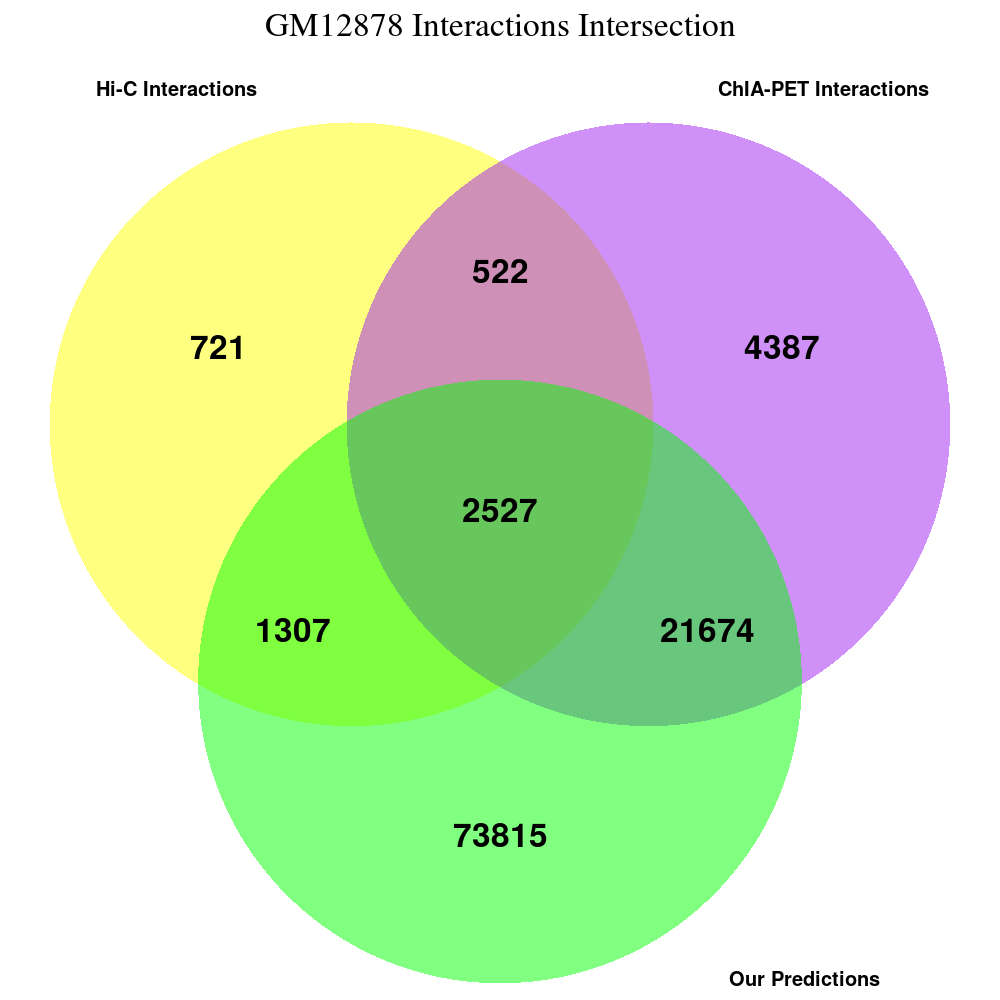 | 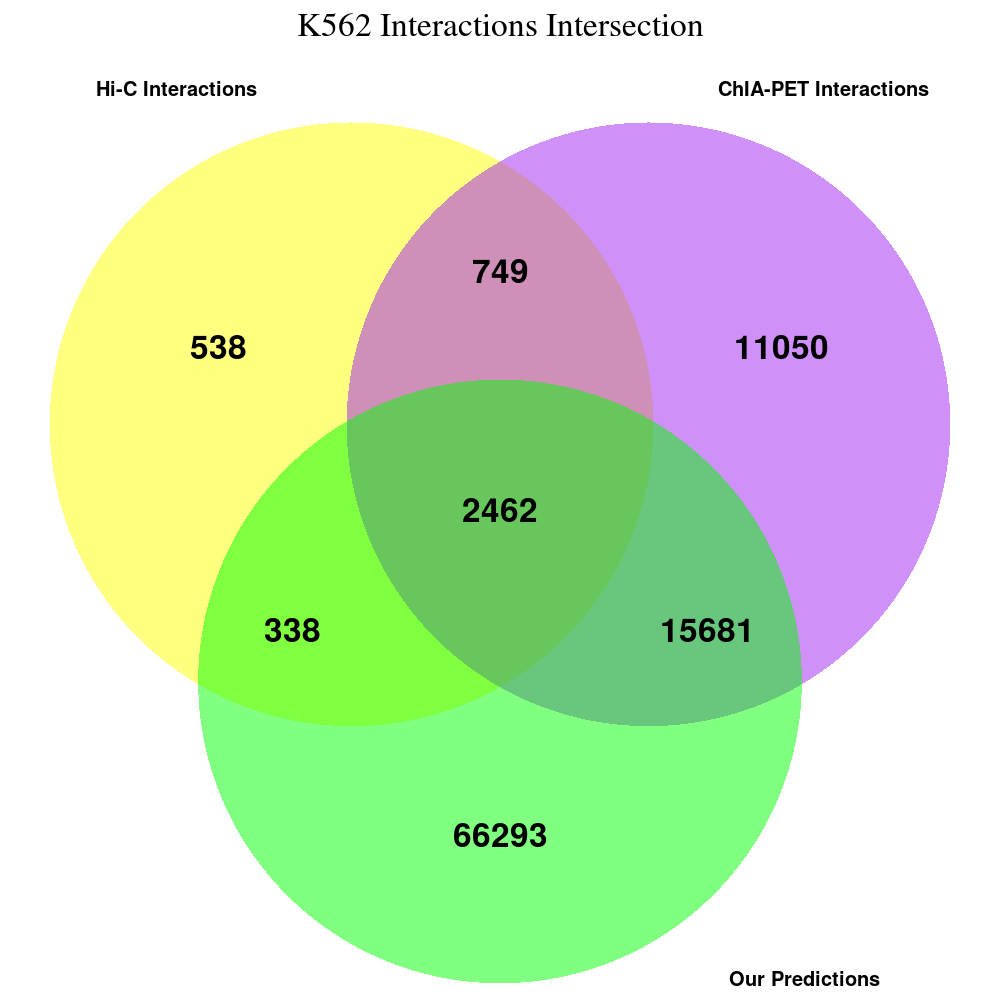 |
| --- | --- |


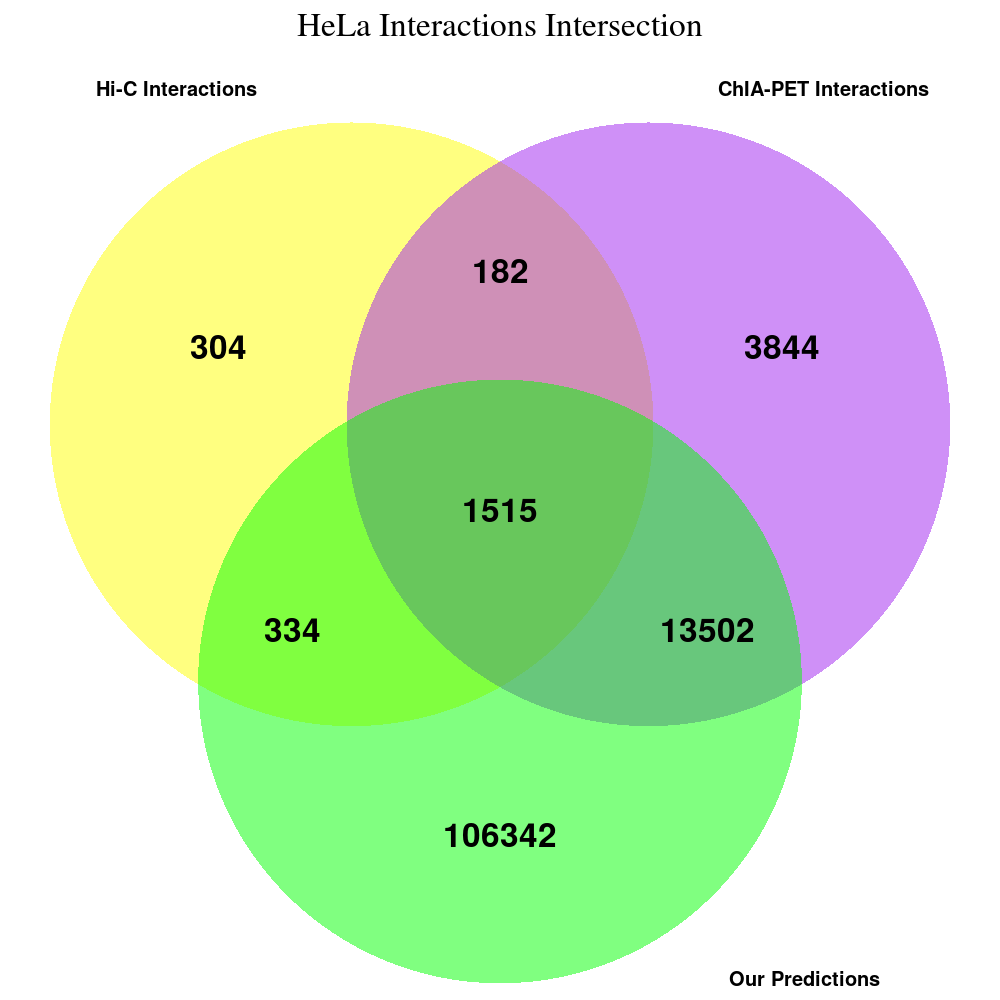


Figure 17 Venn diagram represents the intersections between our predicted CTCF ChIA-PET Interactions and experimental CTCF Chia-PET and Hi-C Loops. Our predictions in green, experimental CTCF are in purple, Hi-C loops in yellow

# RNAP II ChIA-PET Interactions

In the next phase of our study we applied our supervised method to predict functional interaction mediated by RNAP II protein. Four cell lines were analyzed, three human: GM12878, K562, HeLa, and one mouse: CH12.

## Input Features

**Transcription Factors:** CTCF, RNAP II, RAD21, and SMC3.

**Histone Modifications:** H3K4me1, H3K4me2, H3K4me3, H3K9ac, H3K9me3, H3K27ac, H3K27me3, H3K36me3, and H3K79me2.

See (Supplementary Table 1.xlsx) for full details of all features.

## Filtration process

The same filtration process as in CTCF Interactions prediction was applied here. The only difference was the selection of different key-features set. Transcription factor signals in the case of CTCF-mediated interactions were strong indicators for the interacting segments, while for RNAP II mediated interactions the histone modification signal became more important. We used the epigenomic profiles (H3K27ac, H3K4me1, H3K4me2, H3K9ac), and RNAP II binding profile to score the genomic segments. Than we applied the same steps as for CTCF ChIA-PET interactions filtration procedure. See the supplementary (Supplementary Table 2.xlsx) for filtration results.

## Applied Tests

All tests in the chromosome and genome level predictors were done similarly to CTCF ChIA-PET and *in situ* Hi-C chromatin loops identification and summarized in the supplementary file (Supplementary Table 5.xlsx).

The ROC curves obtained when applying genome predictor on other cell lines are illustrated in Figure 18 and Figure 19.

| 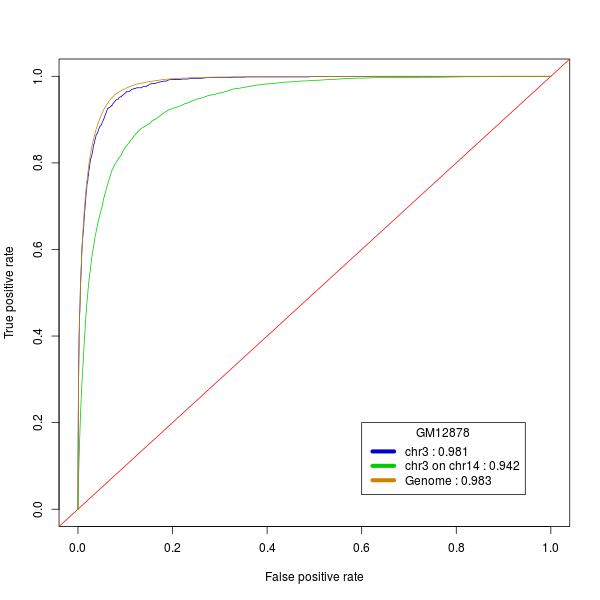 | 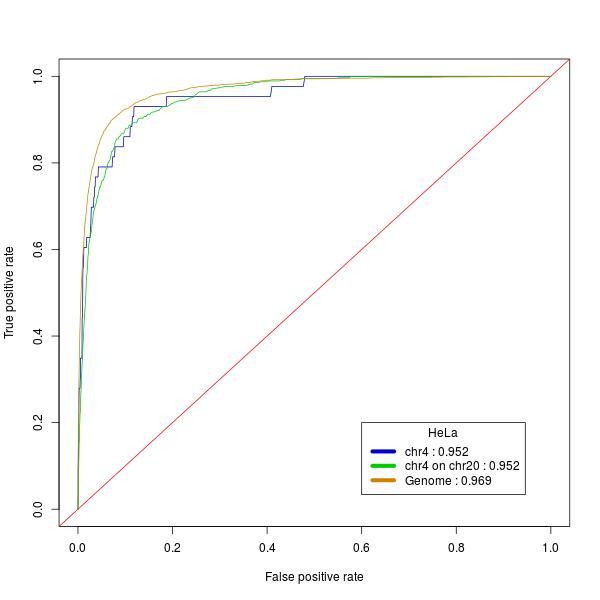 |
| --- | --- |
| 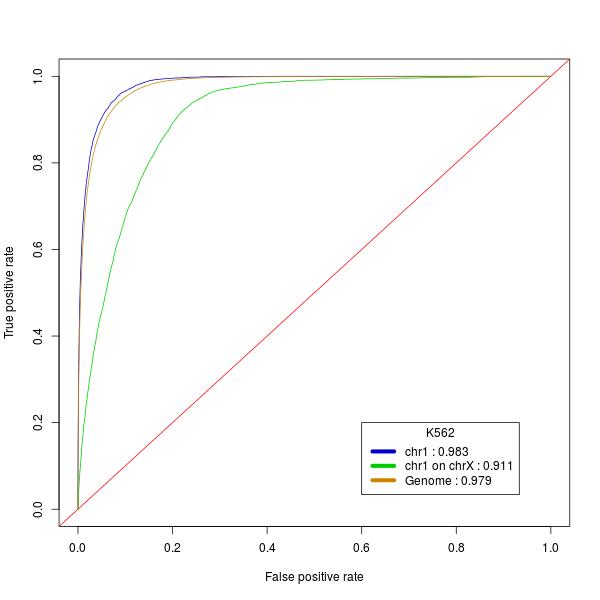 | 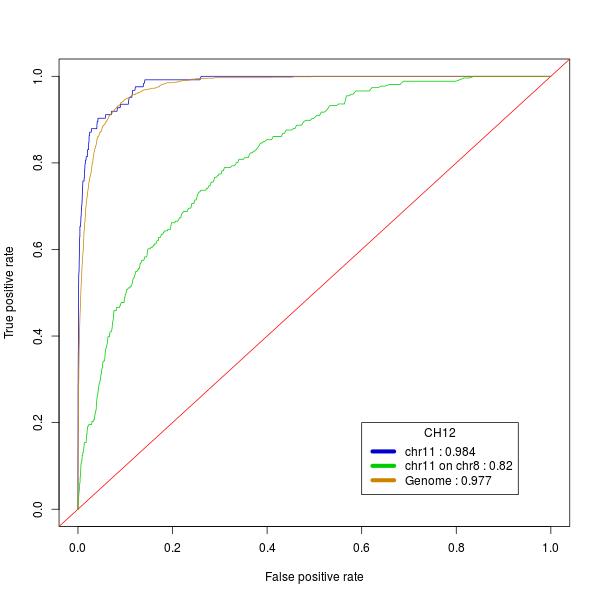 |

Figure 18 ROC Curves obtained for the predictors trained on GM12878, K562, HeLa, and CH12 cell lines. Three curves for each cell line representing different genomics scales: the whole genome in orange, trained on chromosome and tested on another chromosome in green, and trained and tested on the same chromosome in blue.

| 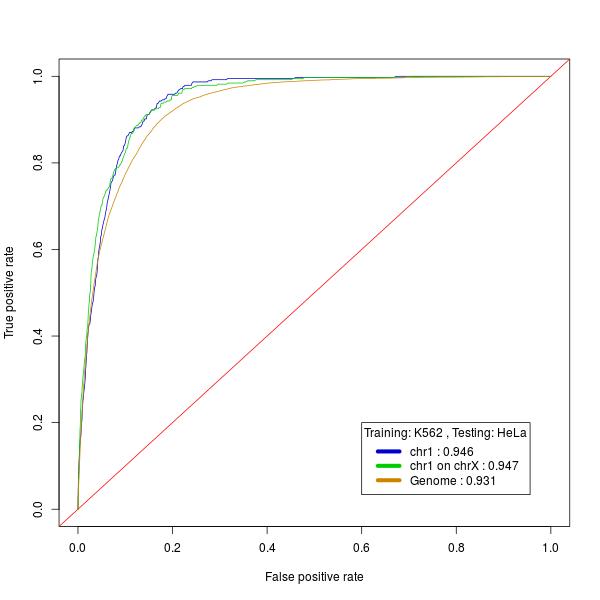 | 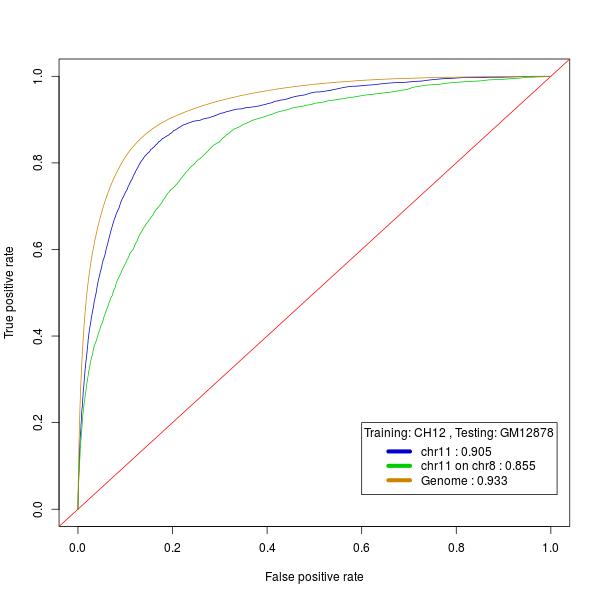 |
| --- | --- |
| 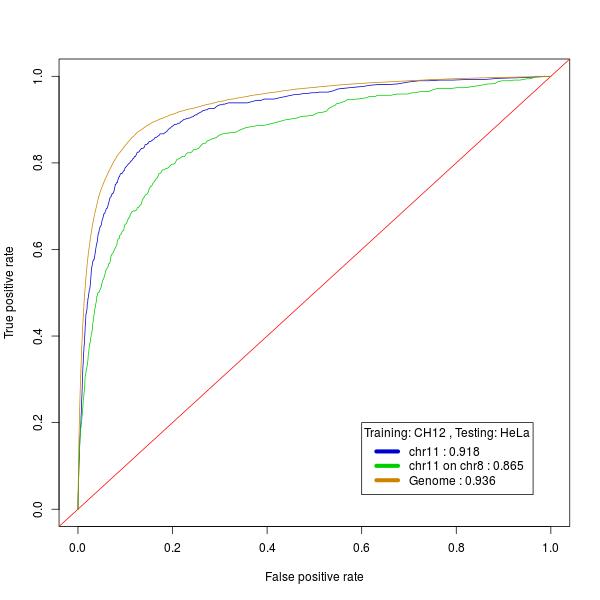 | 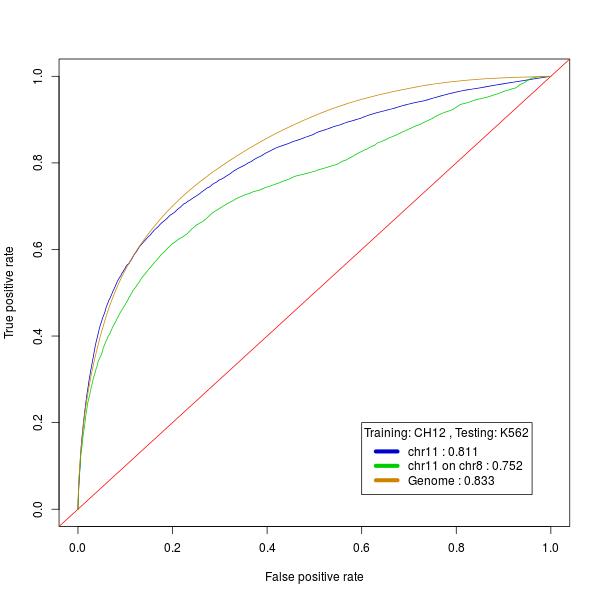 |
| 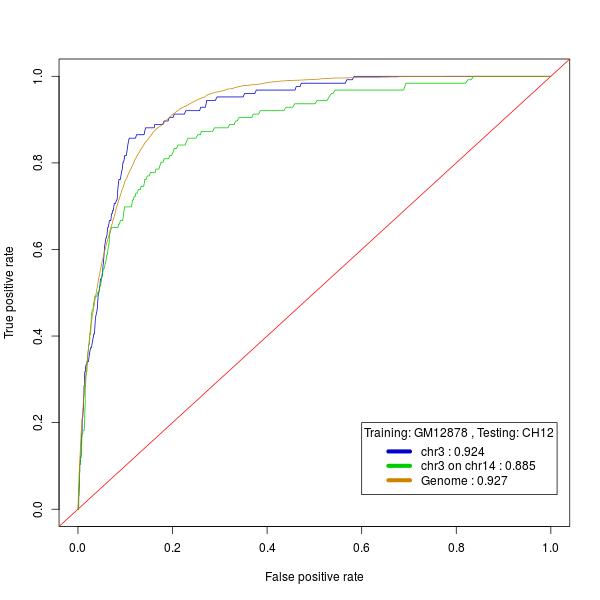 | 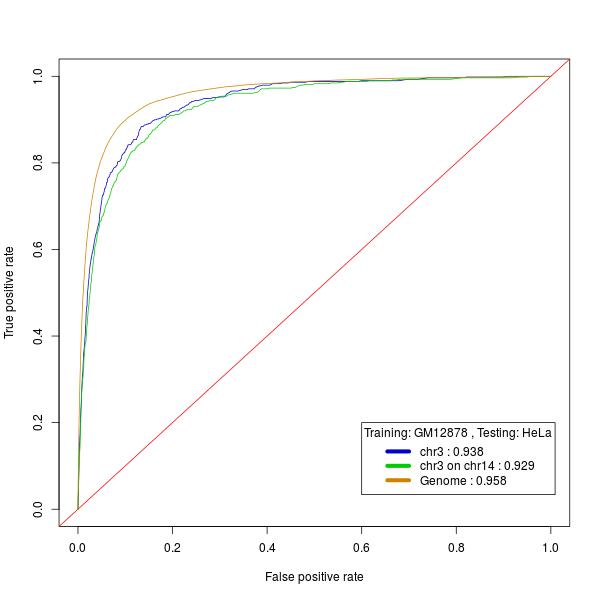 |
| 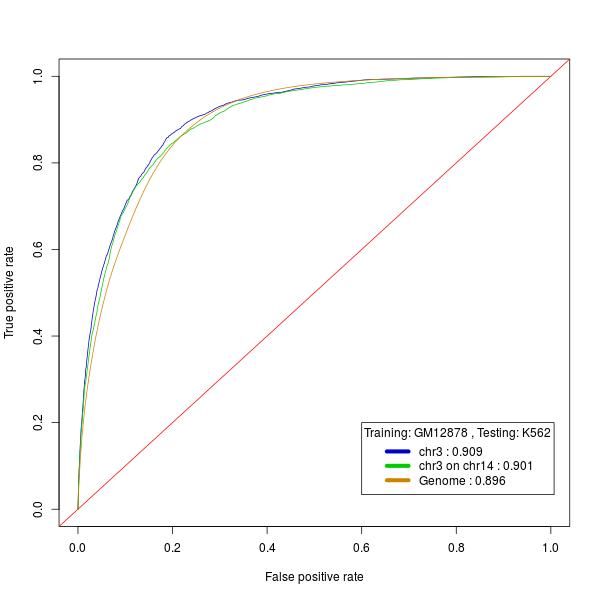 | 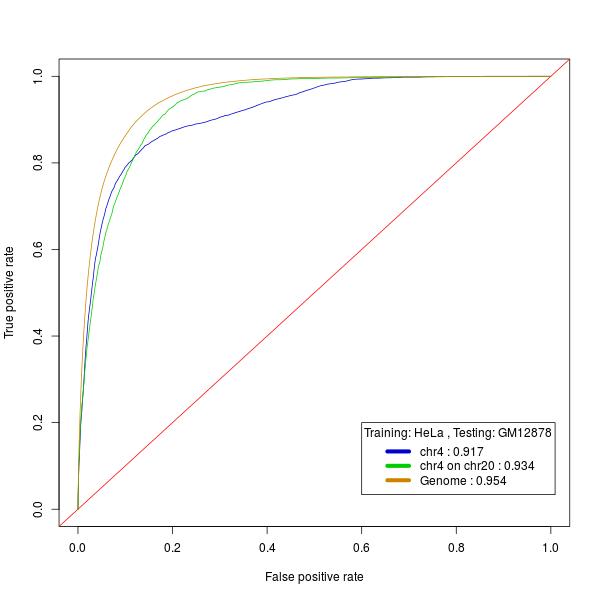 |
| 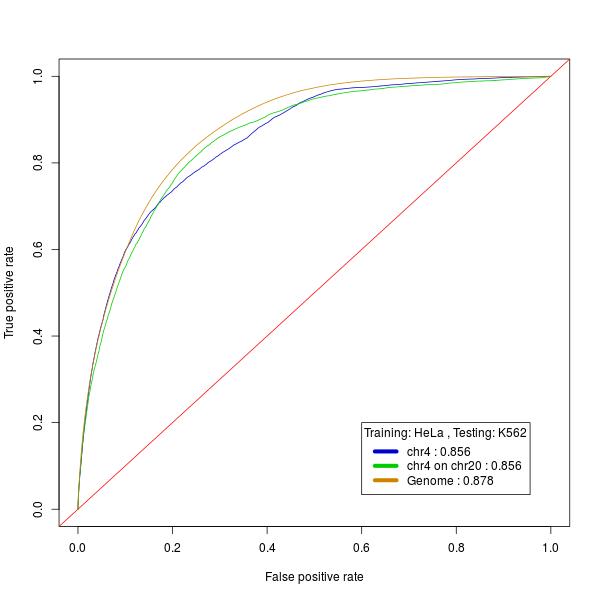 | 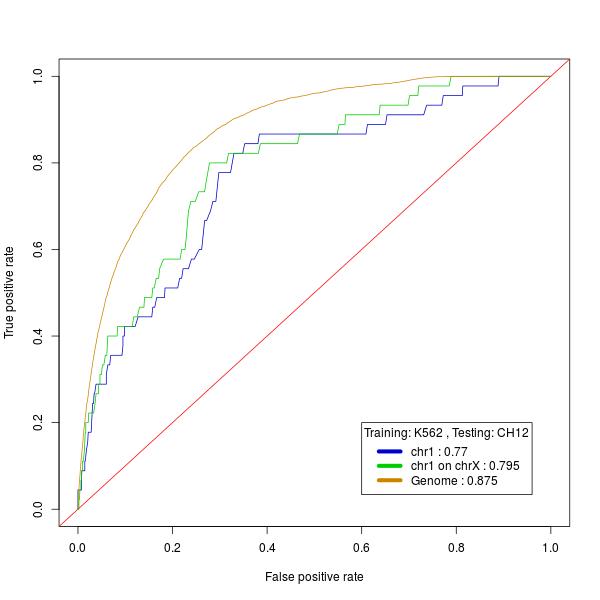 |
| 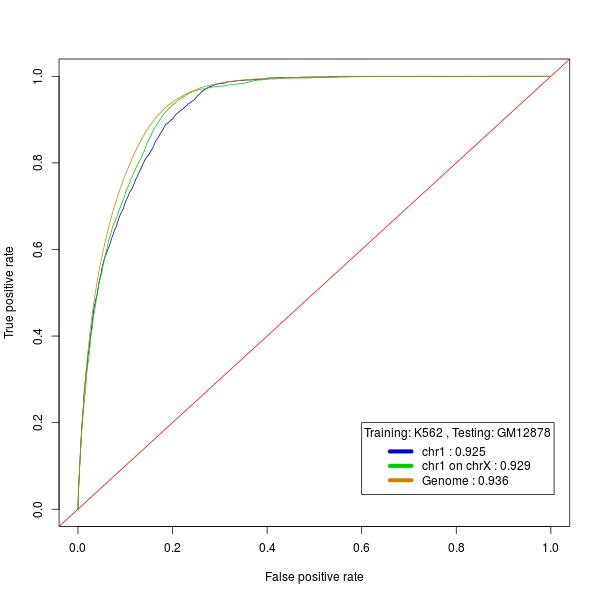 | |

Figure 19 ROC Curves obtained for the predictors trained on cell line and tested on another cell line. Three curves for each cell line representing different genomics scales: the whole genome in orange, trained on chromosome and tested on another chromosome in green, and trained and tested on the same chromosome in blue

## Important Features

Here we report the important features for RNAP II interactions predictor at the whole genome scale according to the Random Forest (RF) classifier.

| 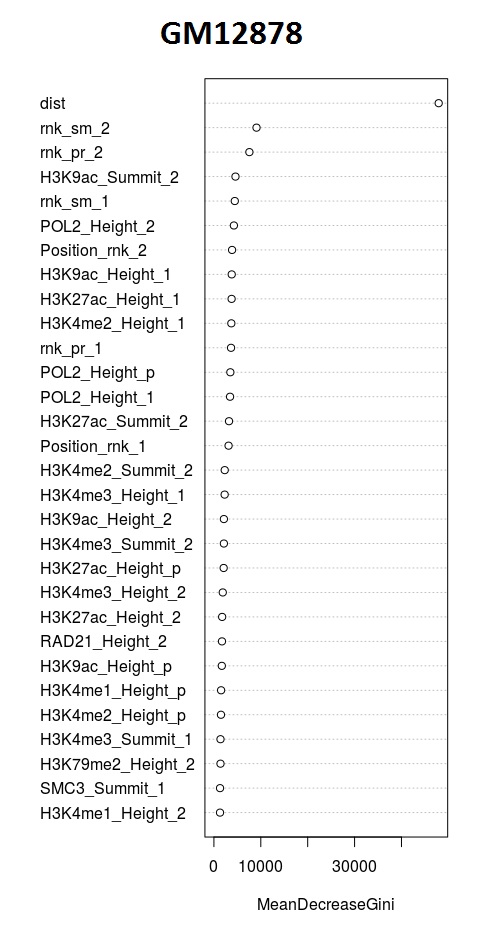 | 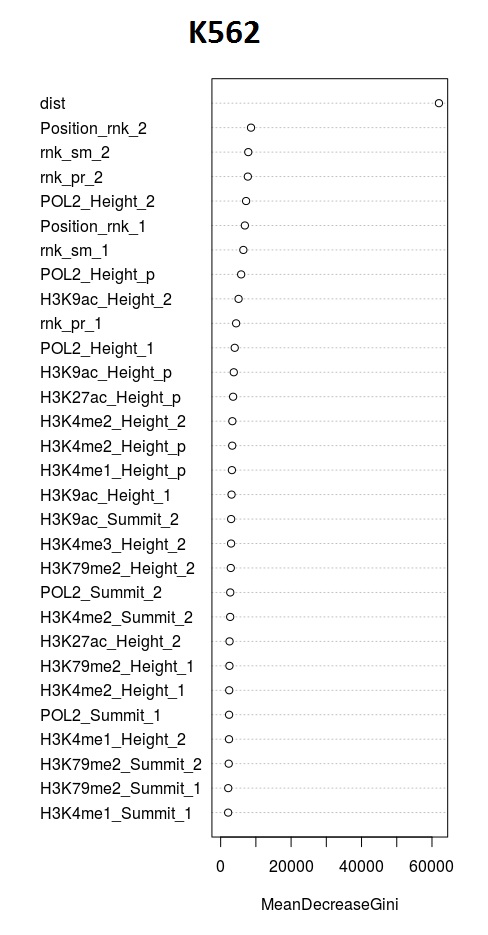 |
| --- | --- |
| 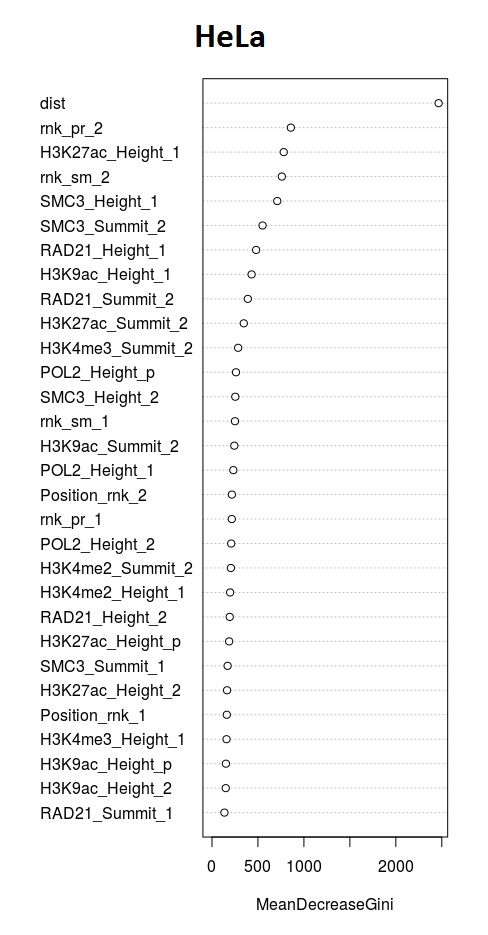 | 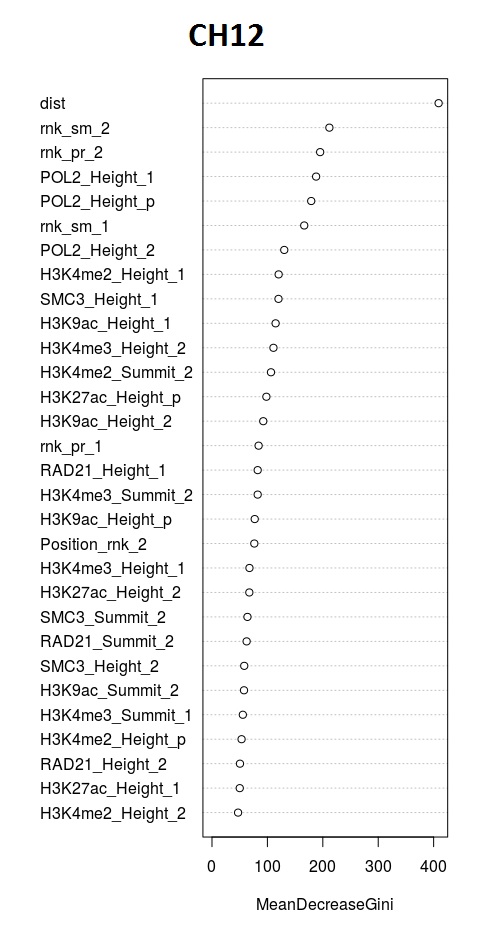 |

Figure 20 the important features according to RF classifier for the cell lines: GM12878, K562, HeLa and mouse cell line CH12.

## Comparison with EpiTensor

We also compared predictions of our supervised method with EpiTensor unsupervised algorithm for all cell lines. The results are presented on Figure 21.


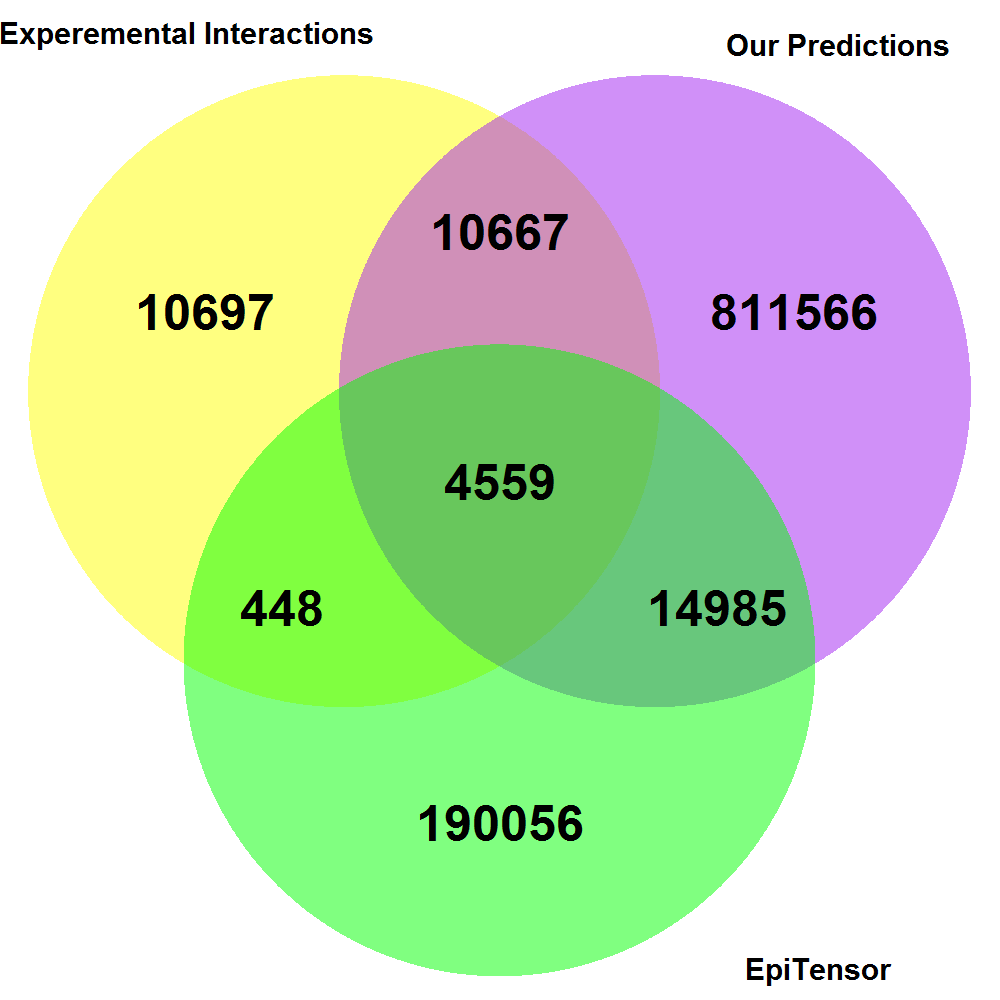


Figure 21 Venn diagram: the intersections of our predicted RNAP II interactions (in purple) with the experimental interactions (in Yellow) and EpiTensor interactions (in Green)

# *In situ* Hi-C Interactions Prediction

## Training and testing datasets selection

The training of supervised method for *in situ* Hi-C heatmaps is methodologically more challenging comparing with physical interactions from ChIA-PET, or chromatin loops. Hi-C experiments results are affected by noise and polymer physico-chemical effects. Interactions with the high score on heatmaps does not reflect the true importance of the interactions, i.e. whether they are physical ones, or only reflecting some spatial proximity. The majority of strong *in situ* Hi-C heatmap-based interactions are characterized by the short distance between the anchors. The heatmap plot represents the relation between the genomic distance between the anchors and the strength of the interaction by the dark pixels close to the diagonal (where the anchors are close to each other). The Figure 22 illustrates the density distribution of the normalized scores of the heatmaps according to the genomic distance between the anchors. Almost all segments within the same TAD are interacting, but they vary in the strength of the interaction especially when using low resolution data. Predicting the strongest *in situ* Hi-C interactions within the TADs turned out to be the search procedure for the closest pairs identification. In order to define the important interactions for each TAD, we took the genomic distance into the consideration. We identified the importance of the interaction as the p-value of the normalized score when comparing with the normalized score of the interactions sharing the same genomic distance between their anchors. In other words, the strength of any interaction is relative to all other interactions sharing the same genomic distance between their anchors. We calculated the p-values of the interactions as follows:

1. We grouped the pairs based on their genome distance (each group contains all pairs with the same genomic distance between the anchors).
2. For each group, we ranked its pairs using the normalized scores obtained from the experimental heatmaps, and finally divided the rank on the pairs count:

$pvalue=\frac{{rank}_{normalized score}}{pairs count}$.

1. The separation between the important and un-important interactions was done by using the optimal threshold for the p-values.
2. The optimal threshold should achieve the high coverage of the physical interactions (Hi-C chromatin loops and ChIA-PET interactions) and doesn't affect the performance of our predictors.
3. The pairs are classified either true interactions, when their p-values are less than the optimal chosen threshold, or false interactions when their p-values are greater than (1 – threshold).
4. We investigated three different options for the optimal threshold, then we chose 0.15. See the applied tests 5.2.2 for full details.


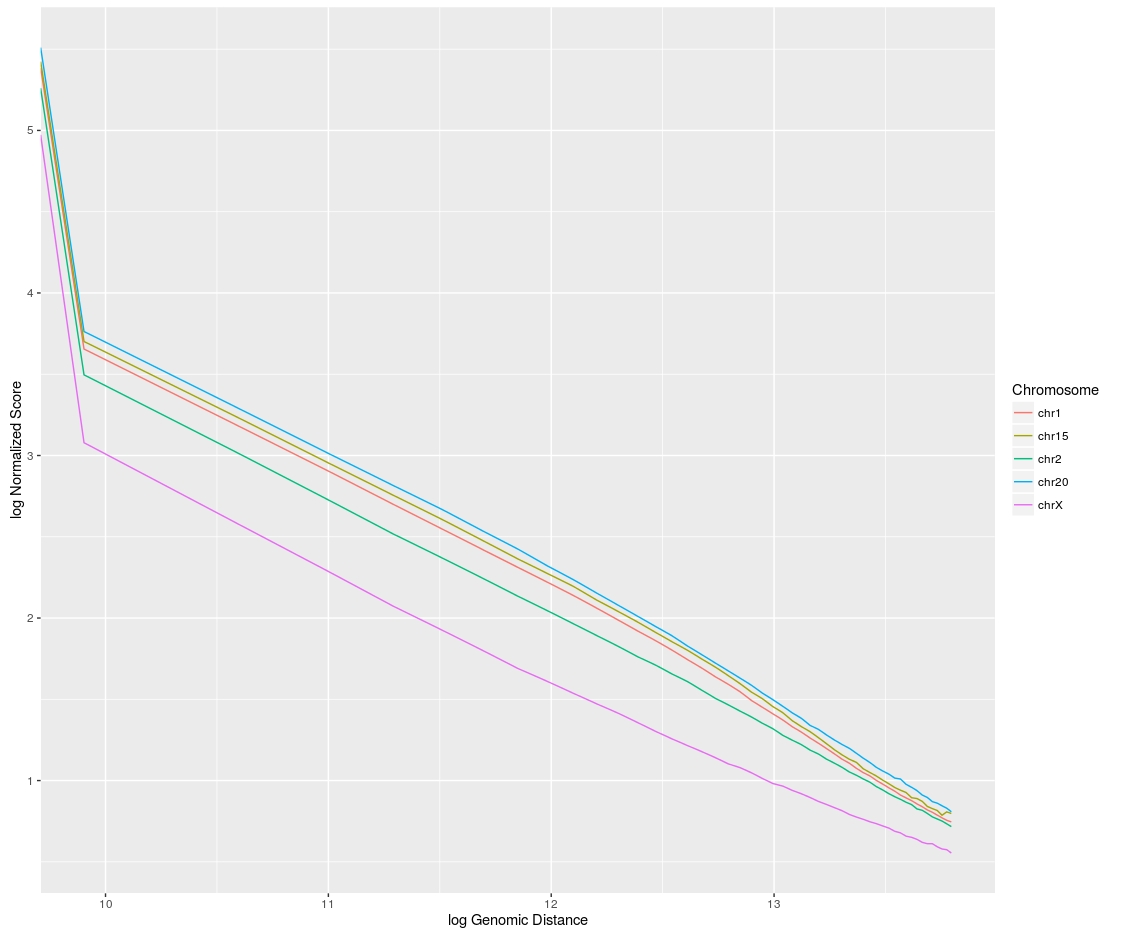


Figure 22. The log-log plot of the normalized scores according to the genomic distance between the anchors

## GM12878 analysis with All Features

### Input Features in this test

**Transcription factors**: ATF2, ATF3, BATF, BCL11, BCL3, BCLAF, BHLHE40, BRCA1, CDP, CEBPB, CHD1, CHD2, cMYC, COREST, CREB1, CTCF, E2F4, EBF1, EBF, EGR1, ELF1, ELK1, ERRA, ETS1, EZH2, FOXM1, GABP, GCN5, IRF3, IRF4, JUND, MEF2A, MEF2C, MTA3, NFATC1, NFE2, NFIC, NRF1, NRSF, P300, PAX5,PBX3, PML, POL2-4H8, POL2-HAIB, POL2, POL2-S2P, POU2F2, PU.1, RAD21, RAD21-SNYDER, RFX5, RUNX3, RXRA, SA1, SIX5, SMC3, SP1, SPT20, SRF, STAT1, STAT3, STAT5, TAF1, TBP, TCF12, TCF3, USF1, USF2, WHIP, YY1, ZBTB33, ZEB1, and ZNF143.

**Histone Modifications**: H2AFZ, H3K27ac, H3K27me3, H3K36me3, H3K4me1, H3K4me2, H3K4me3, H3K79me2, H3K9ac, H3K9me1, H3K9me3, and H4K20me1.

See Supplementary Table 1.xlsx for full details of all features and their resources.

### Applied Test

In the first test, we tried to choose the optimal threshold to separate the important interactions from unimportant ones, we analyzed three different threshold values (0.15, 0.25, 0.5), choosing the optimal one according to the coverage of the chromatin loops without scarifying the performance of our predictors. The Table 10 present the physical interactions coverage obtained when using different threshold values. The pairs are classified either as true interactions, when their p-values are less than the optimal chosen threshold, or false interactions when their p-values are greater than (1 - threshold). The pairs were split into training dataset (80%) and testing dataset (20%). About 2100 different TADs were analyzed in the same way using three different thresholds. Two predictors were built for each genomic domain: the first using all features, the second without the genomic distance. This will allow us to assess the influence of the genomic distance on supervised learning algorithm.

GM12878 cell line has a lot of published Epigenomic profiles and transcription factors binding assays, in addition to *in situ* Hi-C heatmaps (5 kb and 40 kb resolution), Hi-C chromatin loops and ChIA-PET interactions. Therefore, we analyzed and selected the best threshold value for this cell line. The genome was divided into 5 kb resolution segments, no segments filtration was applied in this case. The pairs were formed from all possible segments combinations within the same TAD. We compare those pairs with *in situ* Hi-C heatmaps with 5 kb resolution, Hi-C chromatin loops, and ChIA-PET CTCF-mediated interactions. True interactions (what we want to predict) are the interaction with normalized score less than the selected threshold. False interactions are the interactions with normalized score greater than (1 – selected threshold). See the supplementary file (Supplementary Table 6.xlsx) for the detailed description of the percentage of the covered physical interaction within the true interactions according to each threshold.

| Threshold | All Hi-C | Covered Hi-C Loops | Covered Hi-C in True Interactions | All ChIA-PET | Covered ChIA-PET | Covered ChIA-PET in True Interactions |
| --- | --- | --- | --- | --- | --- | --- |
| 0.15 | 11944 | 11145 (93%) | 11128 (93.1%) | 65489 | 44695 (68%) | 42918 (65%) |
| 0.25 | 11944 | 11562 (96%) | 11194 (93.7%) | 65489 | 52275 (79%) | 43758 (66%) |
| 0.5 | 11944 | 11944 (100%) | 11194 (93.7%) | 65489 | 65489 (100%) | 43758 (66%) |

Table 10 Physical Interactions (Hi-C loops, and CTCF ChIA-PET) Coverage when using different thresholds. Columns [3, 6] represent the covered physical interactions in the filtered interactions according to the specified threshold. Columns [4, 7] represent the covered physical interactions in the filtered interactions which have p-value less than the specified threshold.

We can see from the table that both threshold 0.25, and 0.5 cover the same percentage of the physical interactions, and some of the physical interactions are not covered by the true interactions because they have low score in the heatmaps.

We built one predictor for each TAD, the full results of these tests are described in the supplementary file (Supplementary Table 6.xlsx). On Figure 23 we illustrate the heatmaps after applying the filtration process using three different threshold (0.15, 0.25, and 0.5).

|  | Full Heatmap | Training Heatmap | Testing Heatamp |
| --- | --- | --- | --- |
| 0.15 | 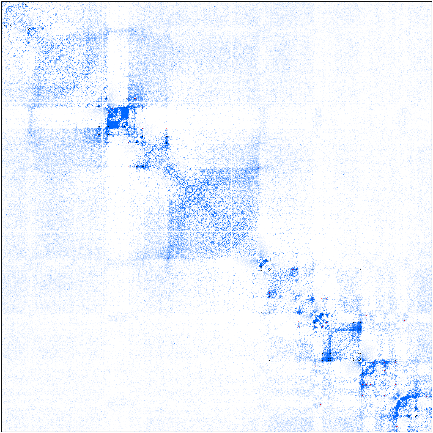 | 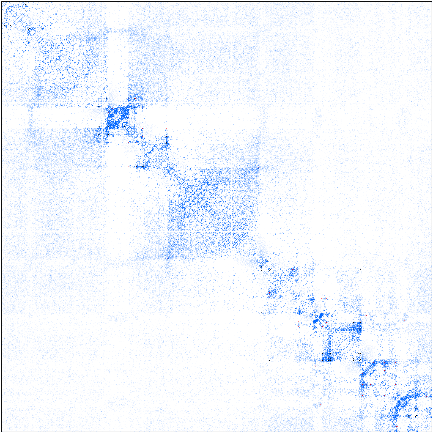 | 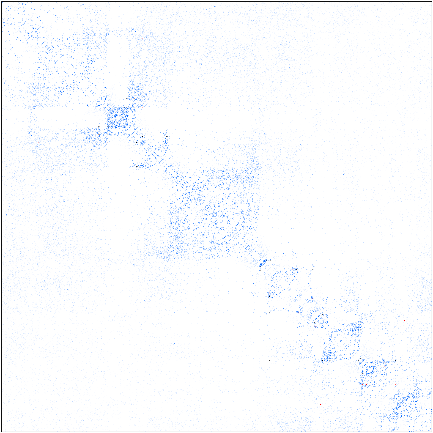 |
| 0.25 | 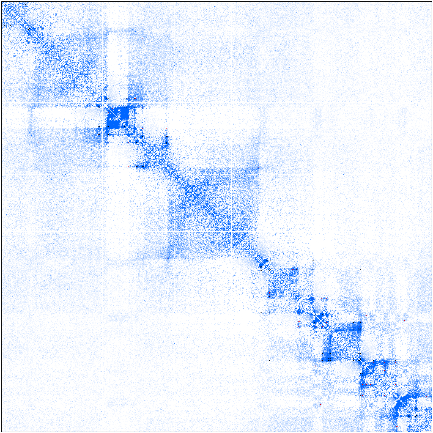 | 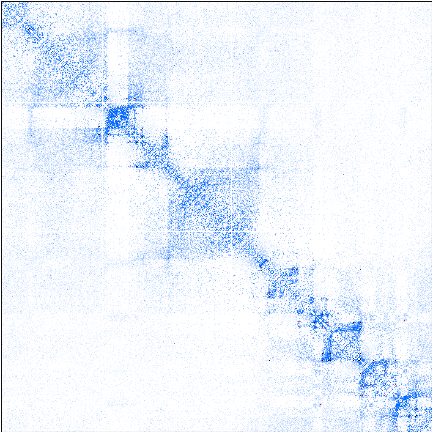 | 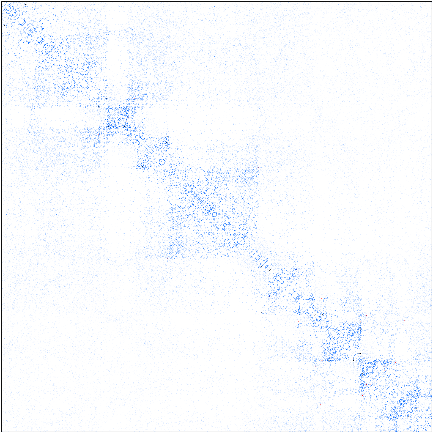 |
| 0.5 | 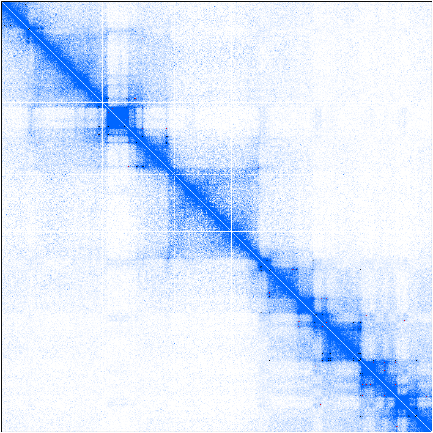 | 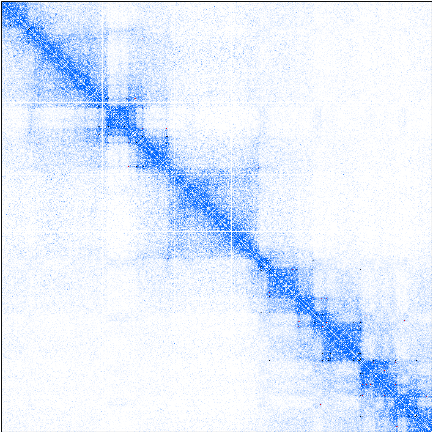 | 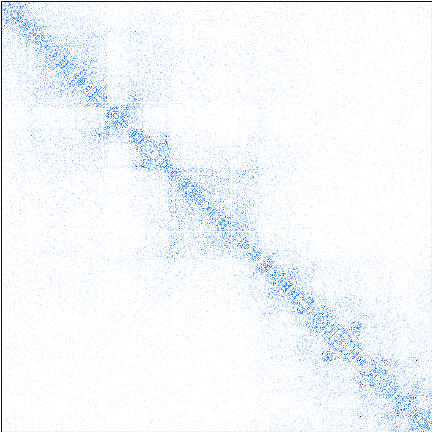 |

Figure 23 GM12878 original, Training, and testing Heatmaps after the filtration according to the tested three thresholds (0.15, 0.25, and 0.25).

The full tests are available in the Supplementary Table 6.xlsx, but on average the predictors achieved the following measurements in the Table 11.

| *Threshold* | *accuracy* | *specificity* | *precision* | *Sensitivity* | *AUC* |
| --- | --- | --- | --- | --- | --- |
| 0.15 | 0.70 | 0.72 | 0.59 | 0.67 | 0.74 |
| 0.25 | 0.68 | 0.69 | 0.59 | 0.67 | 0.73 |
| 0.5 | 0.62 | 0.63 | 0.55 | 0.62 | 0.66 |

Table 11 Predictors Evaluation when using different thresholds.

At the end of this test we chose 0.15 as the optimal threshold, as it preserves most of the physical interactions, and slightly affects the performance of the predictor.

### Important Features

The important features according to TADs predictors are represent in the table below, showing the count of each feature in the specified ranking of the reported important features. The first row in the ranking 1 means that the distance got the ranking one over the important features in 2078 tests (one test for each TAD).

| RANKING:1 | |  | RANKING: 2 | |
| --- | --- | --- | --- | --- |
| distance | 2078 |  | H3K27ME3_SUMMIT_1 | 583 |
| H3K36ME3_SUMMIT_1 | 9 |  | H3K36ME3_SUMMIT_1 | 308 |
| H3K4ME1_HEIGHT_1 | 8 |  | H3K27ME3_HEIGHT_2 | 94 |
| H3K27ME3_SUMMIT_1 | 4 |  | H3K4ME1_HEIGHT_1 | 91 |
| H3K36ME3_HEIGHT_2 | 4 |  | H3K36ME3_HEIGHT_2 | 85 |
| H3K36ME3_SUMMIT_2 | 4 |  | PU.1_SUMMIT_1 | 67 |
| H3K79ME2_HEIGHT_1 | 4 |  | H2AZ_SUMMIT_1 | 57 |
| H3K27ME3_HEIGHT_2 | 3 |  | H3K4ME1_SUMMIT_1 | 55 |
| H3K36ME3_HEIGHT_1 | 3 |  | H3K36ME3_SUMMIT_2 | 48 |
| H3K79ME2_SUMMIT_2 | 3 |  | H3K79ME2_SUMMIT_1 | 40 |
| RANKING: 3 | |  | RANKING: 4 | |
| H3K27ME3_HEIGHT_2 | 416 |  | H3K36ME3_SUMMIT_1 | 193 |
| H3K36ME3_HEIGHT_2 | 209 |  | H3K27ME3_SUMMIT_1 | 155 |
| H3K27ME3_SUMMIT_1 | 163 |  | H3K4ME1_SUMMIT_1 | 134 |
| H3K36ME3_SUMMIT_1 | 153 |  | H3K27ME3_HEIGHT_2 | 119 |
| H3K4ME1_SUMMIT_1 | 75 |  | H3K36ME3_HEIGHT_2 | 116 |
| PU.1_SUMMIT_1 | 65 |  | H3K4ME1_HEIGHT_2 | 92 |
| H3K4ME1_SUMMIT_2 | 63 |  | H3K4ME1_HEIGHT_1 | 78 |
| H3K4ME1_HEIGHT_1 | 57 |  | PU.1_SUMMIT_1 | 73 |
| H3K4ME1_HEIGHT_2 | 51 |  | PU.1_HEIGHT_2 | 66 |
| PU.1_HEIGHT_2 | 51 |  | H3K27ME3_SUMMIT_2 | 53 |
| RANKING: 5 | |  | RANKING: 6 | |
| H3K36ME3_HEIGHT_2 | 163 |  | H3K4ME1_SUMMIT_1 | 194 |
| H3K4ME1_SUMMIT_1 | 144 |  | H3K4ME1_HEIGHT_2 | 118 |
| H3K27ME3_HEIGHT_2 | 138 |  | H3K36ME3_SUMMIT_1 | 91 |
| H3K4ME1_HEIGHT_2 | 107 |  | H3K36ME3_HEIGHT_2 | 82 |
| H3K36ME3_SUMMIT_1 | 91 |  | H3K27ME3_SUMMIT_1 | 76 |
| H3K27ME3_SUMMIT_1 | 82 |  | PU.1_HEIGHT_2 | 76 |
| PU.1_SUMMIT_1 | 82 |  | H3K27ME3_HEIGHT_2 | 74 |
| PU.1_HEIGHT_2 | 79 |  | PU.1_SUMMIT_1 | 67 |
| H3K4ME1_SUMMIT_2 | 68 |  | H3K4ME1_SUMMIT_2 | 58 |
| H3K4ME1_HEIGHT_1 | 54 |  | H2AZ_SUMMIT_1 | 48 |
| RANKING: 7 | |  | RANKING: 8 | |
| H3K4ME1_SUMMIT_1 | 167 |  | H3K4ME1_SUMMIT_1 | 145 |
| H3K4ME1_HEIGHT_2 | 145 |  | H3K4ME1_HEIGHT_2 | 127 |
| H3K27ME3_HEIGHT_2 | 111 |  | H3K4ME1_HEIGHT_1 | 75 |
| H3K36ME3_HEIGHT_2 | 82 |  | PU.1_SUMMIT_1 | 74 |
| PU.1_SUMMIT_1 | 69 |  | PU.1_HEIGHT_2 | 65 |
| H3K4ME1_SUMMIT_2 | 65 |  | H3K36ME3_HEIGHT_2 | 57 |
| PU.1_HEIGHT_2 | 60 |  | H3K27ME3_HEIGHT_2 | 56 |
| H3K4ME1_HEIGHT_1 | 56 |  | H3K36ME3_SUMMIT_1 | 51 |
| H3K36ME3_SUMMIT_1 | 53 |  | H3K4ME1_SUMMIT_2 | 50 |
| H3K27ME3_SUMMIT_1 | 47 |  | RUNX3_SUMMIT_1 | 50 |
| RANKING: 9 | |  | RANKING: 10 | |
| H3K4ME1_HEIGHT_2 | 134 |  | H3K4ME1_HEIGHT_2 | 116 |
| H3K4ME1_SUMMIT_1 | 85 |  | H3K4ME1_SUMMIT_1 | 102 |
| H3K36ME3_HEIGHT_2 | 73 |  | PU.1_HEIGHT_2 | 83 |
| PU.1_SUMMIT_1 | 71 |  | PU.1_SUMMIT_1 | 74 |
| PU.1_HEIGHT_2 | 67 |  | H3K4ME1_SUMMIT_2 | 56 |
| H3K27ME3_HEIGHT_2 | 63 |  | RUNX3_HEIGHT_2 | 53 |
| H3K4ME1_SUMMIT_2 | 60 |  | H2AZ_SUMMIT_1 | 50 |
| H3K4ME1_HEIGHT_1 | 51 |  | H3K79ME2_SUMMIT_1 | 49 |
| ELF1_HEIGHT_2 | 44 |  | POL2.4H8_SUMMIT_1 | 47 |
| H2AZ_SUMMIT_1 | 43 |  | POL2_SUMMIT_1 | 45 |
| RANKING: 11 | |  | RANKING: 12 | |
| H3K4ME1_HEIGHT_2 | 88 |  | H3K4ME1_HEIGHT_2 | 88 |
| H3K4ME1_SUMMIT_1 | 86 |  | H3K4ME1_SUMMIT_1 | 70 |
| PU.1_SUMMIT_1 | 77 |  | PU.1_SUMMIT_1 | 70 |
| PU.1_HEIGHT_2 | 75 |  | PU.1_HEIGHT_2 | 62 |
| H3K4ME1_HEIGHT_1 | 67 |  | RAD21_HEIGHT_2 | 59 |
| H3K4ME1_SUMMIT_2 | 60 |  | RUNX3_SUMMIT_1 | 53 |
| RUNX3_SUMMIT_1 | 50 |  | POL2.4H8_SUMMIT_1 | 52 |
| H2AZ_SUMMIT_1 | 48 |  | SA1_HEIGHT_2 | 51 |
| POL2.4H8_SUMMIT_1 | 47 |  | H2AZ_SUMMIT_1 | 46 |
| POL2_SUMMIT_1 | 42 |  | ELF1_SUMMIT_1 | 45 |
| RANKING: 13 | |  | RANKING: 14 | |
| H3K4ME1_HEIGHT_2 | 78 |  | RUNX3_SUMMIT_1 | 72 |
| H3K4ME1_SUMMIT_1 | 70 |  | PU.1_HEIGHT_2 | 69 |
| PU.1_SUMMIT_1 | 64 |  | PU.1_SUMMIT_1 | 67 |
| PU.1_HEIGHT_2 | 63 |  | H3K4ME1_HEIGHT_2 | 64 |
| RAD21_HEIGHT_2 | 56 |  | H3K4ME1_SUMMIT_1 | 62 |
| H3K4ME1_HEIGHT_1 | 55 |  | RUNX3_HEIGHT_2 | 55 |
| RUNX3_SUMMIT_1 | 52 |  | POL2_SUMMIT_1 | 54 |
| H3K4ME1_SUMMIT_2 | 50 |  | RAD21_HEIGHT_2 | 51 |
| RUNX3_HEIGHT_2 | 50 |  | H3K4ME1_SUMMIT_2 | 48 |
| H2AZ_SUMMIT_1 | 48 |  | SA1_HEIGHT_2 | 44 |
| RANKING: 15 | |  | RANKING: 16 | |
| RUNX3_HEIGHT_2 | 60 |  | H3K4ME1_HEIGHT_2 | 72 |
| PU.1_HEIGHT_2 | 58 |  | PU.1_HEIGHT_2 | 67 |
| PU.1_SUMMIT_1 | 57 |  | RUNX3_HEIGHT_2 | 65 |
| H3K4ME1_SUMMIT_1 | 55 |  | RUNX3_SUMMIT_1 | 60 |
| H2AZ_HEIGHT_2 | 54 |  | PU.1_SUMMIT_1 | 51 |
| H3K4ME1_HEIGHT_2 | 51 |  | RAD21_HEIGHT_2 | 50 |
| RUNX3_SUMMIT_1 | 50 |  | SA1_HEIGHT_2 | 44 |
| ELF1_SUMMIT_1 | 48 |  | ELF1_HEIGHT_2 | 43 |
| H3K4ME1_SUMMIT_2 | 46 |  | POL2.HAIB_SUMMIT_1 | 42 |
| H2AZ_SUMMIT_1 | 45 |  | POL2_SUMMIT_1 | 41 |
| RANKING: 17 | |  | RANKING: 18 | |
| RUNX3_SUMMIT_1 | 64 |  | RUNX3_SUMMIT_1 | 66 |
| SA1_HEIGHT_2 | 54 |  | PU.1_HEIGHT_2 | 54 |
| RUNX3_HEIGHT_2 | 52 |  | RUNX3_HEIGHT_2 | 54 |
| PU.1_SUMMIT_1 | 50 |  | BCL3_SUMMIT_1 | 48 |
| BCL3_SUMMIT_1 | 48 |  | RAD21_HEIGHT_2 | 48 |
| RAD21_HEIGHT_2 | 47 |  | POL2_HEIGHT_2 | 45 |
| H2AZ_SUMMIT_1 | 46 |  | PU.1_SUMMIT_1 | 44 |
| POL2.4H8_SUMMIT_1 | 46 |  | RAD21_SUMMIT_1 | 43 |
| PU.1_HEIGHT_2 | 43 |  | H2AZ_SUMMIT_1 | 42 |
| H3K4ME1_SUMMIT_2 | 41 |  | POL2_SUMMIT_1 | 41 |
| RANKING: 19 | |  | RANKING: 20 | |
| RAD21_HEIGHT_2 | 65 |  | RAD21_HEIGHT_2 | 55 |
| RUNX3_SUMMIT_1 | 59 |  | RUNX3_HEIGHT_2 | 54 |
| RUNX3_HEIGHT_2 | 54 |  | RUNX3_SUMMIT_1 | 52 |
| SA1_HEIGHT_2 | 53 |  | ELF1_SUMMIT_1 | 45 |
| PU.1_HEIGHT_2 | 48 |  | RAD21.SNYDER_HEIGHT_2 | 44 |
| RAD21.SNYDER_HEIGHT_2 | 47 |  | SA1_HEIGHT_2 | 44 |
| RAD21_SUMMIT_1 | 47 |  | PU.1_SUMMIT_1 | 41 |
| PU.1_SUMMIT_1 | 43 |  | H3K4ME1_HEIGHT_2 | 39 |
| POL2.4H8_HEIGHT_2 | 42 |  | POL2.4H8_HEIGHT_2 | 39 |
| H2AZ_HEIGHT_2 | 40 |  | PU.1_HEIGHT_2 | 39 |
| RANKING: 21 | |  | RANKING: 22 | |
| RUNX3_HEIGHT_2 | 53 |  | RUNX3_SUMMIT_1 | 61 |
| RAD21_HEIGHT_2 | 52 |  | RUNX3_HEIGHT_2 | 60 |
| H3K4ME1_HEIGHT_2 | 47 |  | H2AZ_HEIGHT_2 | 44 |
| RAD21.SNYDER_HEIGHT_2 | 46 |  | RAD21_HEIGHT_2 | 44 |
| RUNX3_SUMMIT_1 | 45 |  | SA1_HEIGHT_2 | 44 |
| SA1_HEIGHT_2 | 45 |  | BCL3_HEIGHT_2 | 43 |
| RAD21_SUMMIT_1 | 44 |  | PU.1_SUMMIT_1 | 39 |
| POL2_HEIGHT_2 | 42 |  | CTCF_HEIGHT_2 | 36 |
| PU.1_HEIGHT_2 | 38 |  | POL2.4H8_SUMMIT_1 | 36 |
| CTCF_HEIGHT_2 | 36 |  | PU.1_HEIGHT_2 | 35 |
| RANKING: 23 | |  | RANKING: 24 | |
| RAD21_HEIGHT_2 | 64 |  | RAD21_HEIGHT_2 | 55 |
| RUNX3_SUMMIT_1 | 54 |  | BCL3_SUMMIT_1 | 50 |
| RUNX3_HEIGHT_2 | 44 |  | RUNX3_SUMMIT_1 | 45 |
| SA1_HEIGHT_2 | 44 |  | RAD21.SNYDER_HEIGHT_2 | 44 |
| BCL3_HEIGHT_2 | 38 |  | RUNX3_HEIGHT_2 | 42 |
| H2AZ_HEIGHT_2 | 38 |  | ELF1_HEIGHT_2 | 41 |
| PU.1_HEIGHT_2 | 37 |  | PU.1_SUMMIT_1 | 40 |
| RAD21.SNYDER_HEIGHT_2 | 36 |  | CTCF_HEIGHT_2.1 | 38 |
| RAD21_SUMMIT_1 | 36 |  | BATF_HEIGHT_2 | 36 |
| SA1_SUMMIT_1 | 36 |  | BCL3_HEIGHT_2 | 35 |
| RANKING: 25 | |  |  |  |
| RUNX3_HEIGHT_2 | 59 |  |  |  |
| RAD21_HEIGHT_2 | 51 |  |  |  |
| RAD21.SNYDER_HEIGHT_2 | 50 |  |  |  |
| RUNX3_SUMMIT_1 | 48 |  |  |  |
| ZNF143_HEIGHT_2 | 46 |  |  |  |
| SA1_HEIGHT_2 | 45 |  |  |  |
| BCL3_SUMMIT_1 | 42 |  |  |  |
| PU.1_HEIGHT_2 | 42 |  |  |  |
| H2AZ_HEIGHT_2 | 41 |  |  |  |
| RAD21_SUMMIT_1 | 39 |  |  |  |

## GM12878, K562, IMR90, HUVEC, NHEK, and HMEC analysis

### Input Features in this test

**Transcription factors**: CTCF.

**Histone Modifications**: H3K27ac, H3K27me3, H3K36me3, H3K4me1, H3K4me2, H3K4me3, H3K9ac, H3K9me3, H4K20me1, H2AZ, and H3k79me2.

See (Supplementary Table 1.xlsx) for full details of all features and their resources.

### Applied Tests

We built one predictor for each TAD, then we tested these predictor on the same TAD within the same cell line (on the testing dataset), and the same TAD but different cell line. The obtained results are reported in the supplementary (Supplementary Table 6.xlsx). The predictors failed in few cases where they predicted only one class. We believe that the better normalization could improve the performance. Or maybe these TADs require more analysis for the genomic and transcription factor patterns.

We illustrate the heatmaps in the Figure 24 for some example domains in all cell lines we included in this test.

|  | |  |  |  | |  |  |  |  |
| --- | --- | --- | --- | --- | --- | --- | --- | --- | --- |
| **chr20:50255531-58072397** | | | | | | | | | |
|  | Full Heatmap | | | | Training Heatmap | | | | Testing Heatamp |
| K562 | 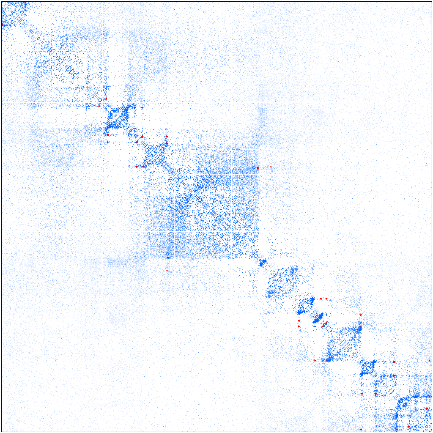 | | | | 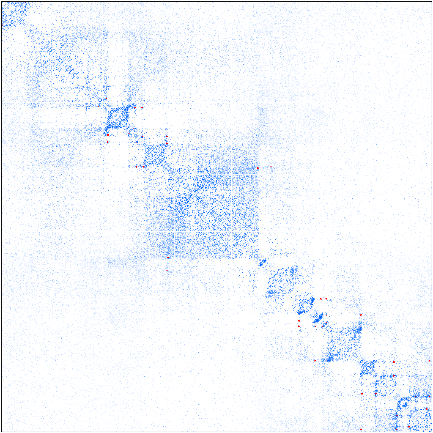 | | | | 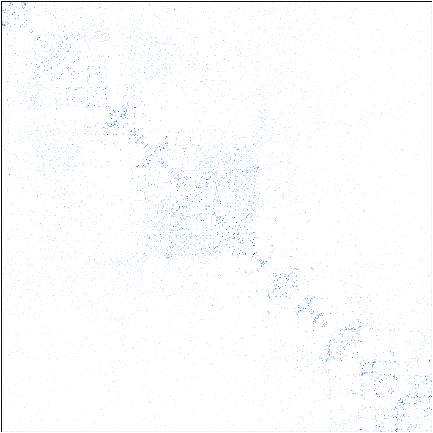 |
| HUVEC | 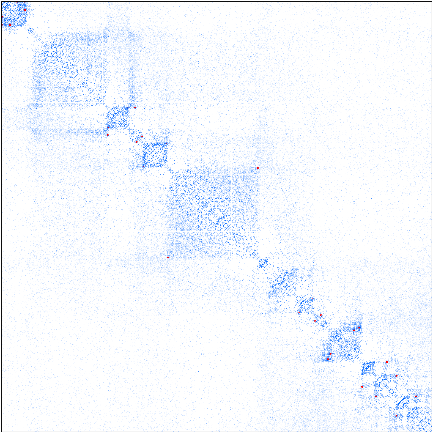 | | | | 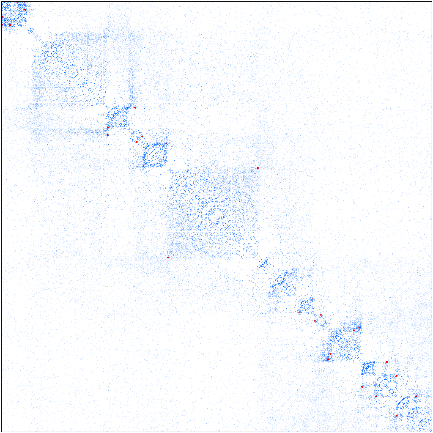 | | | | 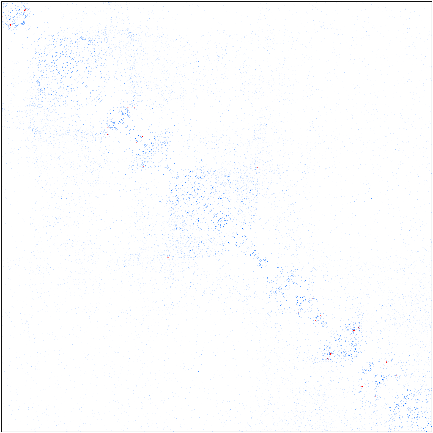 |
| IMR90 | 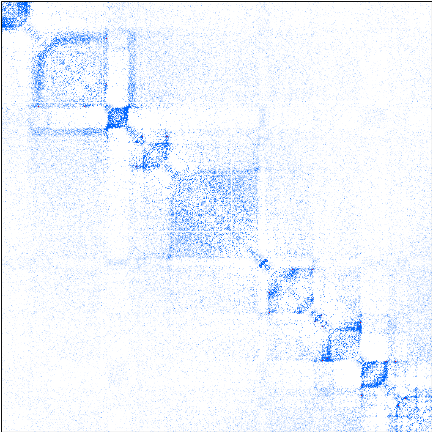 | | | | 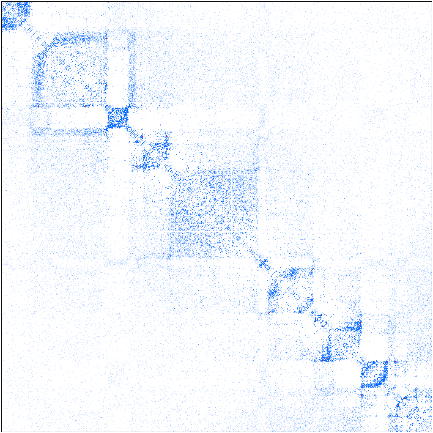 | | | | 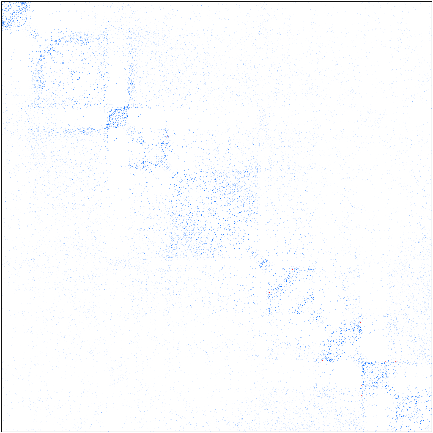 |
| HMEC | 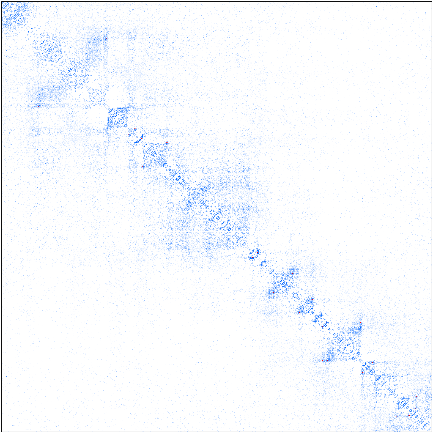 | | | | 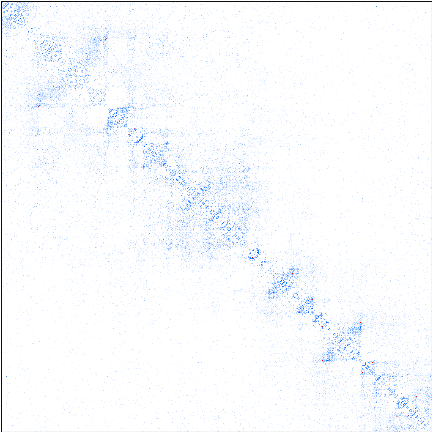 | | | | 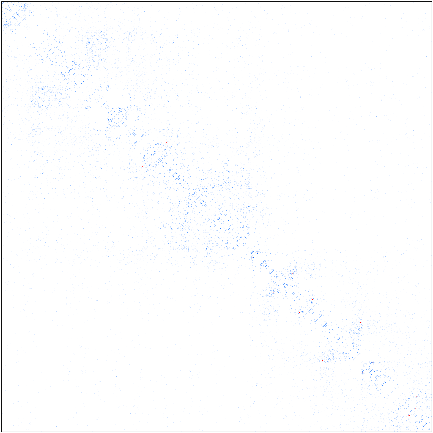 |
| NHEK | 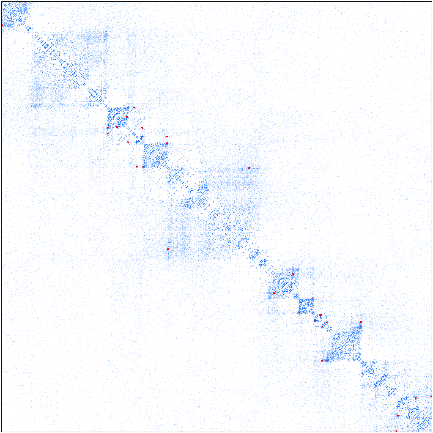 | | | | 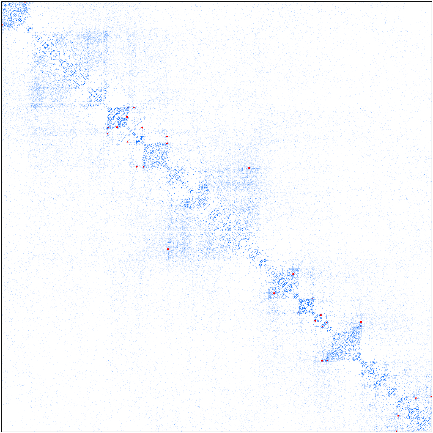 | | | |  |

| **chr10:45425385-52037296** | | | |
| --- | --- | --- | --- |
|  | Full Heatmap | Training heatmap | Testing Heatamp |
| K562 |  |  |  |
| HUVEC |  |  |  |
| IMR90 |  |  |  |
| HMEC |  |  |  |
| NHEK |  |  |  |

Figure 24 the Hi-C heatmaps obtained for K562, HUVEC, HeLa, HMEC, NHEK, and IMR90 when using 0.15 as threshold. Red pixels in the heatmaps represents the physical Hi-C loops, while the black ones represent ChIA-PET Interactions if available.

We illustrate some example heatmaps (the original heatmaps, and the predicted ones) on Figure 25.

|  | Original | Predicted |
| --- | --- | --- |
| GM12878 Chr1:159101161-161110422 predicted by K562 Predictor |  |  |
| K562 Chr1:22065527-24193808 predicted by GM12878 Predictor |  |  |
| K562 Chr6:39315304-43113037 predicted by IMR90 Predictor |  |  |

Figure 25 comparison between the original and predicted heatmap, Purple pixels are wrong predictions (they might be correct interactions according to the other experimental data, their p-values don’t satisfy our condition). Red pixels in the heatmaps representing the physical Hi-C loops, while the black ones represent Hi-C loops.

### Important Features

The most 50 important features, reported for all these cell lines within the TADs are ordered according to their frequencies:

| Order | Feature Name | Count in TADs | Order | Feature Name | Count in TADs |
| --- | --- | --- | --- | --- | --- |
| 1 | distance | 13433 | **25** | H3K4ME3_HEIGHT_2 | 7608 |
| 2 | H3K4ME1_SUMMIT_1 | 13367 | **26** | H3K9AC_SUMMIT_1 | 5687 |
| 3 | H3K4ME1_HEIGHT_2 | 13360 | **27** | H3K9AC_HEIGHT_2 | 5042 |
| 4 | H2AZ_SUMMIT_1 | 13312 | **28** | H3K36ME3_SUMMIT_2 | 4880 |
| 5 | H2AZ_HEIGHT_2 | 13278 | **29** | H3K4ME2_SUMMIT_2 | 4615 |
| 6 | H3K4ME2_SUMMIT_1 | 12836 | **30** | H3K36ME3_HEIGHT_1 | 4391 |
| 7 | H3K4ME2_HEIGHT_2 | 12604 | **31** | H3K4ME2_HEIGHT_1 | 4288 |
| 8 | CTCF_HEIGHT_2 | 12547 | **32** | H4K20ME1_SUMMIT_1 | 4270 |
| 9 | CTCF_SUMMIT_1 | 12331 | **33** | H4K20ME1_HEIGHT_2 | 3874 |
| 10 | H3K4ME1_SUMMIT_2 | 11108 | **34** | H3K79ME2_SUMMIT_2 | 3622 |
| 11 | H3K4ME1_HEIGHT_1 | 10835 | **35** | H3K79ME2_HEIGHT_1 | 3593 |
| 12 | H3K27AC_SUMMIT_1 | 10405 | **36** | H3K27ME3_SUMMIT_2 | 3535 |
| 13 | H3K36ME3_SUMMIT_1 | 10218 | **37** | CTCF_HEIGHT_1 | 3386 |
| 14 | H3K36ME3_HEIGHT_2 | 10132 | **38** | CTCF_SUMMIT_2 | 3291 |
| 15 | H3K27AC_HEIGHT_2 | 9953 | **39** | H3K27ME3_HEIGHT_1 | 3132 |
| 16 | H3K79ME2_SUMMIT_1 | 9876 | **40** | H3K9ME3_SUMMIT_2 | 2377 |
| 17 | H3K27ME3_SUMMIT_1 | 9781 | **41** | H3K9ME3_HEIGHT_1 | 2233 |
| 18 | H3K79ME2_HEIGHT_2 | 9732 | **42** | H3K4ME3_HEIGHT_1 | 1022 |
| 19 | H3K27ME3_HEIGHT_2 | 9488 | **43** | H3K4ME3_SUMMIT_2 | 897 |
| 20 | H2AZ_SUMMIT_2 | 8947 | **44** | H4K20ME1_SUMMIT_2 | 785 |
| 21 | H2AZ_HEIGHT_1 | 8361 | **45** | H3K27AC_SUMMIT_2 | 748 |
| 22 | H3K4ME3_SUMMIT_1 | 8321 | **46** | H3K27AC_HEIGHT_1 | 730 |
| 23 | H3K9ME3_SUMMIT_1 | 8221 | **47** | H4K20ME1_HEIGHT_1 | 691 |
| 24 | H3K9ME3_HEIGHT_2 | 7888 | **48** | H3K9AC_HEIGHT_1 | 500 |
|  |  |  | **49** | H3K9AC_SUMMIT_2 | 319 |

## Human population analysis

### Input Features in this test

**Transcription Factors**: CTCF, and SA1.

**Histone modifications**: H3K4me1, H3K4me3, H3K27ac, H3K27me3, and H3K36me3.

**Cell Lines**: In total 8 individuals from 1000 Genomes Project: GM12878, HG00731, HG00732, HG00513, HG00514, and the trio (family) GM19238, GM19239, and GM19240. All lymphoblastoid cell lines.

### Tests

We used experimental heatmaps with 40kb resolution, which is the highest resolution data we could obtain.

We build one predictor for each TAD, then we trained the predictor on each cell type and tested it on the rest of the predictors. The genomics segments were divided into 40kb resolution to match the resolution of the experimental heatmaps. The full results were described in the supplementary (Supplementary Table 6.xlsx).

### Important Features

The top 10 important features for all individuals are reported below in the Table 12 with their frequencies in each ranking. The first row in the ranking 1 means that the distance got the ranking one over the important features in 11683 tests.

| Ranking 1 | | Ranking 2 | |
| --- | --- | --- | --- |
| distance | 11683 | CTCF_Height_2 | 1849 |
| CTCF_Height_2 | 547 | CTCF_Summit_2 | 1685 |
| CTCF_Summit_2 | 424 | SA1_Height_2 | 1299 |
| SA1_Height_2 | 341 | SA1_Summit_2 | 1120 |
| SA1_Summit_2 | 312 | H3k4me1_Height_2 | 1081 |
| H3k4me1_Height_1 | 309 | H3k4me1_Height_1 | 1007 |
| H3k4me1_Summit_1 | 267 | distance | 991 |
| H3k4me1_Height_2 | 240 | H3k4me1_Summit_2 | 972 |
| H3k4me1_Summit_2 | 222 | H3k4me1_Summit_1 | 782 |
| H3k4me3_Height_2 | 180 | H3k4me3_Height_2 | 486 |
| H3k36me3_Summit_2 | 163 | H3k36me3_Summit_2 | 480 |
| H3k27me3_Summit_2 | 144 | H3k27me3_Summit_2 | 446 |
| H3k4me3_Summit_2 | 142 | H3k36me3_Height_2 | 440 |
| H3k36me3_Height_2 | 140 | H3k27me3_Height_2 | 430 |
| H3k27me3_Height_2 | 134 | H3k27ac_Summit_2 | 411 |
| CTCF_Summit_1 | 131 | H3k27ac_Height_2 | 403 |
| CTCF_Height_1 | 130 | H3k4me3_Summit_2 | 394 |
| H3k27ac_Height_2 | 121 | CTCF_Summit_1 | 281 |
| H3k27ac_Summit_2 | 115 | CTCF_Height_1 | 262 |
| H3k27ac_Height_1 | 104 | H3k36me3_Height_1 | 238 |
| SA1_Summit_1 | 86 | SA1_Summit_1 | 216 |
| SA1_Height_1 | 84 | H3k36me3_Summit_1 | 200 |
| H3k36me3_Height_1 | 68 | H3k27ac_Summit_1 | 196 |
| H3k36me3_Summit_1 | 67 | H3k27ac_Height_1 | 181 |
| H3k27ac_Summit_1 | 64 | SA1_Height_1 | 175 |
| H3k4me3_Height_1 | 50 | H3k27me3_Summit_1 | 94 |
| H3k4me3_Summit_1 | 46 | H3k4me3_Height_1 | 92 |
| H3k27me3_Summit_1 | 32 | H3k4me3_Summit_1 | 79 |
| H3k27me3_Height_1 | 22 | H3k27me3_Height_1 | 78 |

| Ranking 3 | | Ranking 4 | |
| --- | --- | --- | --- |
| CTCF_Height_2 | 1762 | SA1_Height_2 | 1586 |
| CTCF_Summit_2 | 1533 | CTCF_Height_2 | 1534 |
| SA1_Height_2 | 1350 | SA1_Summit_2 | 1485 |
| SA1_Summit_2 | 1190 | CTCF_Summit_2 | 1414 |
| H3k4me1_Summit_2 | 1008 | H3k4me1_Summit_2 | 1029 |
| H3k4me1_Height_2 | 991 | H3k4me1_Height_2 | 1014 |
| distance | 899 | H3k4me1_Height_1 | 663 |
| H3k4me1_Summit_1 | 812 | H3k4me1_Summit_1 | 622 |
| H3k4me1_Height_1 | 801 | H3k27ac_Height_2 | 571 |
| H3k27me3_Height_2 | 507 | H3k27ac_Summit_2 | 533 |
| H3k36me3_Summit_2 | 504 | distance | 508 |
| H3k27ac_Height_2 | 492 | H3k4me3_Summit_2 | 487 |
| H3k36me3_Height_2 | 490 | H3k4me3_Height_2 | 486 |
| H3k27me3_Summit_2 | 485 | H3k36me3_Height_2 | 467 |
| H3k4me3_Height_2 | 443 | H3k36me3_Summit_2 | 466 |
| H3k27ac_Summit_2 | 431 | H3k27me3_Height_2 | 457 |
| H3k4me3_Summit_2 | 394 | H3k27me3_Summit_2 | 436 |
| CTCF_Summit_1 | 306 | CTCF_Height_1 | 383 |
| CTCF_Height_1 | 290 | CTCF_Summit_1 | 319 |
| H3k36me3_Height_1 | 236 | H3k27ac_Height_1 | 309 |
| H3k27ac_Height_1 | 223 | H3k27ac_Summit_1 | 285 |
| H3k27ac_Summit_1 | 217 | H3k36me3_Summit_1 | 250 |
| SA1_Summit_1 | 214 | SA1_Summit_1 | 237 |
| SA1_Height_1 | 214 | SA1_Height_1 | 229 |
| H3k36me3_Summit_1 | 214 | H3k36me3_Height_1 | 207 |
| H3k4me3_Summit_1 | 107 | H3k4me3_Height_1 | 131 |
| H3k4me3_Height_1 | 105 | H3k4me3_Summit_1 | 121 |
| H3k27me3_Height_1 | 86 | H3k27me3_Summit_1 | 79 |
| H3k27me3_Summit_1 | 64 | H3k27me3_Height_1 | 60 |

| Ranking 5 | | Ranking 6 | |
| --- | --- | --- | --- |
| SA1_Height_2 | 1529 | H3k4me1_Summit_2 | 1333 |
| CTCF_Summit_2 | 1445 | H3k4me1_Height_2 | 1244 |
| SA1_Summit_2 | 1435 | SA1_Height_2 | 1187 |
| CTCF_Height_2 | 1343 | SA1_Summit_2 | 1179 |
| H3k4me1_Height_2 | 1136 | CTCF_Height_2 | 1148 |
| H3k4me1_Summit_2 | 1073 | CTCF_Summit_2 | 1119 |
| H3k4me1_Summit_1 | 622 | H3k27ac_Summit_2 | 700 |
| H3k4me1_Height_1 | 621 | H3k27ac_Height_2 | 683 |
| H3k27ac_Height_2 | 590 | CTCF_Height_1 | 619 |
| H3k27ac_Summit_2 | 563 | H3k4me3_Height_2 | 608 |
| H3k4me3_Summit_2 | 556 | H3k4me1_Height_1 | 581 |
| H3k4me3_Height_2 | 524 | H3k4me3_Summit_2 | 573 |
| H3k36me3_Summit_2 | 481 | H3k4me1_Summit_1 | 554 |
| distance | 464 | H3k36me3_Summit_2 | 545 |
| H3k27me3_Height_2 | 456 | CTCF_Summit_1 | 538 |
| H3k36me3_Height_2 | 447 | H3k27me3_Summit_2 | 530 |
| H3k27me3_Summit_2 | 444 | H3k27me3_Height_2 | 506 |
| CTCF_Height_1 | 380 | H3k36me3_Height_2 | 474 |
| CTCF_Summit_1 | 349 | SA1_Height_1 | 311 |
| H3k27ac_Height_1 | 299 | SA1_Summit_1 | 288 |
| H3k27ac_Summit_1 | 281 | H3k27ac_Summit_1 | 271 |
| SA1_Height_1 | 246 | distance | 266 |
| SA1_Summit_1 | 237 | H3k27ac_Height_1 | 264 |
| H3k36me3_Height_1 | 231 | H3k36me3_Height_1 | 212 |
| H3k36me3_Summit_1 | 222 | H3k36me3_Summit_1 | 205 |
| H3k4me3_Summit_1 | 134 | H3k4me3_Summit_1 | 142 |
| H3k4me3_Height_1 | 108 | H3k4me3_Height_1 | 141 |
| H3k27me3_Height_1 | 85 | H3k27me3_Summit_1 | 87 |
| H3k27me3_Summit_1 | 67 | H3k27me3_Height_1 | 60 |

| Ranking 7 | | Ranking 8 | |
| --- | --- | --- | --- |
| H3k4me1_Summit_2 | 1336 | H3k4me1_Height_2 | 1181 |
| H3k4me1_Height_2 | 1303 | H3k4me1_Summit_2 | 1145 |
| SA1_Height_2 | 1194 | SA1_Height_2 | 967 |
| SA1_Summit_2 | 1169 | SA1_Summit_2 | 966 |
| CTCF_Summit_2 | 1033 | CTCF_Summit_2 | 959 |
| CTCF_Height_2 | 988 | H3k27ac_Height_2 | 916 |
| H3k27ac_Height_2 | 738 | H3k27ac_Summit_2 | 906 |
| H3k27ac_Summit_2 | 713 | CTCF_Height_2 | 883 |
| H3k4me3_Height_2 | 630 | H3k4me3_Height_2 | 733 |
| CTCF_Summit_1 | 628 | CTCF_Height_1 | 707 |
| H3k4me3_Summit_2 | 626 | H3k4me1_Height_1 | 687 |
| CTCF_Height_1 | 624 | CTCF_Summit_1 | 640 |
| H3k4me1_Height_1 | 540 | H3k4me3_Summit_2 | 638 |
| H3k4me1_Summit_1 | 532 | H3k4me1_Summit_1 | 538 |
| H3k36me3_Height_2 | 530 | H3k36me3_Height_2 | 529 |
| H3k27me3_Summit_2 | 525 | H3k27me3_Height_2 | 516 |
| H3k27me3_Height_2 | 494 | H3k36me3_Summit_2 | 513 |
| H3k36me3_Summit_2 | 490 | H3k27me3_Summit_2 | 503 |
| SA1_Summit_1 | 353 | SA1_Summit_1 | 467 |
| SA1_Height_1 | 347 | SA1_Height_1 | 429 |
| H3k27ac_Height_1 | 261 | H3k27ac_Height_1 | 265 |
| H3k27ac_Summit_1 | 239 | H3k27ac_Summit_1 | 250 |
| distance | 212 | H3k36me3_Summit_1 | 201 |
| H3k36me3_Height_1 | 209 | H3k36me3_Height_1 | 196 |
| H3k36me3_Summit_1 | 204 | distance | 179 |
| H3k4me3_Summit_1 | 155 | H3k4me3_Height_1 | 137 |
| H3k4me3_Height_1 | 117 | H3k4me3_Summit_1 | 118 |
| H3k27me3_Height_1 | 95 | H3k27me3_Summit_1 | 111 |
| H3k27me3_Summit_1 | 83 | H3k27me3_Height_1 | 88 |

| Ranking 9 | | Ranking 10 | |
| --- | --- | --- | --- |
| H3k4me1_Height_2 | 1224 | H3k27ac_Height_2 | 978 |
| H3k4me1_Summit_2 | 1106 | H3k4me1_Summit_2 | 971 |
| H3k27ac_Height_2 | 995 | H3k27ac_Summit_2 | 955 |
| H3k27ac_Summit_2 | 959 | H3k4me1_Height_2 | 930 |
| SA1_Summit_2 | 935 | H3k4me3_Summit_2 | 874 |
| SA1_Height_2 | 917 | SA1_Summit_2 | 857 |
| CTCF_Summit_2 | 857 | H3k4me3_Height_2 | 855 |
| CTCF_Height_2 | 849 | CTCF_Summit_2 | 794 |
| H3k4me3_Height_2 | 755 | H3k4me1_Height_1 | 779 |
| H3k4me3_Summit_2 | 697 | SA1_Height_2 | 775 |
| CTCF_Height_1 | 695 | CTCF_Height_1 | 762 |
| CTCF_Summit_1 | 653 | CTCF_Summit_1 | 727 |
| H3k4me1_Height_1 | 583 | CTCF_Height_2 | 709 |
| H3k4me1_Summit_1 | 563 | H3k4me1_Summit_1 | 659 |
| H3k36me3_Height_2 | 560 | H3k36me3_Height_2 | 569 |
| H3k36me3_Summit_2 | 521 | H3k36me3_Summit_2 | 550 |
| H3k27me3_Summit_2 | 512 | SA1_Height_1 | 525 |
| H3k27me3_Height_2 | 508 | SA1_Summit_1 | 523 |
| SA1_Height_1 | 494 | H3k27me3_Height_2 | 495 |
| SA1_Summit_1 | 431 | H3k27me3_Summit_2 | 481 |
| H3k27ac_Height_1 | 285 | H3k27ac_Summit_1 | 276 |
| H3k27ac_Summit_1 | 227 | H3k27ac_Height_1 | 272 |
| H3k36me3_Height_1 | 202 | H3k36me3_Height_1 | 214 |
| H3k4me3_Summit_1 | 187 | H3k36me3_Summit_1 | 185 |
| H3k36me3_Summit_1 | 184 | H3k4me3_Height_1 | 178 |
| H3k4me3_Height_1 | 160 | H3k4me3_Summit_1 | 142 |
| distance | 143 | distance | 116 |
| H3k27me3_Summit_1 | 84 | H3k27me3_Height_1 | 111 |
| H3k27me3_Height_1 | 82 | H3k27me3_Summit_1 | 106 |

Table 12 The important features in the top 10 rankings, with the frequency for each feature in the specified ranking

# Features Distribution

First, we report the feature distribution at the interactions anchors according to the different interaction types (CTCF, RNAPII ChIA-PET, and Hi-C loops). We also report the same analysis for comparison of the anchors from our predicted interactions and non-interacting genomic segments.

## K562 Cell Line

Figure 26. K562 Features Distribution in the interaction anchors reported for three different interactions types: CTCF ChIA-PET in red, Hi-C Loops in green, and RNAP II ChIA-PET in blue.

Figure 27. K562 Features Distribution in our CTCF ChIA-PET Predicted Interactions reported for the anchors of our predicted interactions and the non-interacting segments. Our prediction are in red and the non-interacting segments are in green.

Figure 28. K562 Features Distribution in our Hi-C Loops Predicted Interactions reported for the anchors of our predicted interactions and the non-interacting segments. Our prediction is in red and the non-interacting segments are in green.

Figure 29. K562 Features Distribution in our RNAP II ChIA-PET Predicted Interactions reported for the anchors of our predicted interactions and the non-interacting segments. Our prediction are in red and the non-interacting segments are in green.

## HeLa Cell Line

Figure 30. HeLa Features Distribution in the interaction anchors reported for three different interactions types CTCF ChIA-PET in red, Hi-C Loops in green, and RNAP II ChIA-PET in blue.

Figure 31. HeLa Features Distribution in our CTCF ChIA-PET Predicted Interactions reported for the anchors of our predicted interactions and the non-interacting segments. Our prediction is in red and the non-interacting segments are in green.

Figure 32. HeLa Features Distribution in our Hi-C loops Predicted Interactions reported for the anchors of our predicted interactions and the non-interacting segments. Our prediction is in red and the non-interacting segments are in green.

Figure 33. HeLa Features Distribution in our RNAP II ChIA-PET Predicted Interactions reported for the anchors of our predicted interactions and the non-interacting segments. Our prediction is in red and the non-interacting segments are in green.

# Parameters and Configuration

## MACS peak Calling

We use MACS2 for peaks calling as follows:

macs2 callpeak -t assay_bed_file -g hs --nomodel --nolambda -n output_name

## Random Forests Classifier

We use Random forests with the default parameters for binary classification.

The parameters are as follows:

Number of trees =500, Node Size = 1, Number of random variables at each split = square root of the features so it depends on the tests, and Cutoff = 0.5.
